# The perineurium integrates leptin with its sympathetic outflow to protect against obesity

**DOI:** 10.1038/s42255-026-01555-3

**Published:** 2026-07-13

**Authors:** Gitalee Sarker, Emma Haberman, Anandhakumar Chandran, Thomas Monfeuga, Cyrielle Maroteau, Sofia Lundh, David S. Oliveira, Andrea Raimondi, Karin Ziegler, Tiago Gomes, Tiago N. Cordeiro, Karishma Parekh, Emilia Schmid, Paola Fernández-Sanmartín, Alina Griebel, Noelia Martinez-Sanchez, Bernardo A. Arús, Samson W. Cheung, Yitao Zhu, Michal Shaked, Sarah C. L. Knowles, Stefan Hanns Engelhardt, Matteo Iannacone, Philipp E. Scherer, Miguel López, Enrique M. Toledo, Ana I. Domingos

**Affiliations:** 1 Department of Physiology, Anatomy and Genetics, University of Oxford, Oxford, UK; 2 Research and Development, Novo Nordisk Research Centre Oxford, Oxford, UK; 3 Department of Pathology and Imaging, Global Drug Discovery, Novo Nordisk, Måløv, Denmark; 4 Experimental Imaging Centre, IRCCS San Raffaele Scientific Institute, Milan, Italy; 5 Institute of Pharmacology and Toxicology, Technical University Munich (TUM), Munich, Germany; 6 DZHK (German Centre for Cardiovascular Research), Partner Site Munich Heart Alliance, Munich, Germany; 7 Instituto de Tecnologia Química e Biológica António Xavier, ITQB NOVA, Lisbon, Portugal; 8 Neurobesity Group, Department of Physiology, Center for Research in Molecular Medicine and Chronic Diseases (CiMUS), Universidade de Santiago de Compostela, Santiago, Spain; 9 Oxford Centre for Diabetes, Endocrinology and Metabolism, Oxford, UK; 10 Helmholtz-Zentrum Dresden-Rossendorf (HZDR), Dresden, Germany; 11 Medizinische Fakultät and University Hospital Carl Gustav Carus, Technische Universität Dresden, Dresden, Germany; 12 Department of Functional Imaging in Surgical Oncology, National Center for Tumor Diseases (NCT/UCC), Dresden, Germany; 13 German Cancer Research Center (DKFZ), Heidelberg, Germany; 14 Department of Biology, University of Oxford, Oxford, UK; 15 Vita-Salute San Raffaele University, Milan, Italy; 16 Division of Immunology, Transplantation and Infectious Diseases, IRCCS San Raffaele Scientific Institute, Milan, Italy; 17 Touchstone Diabetes Center, The University of Texas Southwestern Medical Center, Dallas, TX USA

**Keywords:** Molecular biology, Neuroscience, Metabolism

## Abstract

The regulatory mechanism of leptin’s afferent action in the brain is contingent upon the efferent sympathetic innervation of white and brown adipose tissues. Nonetheless, the peripheral regulation governing the afferent–efferent balance remains ambiguous. Here we show the enriched expression of both leptin receptor (*Lepr*) and β_2_-adrenergic receptor (*Adrb2*) in perineurial cells that form a barrier around sympathetic ganglia and nerve bundles in adipose tissues, using single-cell RNA sequencing on mouse sympathetic ganglia. *Lepr*^+^ sympathetic perineurial cells (SPCs) are molecularly similar to endothelial cells. Conditional knockout of *Adrb2* in *Lepr*^+^ cells, including SPCs, predisposes male mice to obesity by lowering energy expenditure and thermogenesis without affecting food intake. Notably, obesity-associated hyperleptinaemia causes apoptosis in SPCs, disrupting the perineurial barrier and concomitant adipose sympathetic neuropathy. This deleterious effect can be reversed by partial reduction of leptin or sympathomimetic β_2_-adrenergic receptor agonism. Clinically, we observed a male-specific synergistic effect of *LEPR* and *ADRB2* polymorphisms on increased body mass index risk in a large European population. We propose that SPCs coordinate the afferent and efferent arms of the neuroendocrine loop of leptin action to regulate energy expenditure and body weight.

## Abstract (web summary)

*Lepr*^+^*Adrb2*^+^ sympathetic perineurial cells coordinate leptin signalling and sympathetic output to modulate energy expenditure, thermogenesis and body weight in male mice, independent of changes to food intake.

## Main

Leptin regulates body weight via a neuroendocrine negative feedback loop. The hormone leptin is the afferent signal released from white adipocytes, in proportion to fat reserves, and acts on hypothalamic neurons to suppress food intake and activate proportional descending efferent sympathetic activity that triggers lipolysis in white adipose tissue (WAT)^1^ and thermogenesis in brown adipose tissue (BAT)^2–4^. The efferent arm reduces mass via noradrenergic signalling, which in turn reduces leptin levels, thereby closing the loop. However, the cellular and molecular mechanisms regulating the balance between afferent leptin and efferent sympathetic outflow are unknown. Here, we identified the sympathetic perineurium as a key player in the neuroendocrine loop of leptin action, regulating the balance between the afferent and efferent arms to control body weight. Sympathetic perineurial cells (SPCs) show enriched expression of both the leptin receptor (*Lepr*) and beta-2 adrenoceptor (*Adrb2*), enabling them to sense the balance of the afferent and efferent arms in the neuroendocrine loop of leptin action. High levels of leptin drive apoptosis of SPCs, which can be prevented by sympathomimetic β_2_-adrenergic receptor agonism. The strength of leptin’s efferent sympathetic output is determined by the apoptosis of SPCs and the integrity of the perineurial barrier, which has been shown to have a neuroprotective role^5^. This working model could explain why humans with common polymorphisms of both *LEPR* and *ADRB2* have a higher risk of developing obesity and provide a peripheral mechanistic model for the leptin-regulated energy balance beyond the central appetite regulation.

## Results

### scRNA-seq of sympathetic ganglia reveals a cluster of endothelial cells highly expressing the leptin receptor

**Fig. 1:**
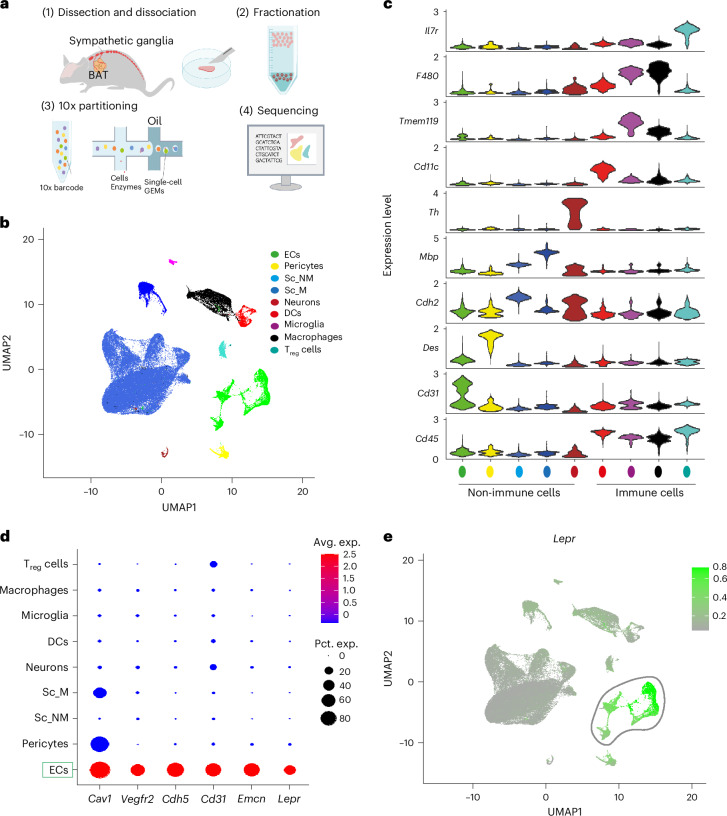
Single-cell transcriptomic data of sympathetic ganglia reveal an endothelial population that highly expresses leptin receptor. **a**, Schematic workflow of scRNA-seq of mouse sympathetic ganglia. **b**, UMAP plot of all 73,042 cells from mouse superior cervical and stellate ganglia. **c**, Violin plots showing the expression of cell-type-specific marker genes. The *y* axis indicates the log-normalized gene expression value, and the width indicates the number of cells expressing the particular gene. **d**, Dot plot showing the expression of endothelial markers (*Cav1*, *Vegfr2*, *Cdh5*, *Cd31*, *Emcn*) and leptin receptor (*Lepr*) in the endothelial cell population. The size of the dot corresponds to the percentage of cells expressing the gene in each cluster, and the colour represents the average gene expression level. **e**, Feature plot showing the expression of *Lepr* in the endothelial cluster. For visualization, denoised expression data were used (**c** and **e**). DC, dendritic cell; EC, endothelial cell; GEMs, gel beads in emulsion; Sc_NM, Schwann cell_non myelinating; Sc_M, Schwann cell_myelinating; T_reg_ cell, regulatory T cell; avg., average; exp., expression; pct., percentage. Source data

**Extended Data Fig. 1:**
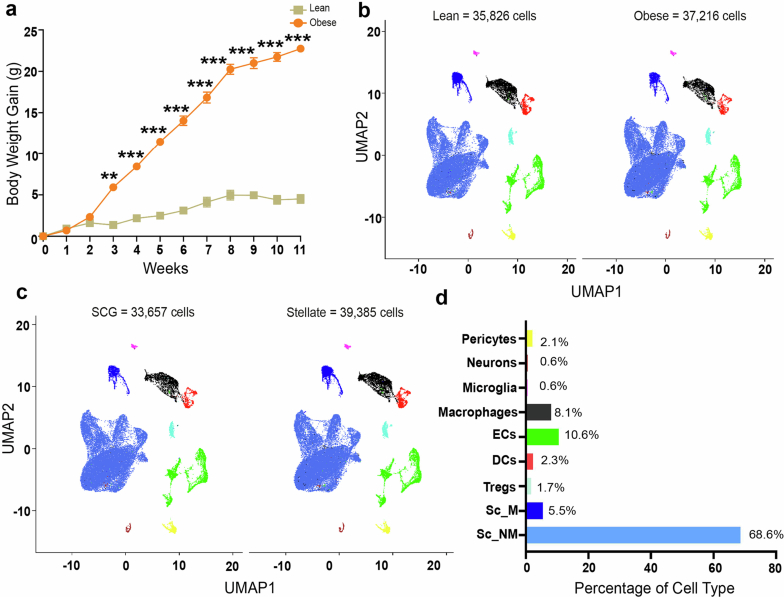
Single-cell transcriptomic data of sympathetic ganglia from lean and obese mice. **a**, The weekly body weight gain of WT male mice treated with normal chow diet (lean) and a high-fat diet (obese) for 11 weeks (n = 12 per group). p values by week: 0 (3.6 × 10^−5^), 1 (2.3 × 10^−5^), 2 (7.69 × 10^−4^), 3 (0.36618), 4 (0.012173), 5 (2.6 × 10^−5^), 6 (4.0 × 10^−6^), 7 ( < 1 × 10^−6^), 8 ( < 1 × 10^−6^), 9 ( < 1 × 10^−6^), 10 ( < 1 × 10^−6^), 11 ( < 1 × 10^−6^). **b**, Uniform manifold approximation and projection (UMAP) plot indicating sample classification as lean and obese. **c**, Uniform manifold approximation and projection (UMAP) plot indicating sample classification as superior cervical ganglia (SCG) and stellate. **d**, Bar graph showing the percentage of different cell populations. ECs = Endothelial cells, Sc_NM = Schwann cell_non myelinating, Sc_M = Schwann cell_myelinating, DCs = dendritic cells, Tregs = regulatory T cells. Data were mean ± s.e.m and were analyzed using two-way ANOVA with Turkey’s post-hoc test. **p < 0.01, ***p < 0.001. Source data

To map the heterogeneity of non-neuronal cell types resident in sympathetic ganglia and their plasticity in response to obesity, we performed single-cell RNA sequencing (scRNA-seq) on isolated superior cervical ganglia (SCG) and stellate ganglia from male C57BL/6 mice fed either a chow diet or a high-fat diet (HFD) for 12 weeks. As expected, the HFD-fed mice gained substantially more weight compared with the chow-fed mice (Extended Data Fig. 1a). For each condition, we pooled SCG or stellate ganglia from lean and obese mice before scRNA-seq, and the four datasets were integrated for analysis (Fig. 1a). After low-quality filtering of the scRNA-seq data, we considered a total of 73,042 single cells. After data integration, we performed principal component analysis and dimensionality reduction with uniform manifold approximation and projection (UMAP) in the integrated dataset^6^. Using unsupervised clustering, we detected nine distinct clusters of immune and non-immune cells (Fig. 1b). Each cluster contained cells from each ganglia type, feeding condition and sample batch, indicating that the transcriptional identities of these cell clusters are stable irrespective of experimental conditions (Extended Data Fig. 1b,c). An immune cell marker (*Cd45*) was expressed in all immune cell populations and absent in non-immune cell populations (Fig. 1c). Using expression patterns of cell-type-specific marker genes, we assigned a single identity to each cluster: endothelial cells (*Cd31*^*+*^), pericytes (*Des*^*+*^), non-myelinating Schwann cells (*Cdh2*^+^), myelinating Schwann cells (*Mbp*^+^), neurons (*Th*^+^), dendritic cells (*Cd11c*^*+*^), microglia (*Tmem11*^*+*^), macrophages (*F480*^*+*^) and regulatory T cells (*Il17r*^*+*^; Fig. 1c). Overall, 74% of the single-cell transcriptomes were assigned to glial cells, 10.6% to endothelial cells, 2.1% to pericytes, 0.6% to neurons and 12.7% to immune cell types (Extended Data Fig. 1d).

**Extended Data Fig. 2:**
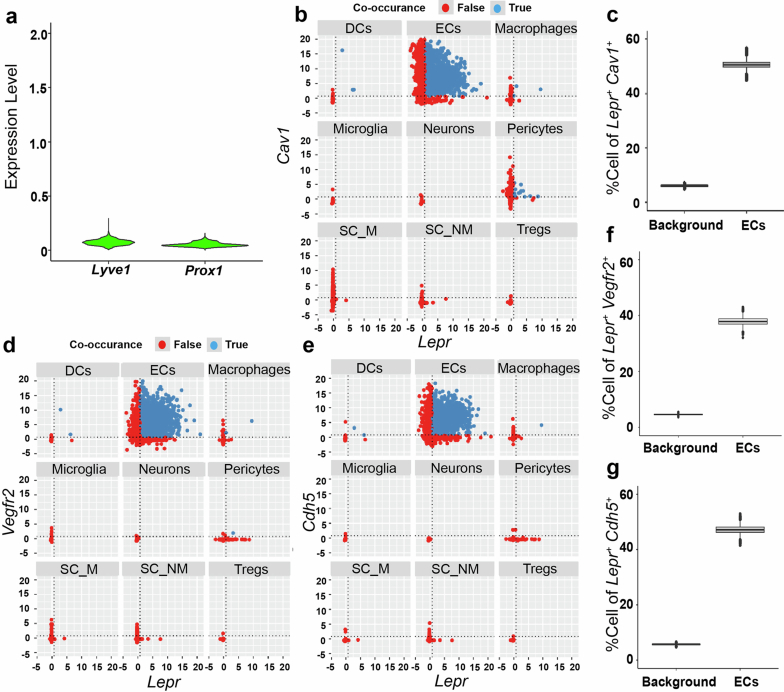
Identification of *Lepr*^*+*^ vascular endothelial cell population in sympathetic ganglia. **a**, Violin plot to show *Lyve1* and *Prox1* expression in sympathetic endothelial cell population. For visualization, denoised expression data was used. **b**, Correlogram to show the co-expression of *Lepr* and *Cav1* in different cell populations. **c**, Box plot showing the percentage of cells co-expressing *Lepr* and *Cav1* in the endothelial cluster from scRNA-seq data of 20 lean mice (10 per experiment). Values were estimated by bootstrap resampling of pooled cells. Boxes indicate median and interquartile range of bootstrap distributions. **d**, Correlogram to show the co-expression of *Lepr* and *Vegfr2* in different cell populations. **e**, Correlogram to show the co-expression of *Lepr* and *Cdh5* in different cell populations. **f**, Box plot showing the percentage of *Lepr*^*+*^*Vegfr2*^*+*^ in the endothelial cell population from scRNA-seq data of 20 lean mice (10 per experiment). Values were estimated by bootstrap resampling of pooled cells. Boxes indicate median and interquartile range of bootstrap distributions. **g**, Box plot shows the percentage of cells co-expressing *Lepr* and *Cdh5* in the endothelial cluster from scRNA-seq data of 20 lean mice (10 per experiment). Values were estimated by bootstrap resampling of pooled cells. Boxes indicate median and interquartile range of bootstrap distributions. The position of the dotted lines is described in the Methods. ECs = endothelial cells, Sc_NM = Schwann cell_non myelinating, Sc_M = Schwann cell_myelinating, DCs = dendritic cells, Tregs = regulatory T cells. Source data

**Extended Data Fig. 3:**
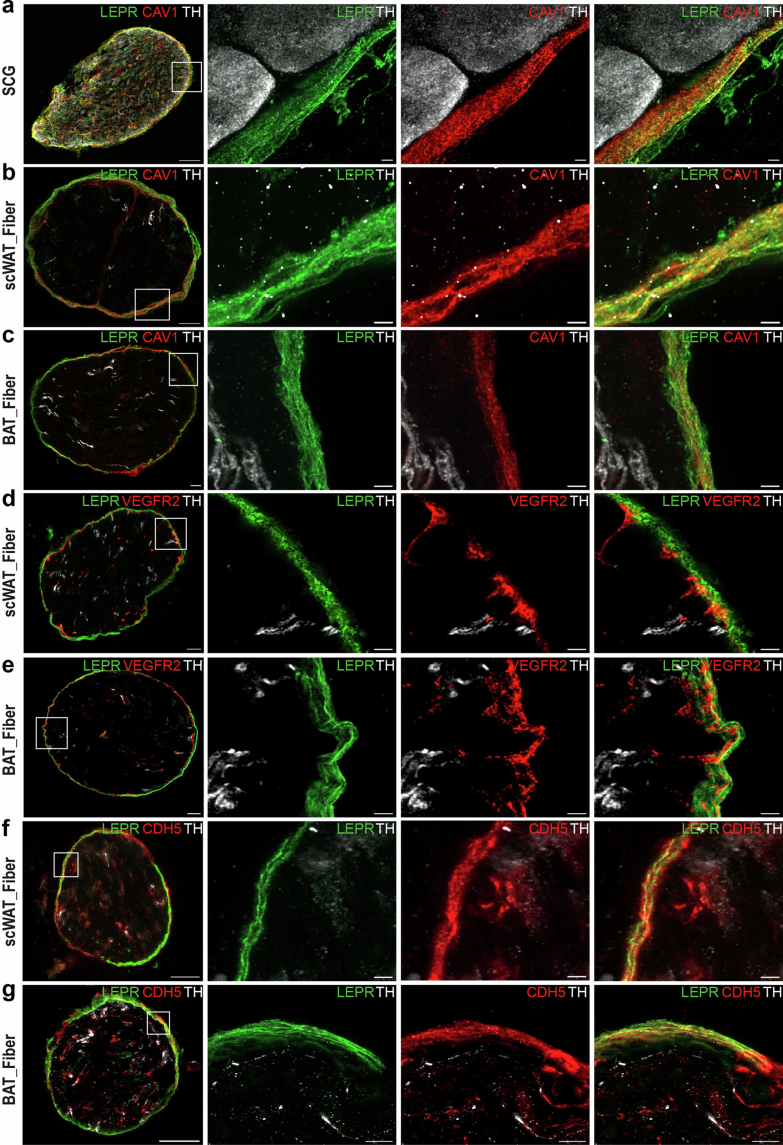
*Lepr*^+^ sympathetic endothelial cells constitute a thin layer surrounding SCG and sympathetic nerve bundles of scWAT and BAT isolated from 12-week-old *Lepr*^Cre^; LSL-YFP mice. **a-c**, Co-expression of LEPR (YFP) & CAV1 in the perineurium surrounding the TH^+^ sympathetic neurons. **d, e** Co-expression of LEPR (YFP) & VEGFR2 in the perineurium surrounding the TH^+^ sympathetic neurons. **f, g** Co-expression of LEPR (YFP) & CDH5 in the perineurium surrounding the TH^+^ sympathetic neurons. Samples were imaged at 40x MPLX_4Y Airyscan mode (**a-g**, left panel). Scale bar = SCG (50 µm), nerve bundles (10 µm). The white box indicates the field of view of the high-resolution images. The Super-resolution images were taken at 40x Airyscan SR mode (**a-g**, right three panels). Scale bar = SCG (10 µm), nerve bundles (2 µm). The experiments were repeated three times with similar results.

The endothelial cell cluster exhibited elevated expression of *Cav1*, *Vegfr2*, *Cdh5*, *Emcn*, *Cldn5* and *Egfl7* that were previously reported to be highly expressed in vascular endothelial cells^7–9^ (Fig. 1d). In contrast, we observed very low expression of lymphatic endothelial cell markers (*Lyve1*, *Prox1*) in the endothelial cell cluster^8,10^ (Extended Data Fig. 2a). Notably, we detected especially high expression of the leptin receptor (*Lepr*) gene in the endothelial cell population (average log_2_(fold change) (FC) = 1.96, percentage of endothelial cells expressing *Lepr* = 71%, percentage of all other cells expressing *Lepr* = 0.3%, *P* < 2.2 × 10^−16^; Fig. 1d,e). Quantification of *Lepr*^+^ endothelial cell population revealed that 50.6% of endothelial cells are *Lepr*^+^*Cav1*^+^ (Extended Data Fig. 2b,c), 37.8% of endothelial cells are *Lepr*^+^*Vegfr2*^+^ (Extended Data Fig. 2d,f) and 47.2% of endothelial cells are *Lepr*^+^*Cdh5*^+^ (Extended Data Fig. 2e,g). To visualize the sympathetic endothelial cells that expressed the leptin receptor, we performed immunofluorescence of SCG and sympathetic nerve bundles dissected from the subcutaneous white adipose tissue (scWAT) and BAT of *Lepr*^Cre^; *Rosa26*^−Lox-Stop-Lox-ChR2-YFP^ reporter (*Lepr*^Cre^; LSL-YFP) mice. In *Lepr*^Cre^; LSL-YFP mice, cre expression follows an IRES sequence downstream of the long form of the leptin receptor^11^, and the Cre-mediated recombination results in expression of membrane-bound YFP. Double staining of CAV1, VEGFR2 or CDH5 with YFP in the SCG and sympathetic nerve bundles revealed that YFP is co-expressed with endothelial cells positive for CAV1 (Extended Data Fig. 3a–c), VEGFR2 (Extended Data Fig. 3d,e) and CDH5 (Extended Data Fig. 3f,g) forming a thin layer surrounding the tyrosine hydroxylase-positive (TH^+^) sympathetic neurons.

### LEPR^+^ sympathetic endothelial cells constitute the perineurial barrier

**Fig. 2:**
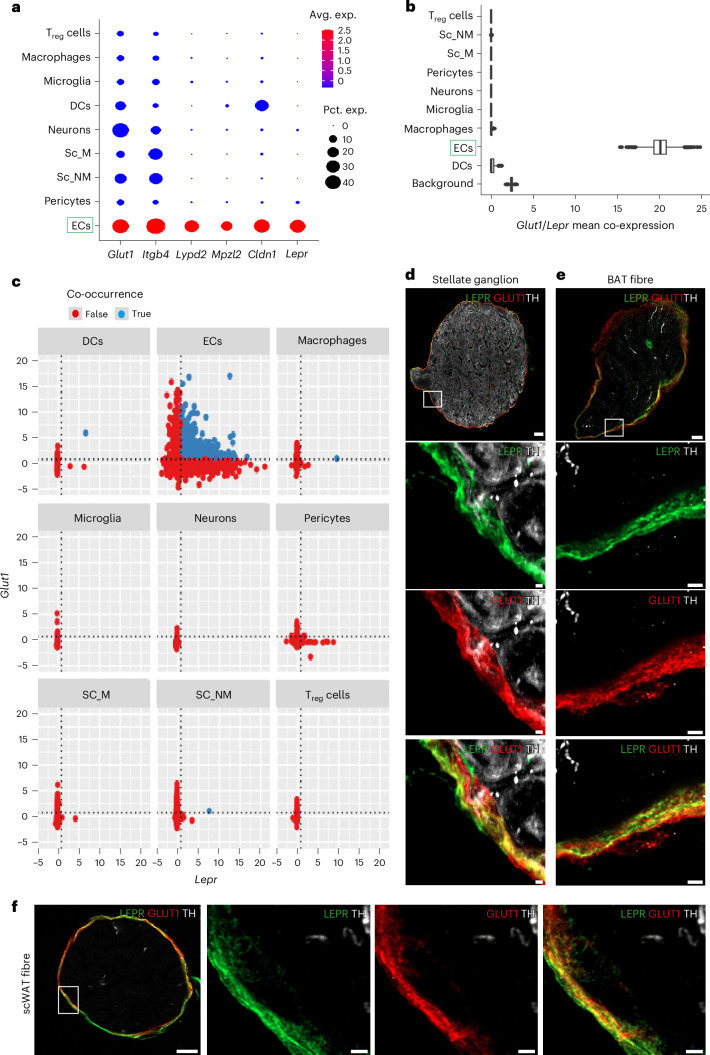
LEPR^+^ cells surround the sympathetic ganglia and sympathetic nerve bundles in adipose tissue as the perineurial barrier. **a**, Dot plot showing the expression of perineurial markers (*Glut1*, *Itgb4*, *Lypd2*, *Mpzl2*, *Cldn1*) and leptin receptor (*Lepr*) in the endothelial cell population. The size of the dot corresponds to the percentage of cells expressing the gene in each cluster, and the colour represents the average gene expression level. **b**, Box plot showing the percentage of cells co-expressing *Lepr* and *Glut1* across indicated cell types from scRNA-seq data of 20 lean mice (10 per experiment). Values were estimated by bootstrap resampling of pooled cells. Boxes indicate median and interquartile range of bootstrap distributions. **c**, Co-expression of *Lepr* and *Glut1* in different cell clusters observed in our scRNA-seq dataset. The position of the dotted lines is described in Methods. **d**–**f**, Immunofluorescence staining of LEPR (YFP), TH and GLUT1 protein in stellate ganglia (**d**) and sympathetic nerve bundles innervating the BAT (**e**) and scWAT (**f**) isolated from 12-week-old *Lepr*^Cre^; LSL-YFP reporter mice. Scale bars, 50 µm (stellate; left) and 10 µm (BAT and scWAT fibre; left). The white box indicates the field of view of the high-resolution images. The super-resolution images were taken at ×40 Airyscan SR mode. Scale bars, 10 µm (stellate; right) and 2 µm (nerve bundles; right). Source data

**Extended Data Fig. 4:**
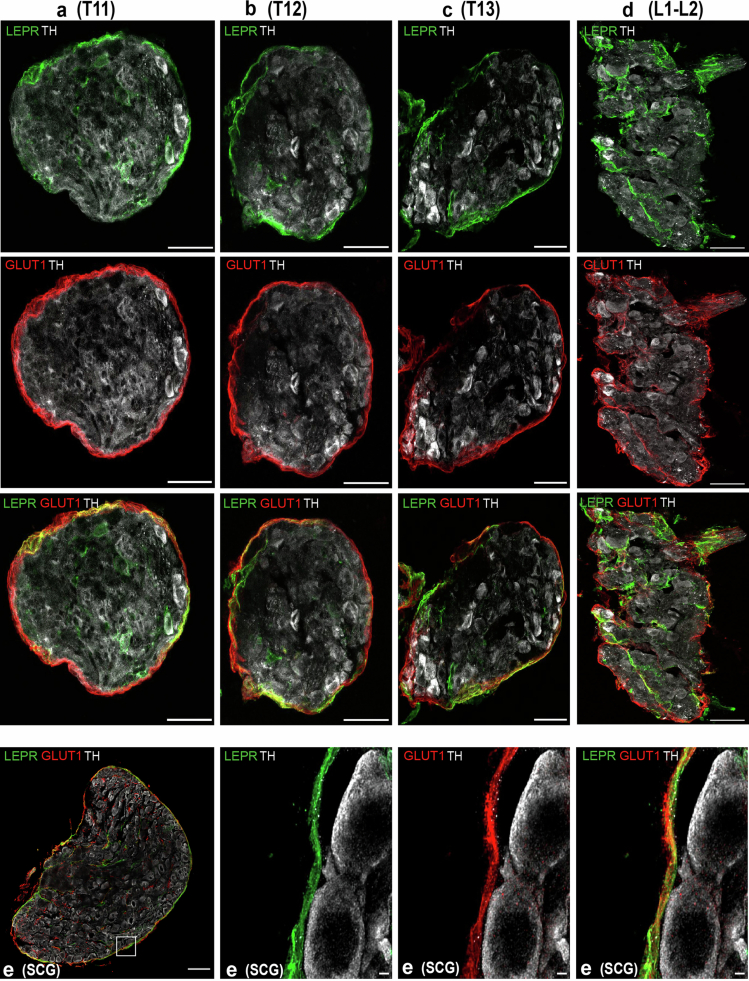
Immunofluorescence staining of LEPR (YFP), TH, and GLUT1 protein in T11 to L1-L2, SCG isolated from 12-week-old *Lepr*^Cre^; LSL-YFP reporter mice. **a-d**, The LEPR^+^ GLUT1^+^ perineurial barrier surrounding TH^+^ sympathetic neurons in T11 to L2 sympathetic ganglia that innervate WAT. Scale bar = 50 µm. **e**, LEPR (YFP) and GLUT1 expression in the perineurium of superior cervical ganglia (SCG). The white box indicates the field of view of the higher-resolution images. Scale bar = top panel (50 µm), lower panels (10 µm). The experiments were repeated three times with similar results.

The sheath-like structure formed by the LEPR^+^ endothelial cells resembles the perineurial barrier, which was once described as epithelial^12^. Interestingly, the scRNA-seq dataset reveals that the *Lepr*^+^ endothelial cell population also highly transcribes *Glut1*, *Itgb4*, *Lypd2*, *Mpzl2* and *Cldn1* (Fig. 2a), which have been reported as perineurial marker genes in the sciatic nerve single-cell atlas^8,13^. Among the perineurial markers, *Glut1*, which has been reported as a pan-perineurial marker^14^, was co-expressed predominantly in the *Lepr*^+^ endothelial cell cluster (Fig. 2c). Quantification of *Lepr*^+^*Glut1*^+^ cells among different clusters revealed around 20.2% of endothelial cells are *Lepr*^+^*Glut1*^+^ (Fig. 2b). In *Lepr*^Cre^; LSL-YFP reporter mice, immunolabelling for GLUT1, YFP and TH revealed the co-expression of LEPR and GLUT1 in the perineurium surrounding the TH^+^ sympathetic neurons of stellate ganglia (fused with T1) and T11–L2 sympathetic ganglia, which innervate BAT^15^ and scWAT^16^, respectively (Fig. 2d and Extended Data Fig. 4a–d). We also detected the co-expression of LEPR and GLUT1 in sympathetic nerve bundles innervating BAT (Fig. 2e), scWAT (Fig. 2f) and SCG (Extended Data Fig. 4e). We thus confirmed that the *Lepr*^+^ sympathetic endothelial cells constitute the perineurial barrier of the sympathetic ganglia and nerve bundles. We, therefore, refer to this endothelial cell population as SPCs.

### SPCs highly co-express *Lepr* and *Adrb2*, unlike any other cell type

**Fig. 3:**
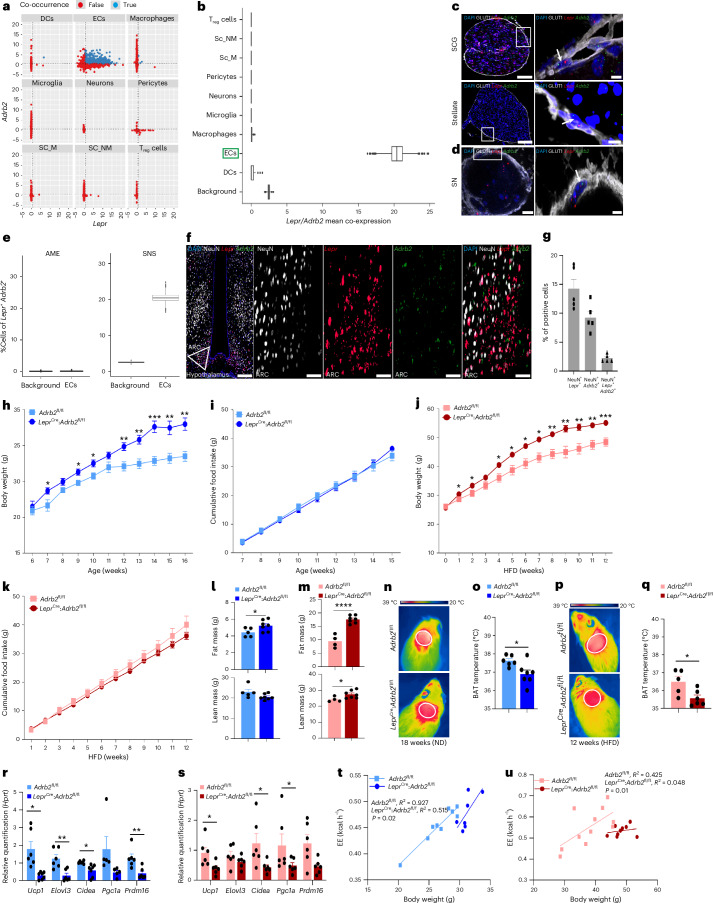
*Adrb2* in SPCs is required for metabolic homeostasis. **a**, scRNA-seq analysis shows co-expression of *Lepr* and *Adrb2* across cell populations; dotted line positions are described in Methods. **b**, Box plot showing the percentage of cells co-expressing *Lepr* and *Glut1* across indicated cell types from scRNA-seq data of 20 lean mice (10 per experiment). Values were estimated by bootstrap resampling of pooled cells. Boxes indicate the median and interquartile range of bootstrap distributions. **c**,**d**, High-magnification images showing dual labelling of GLUT1 protein and *Lepr*, *Adrb2* mRNA in SCG and stellate ganglia (**c**) and sympathetic nerve (SN) bundles (**d**) dissected from WT mice. Scale bars, 50 µm (**c**, left), 5 µm (**c**, right), 5 µm (**d**, left) and 2 µm (**d**, right). **e**, Box plots showing the percentage of cell co-expressing *Lepr* and *Adrb2* in the arcuate median eminence (ARC) and sympathetic ganglia. ARC data are derived from the HypoMap scRNA-seq dataset (single cells; cells are not independent biological replicates). Sympathetic nervous system (SNS) data are from our scRNA-seq analysis of sympathetic ganglia of 20 lean mice (10 per experiment); proportions were estimated per mouse using bootstrap resampling of cells. Boxes indicate the median and interquartile range of bootstrap distributions. **f**, High-magnification images showing dual labelling of NeuN protein and *Lepr*, *Adrb2* mRNA in the hypothalamus of WT mice. Scale bars, 100 µm (first image) and 20 µm (second to fifth images). **g**, Quantification of *Lepr*^+^*Adrb2*^+^ neurons in the ARC nucleus of the hypothalamus (*n* = 5 per group). **h**, Body weight of normal chow diet (ND)-fed *Lepr*^Cre^; *Adrb2*^fl/fl^ and *Adrb2*^fl/fl^ mice. *n* = (*Adrb2*^fl/fl^, 15), (*Lepr*^Cre^: *Adrb2*^fl/fl^, 19). **i**, Food intake of ND-fed *Lep*r^Cre^; *Adrb2*^fl/fl^ and *Adrb2*^fl/fl^ mice. *n* = (*Adrb2*^fl/fl^, 15), (*Lepr*^Cre^: *Adrb2*^fl/fl^, 19). *P* values by week: 6 (0.580), 7 (0.023), 8 (0.067), 9 (0.011), 10 (0.012), 11 (0.070), 12 (0.005), 13 (0.003), 14 (<0.001), 15 (0.004), 16 (0.001). **j**, Body weight of *Lepr*^Cre^; *Adrb2*^fl/fl^ and *Adrb2*^fl/fl^ mice challenged with a 12-week HFD. *n* = (*Adrb2*^fl/fl^, 16), (*Lepr*^Cre^; *Adrb2*^fl/fl^, 23). *P* values by week: 0 (0.580629), 1 (0.031367), 2 (0.023843), 3 (0.043335), 4 (0.005867), 5 (0.003517), 6 (0.001536), 7 (0.00082), 8 (0.000255), 9 (0.000762), 10 (0.000774), 11 (0.00062), 12 (0.000333). **k**, Food intake of *Lepr*^Cre^; *Adrb2*^fl/fl^ and *Adrb2*^fl/fl^ mice following HFD challenge. *n* = (*Adrb2*^fl/fl^, 16), (*Lepr*^Cre^; *Adrb2*^fl/fl^, 23). **l**, Body composition measured in 14-week-old ND-fed *Lepr*^Cre^; *Adrb2*^fl/fl^ and *Adrb2*^fl/fl^ mice. *n* = (*Adrb2*^fl/fl^, 5), (*Lepr*^Cre^; *Adrb2*^fl/fl^, 7). *P* (0.0481). **m**, Body composition measured in *Lepr*^Cre^; *Adrb2*^fl/fl^ and *Adrb2*^fl/fl^ mice following 10-week HFD challenge. *n* = (*Adrb2*^fl/fl^, 4), (*Lepr*^Cre^; *Adrb2*^fl/fl^, 7). *P* < 0.0001. **n**, Representative infrared thermal imaging of the interscapular BAT of ND-fed *Lepr*^Cre^; *Adrb2*^fl/fl^ and *Adrb2*^fl/fl^ mice. **o**, Quantification of BAT temperature. Each data point represents the average BAT temperature per mouse. *n* = (*Adrb2*^fl/fl^, 6), (*Lepr*^Cre^: *Adrb2*^fl/fl^, 7). *P* = 0.0194. **p**, Representative infrared thermal imaging of BAT in HFD-fed *Lepr*^Cre^; *Adrb2*^fl/fl^ and *Adrb2*^fl/fl^ mice. **q**, Quantification of BAT temperature. *n* = (*Adrb2*^fl/fl^, 5), (*Lepr*^Cre^; *Adrb2*^fl/fl^, 6). *P* = 0.025. **r**, Relative expression levels of thermogenic genes in BAT (ND), with individual data points representing biological replicates. *n* = (*Adrb2*^fl/fl^, 6), (*Lepr*^Cre^: *Adrb2*^fl/fl^, 7). *P* values: *Ucp1* (0.016), *Elovl3* (0.006), *Cidea* (0.011), *Pgc1α* (0.13), *Prdm16* (0.0098). **s**, Relative expression levels of thermogenic genes in BAT (HFD), with individual data points representing biological replicates. *n* = 6 per group. *P* values: *Ucp1* (0.035), *Elovl3* (0.212), *Cidea* (0.041), *Pgc1α* (0.142), *Prdm16* (0.139). **t**, Regression plots of energy expenditure (EE) as a function of body weight measured in age-matched (14–16-week-old) ND-fed *Lepr*^Cre^; *Adrb2*^fl/fl^ and *Adrb2*^fl/fl^ mice. *n* = (*Adrb2*^fl/fl^, 9) (*Lepr*^Cre^; *Adrb2*^fl/fl^, 7). **u**, Regression plots of EE as a function of body weight measured in 10-week-old HFD-fed *Lepr*^Cre^; *Adrb2*^fl/fl^ and *Adrb2*^fl/fl^ mice. *n* = (*Adrb2*^fl/fl^, 11), (*Lepr*^Cre^; *Adrb2*^fl/fl^, 9). Data are the mean ± s.e.m. and were analysed using two-way analysis of variance (ANOVA) with Bonferroni post hoc test (**h**–**k**), analysis of covariance (ANCOVA; **t** and **u**) and two-tailed unpaired Student’s *t*-test (**l**, **m**, **o** and **q**–**s**). *P < 0.05, **P < 0.01, ***P < 0.001, ****P < 0.0001. Source data

**Extended Data Fig. 5:**
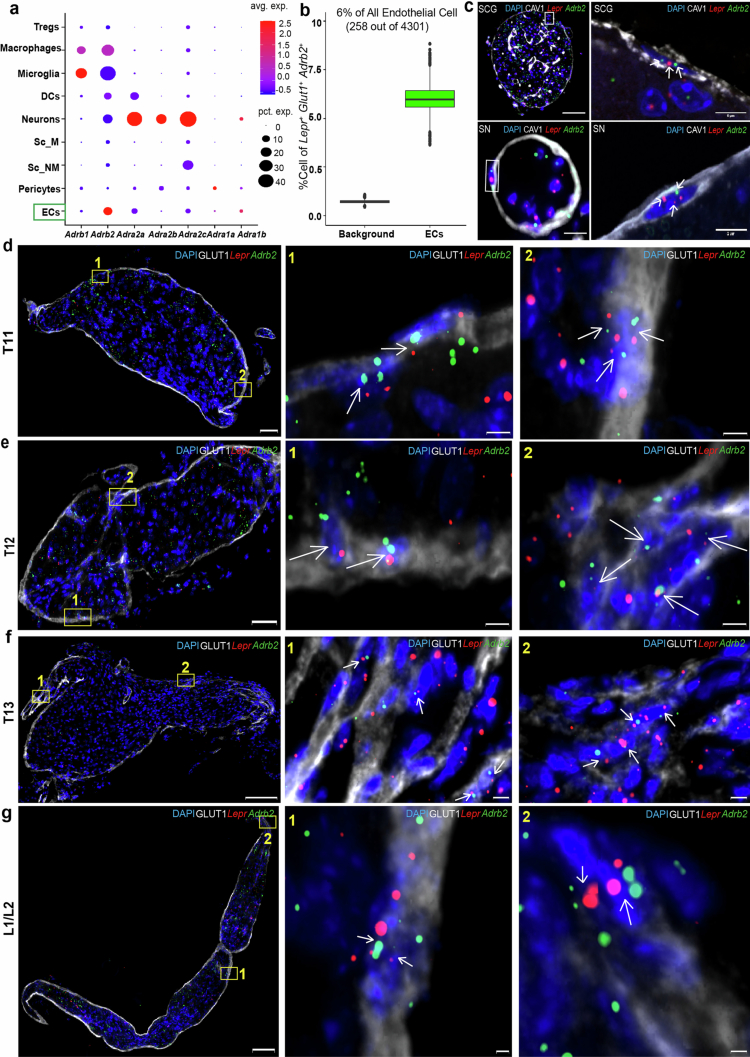
*Lepr*^+^ SPCs express β2 adrenergic (*Adrb2*) receptor in sympathetic ganglia innervating white and brown adipose tissue. **a**, Dot plot showing the expression of adrenergic receptors (*Adrb1*, *Adrb2*, *Adra2a*, *Adra2b*, *Adra2c*, *Adra1a*, and *Adra1b* in our scRNA sympathetic ganglia dataset. The size of the dot corresponds to the percentage of cells expressing the gene in each cluster, and the color represents the average gene expression level. **b**, Box plot shows the percentage of endothelial cells co-expressing *Lepr*, *Glut1*, and *Adrb2*from scRNA-seq data of 20 lean mice (10 per experiment). Values were estimated by bootstrap resampling of pooled cells. Boxes indicate median and interquartile range of bootstrap distributions. **c**, High magnification images showing the expression of CAV1 protein and *Lepr*, *Adrb2* mRNA in superior cervical ganglia (SCG) and sympathetic nerve (SN) bundles dissected from WT adult mice. **d-g**, The dual labelling of GLUT1 protein with IHC and *Lepr*, *Adrb2* mRNA with ISH in the T11 (**d**), T12 (**e**), T13 (**f**) and L1/L2 (**g**) of wild type mice. The yellow boxes in the left panel (40x) indicate the field of view of the higher resolution (120x) images in the right panel. Scale bar = left panel (50 µm), right panel (5 µm). ECs = endothelial cells, Sc_NM = Schwann cell_non myelinated, Sc_M = Schwann cell_myelinated, DCs = dendritic cells, Tregs = regulatory T cells. (**c-g**) The experiments were repeated twice with similar results. Source data

We next questioned whether SPCs could sense the sympathetic efferent arm in the neuroendocrine loop of leptin. To answer this question, we first checked in the scRNA-seq dataset whether SPCs express any adrenergic receptors^17^. Among different adrenergic receptors, *Adrb2* is highly expressed in the endothelial cell cluster. In contrast, we found very low expression of alpha-adrenergic receptors and no expression of β_3_ adrenergic receptor in the endothelial population (Extended Data Fig. 5a). Co-expression analysis further showed that *Lepr* and *Adrb2* are predominantly co-expressed in 20.4% of the endothelial cell population (Fig. 3a,b), and 6% of all endothelial cells are *Lepr*^+^*Glut1*^+^*Adrb2*^+^ (Extended Data Fig. 5b). To validate the scRNA-seq results, we performed fluorescence in situ hybridization (ISH) in combination with GLUT1 or CAV1 immunohistochemistry by RNAscope to detect the *Lepr* and *Adrb2* mRNA in the SPC barrier. We observed co-expression of *Lepr* and *Adrb2* in the GLUT1^+^ (Fig. 3c,d) and CAV1^+^ (Extended Data Fig. 5c) SPC barrier in stellate, SCG and sympathetic nerve bundles. We also confirmed the co-expression of *Lepr* and *Adrb2* in the GLUT1^+^ perineurial barriers of T11–L2 ganglia, which innervate scWAT (Extended Data Fig. 5d–g).

**Extended Data Fig. 6:**
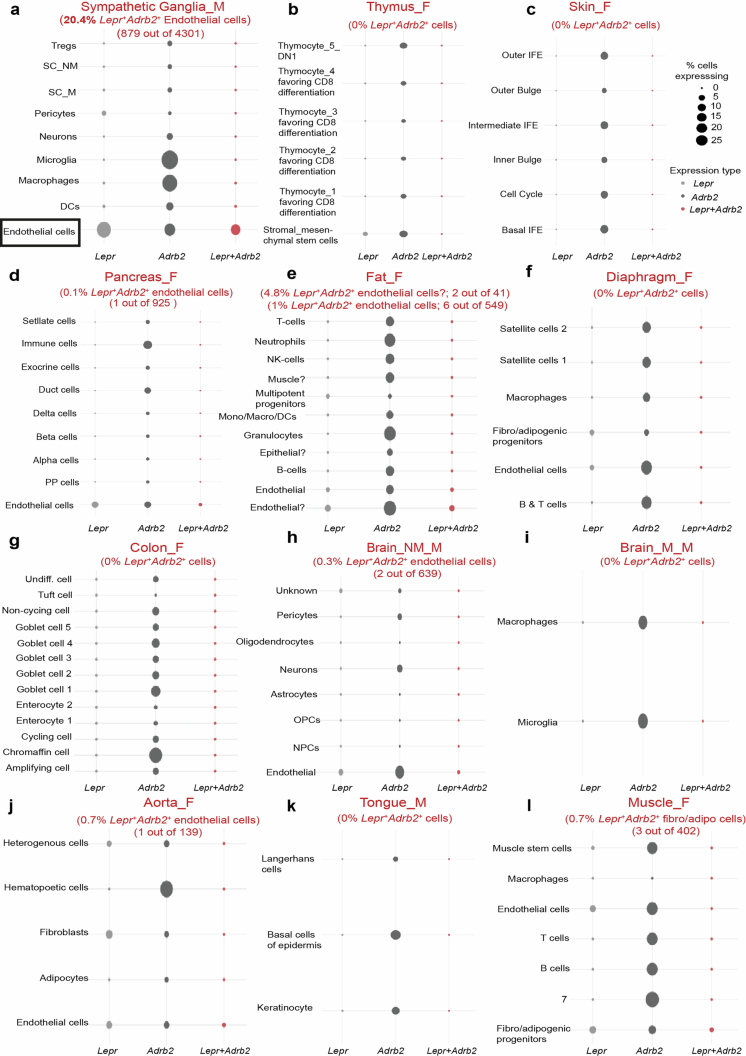
Detailed analysis of Tabula Muris Consortium showing no notable co-expression of *Lepr* and *Adrb2* across different cell types in thymus, skin, pancreas, fat, diaphragm, colon, brain, aorta, tongue, and muscle, whereas only endothelial cells in our sympathetic ganglia dataset exhibit substantial co-expression. **a**, Dot plot showing the percentage of *Lepr*^+^ *Adrb2*^+^ cells in our sympathetic ganglia dataset. **b-l**, Dot plot showing the percentage of *Lepr*^+^ *Adrb2*^+^ cells in different mouse organs documented in Tabula Muris Consortium. M = Microfluidic droplet method. F = FACS-based method. Cells with normalized expression values > 0.75 for both genes were considered positive for co-expression. Source data

**Extended Data Fig. 7:**
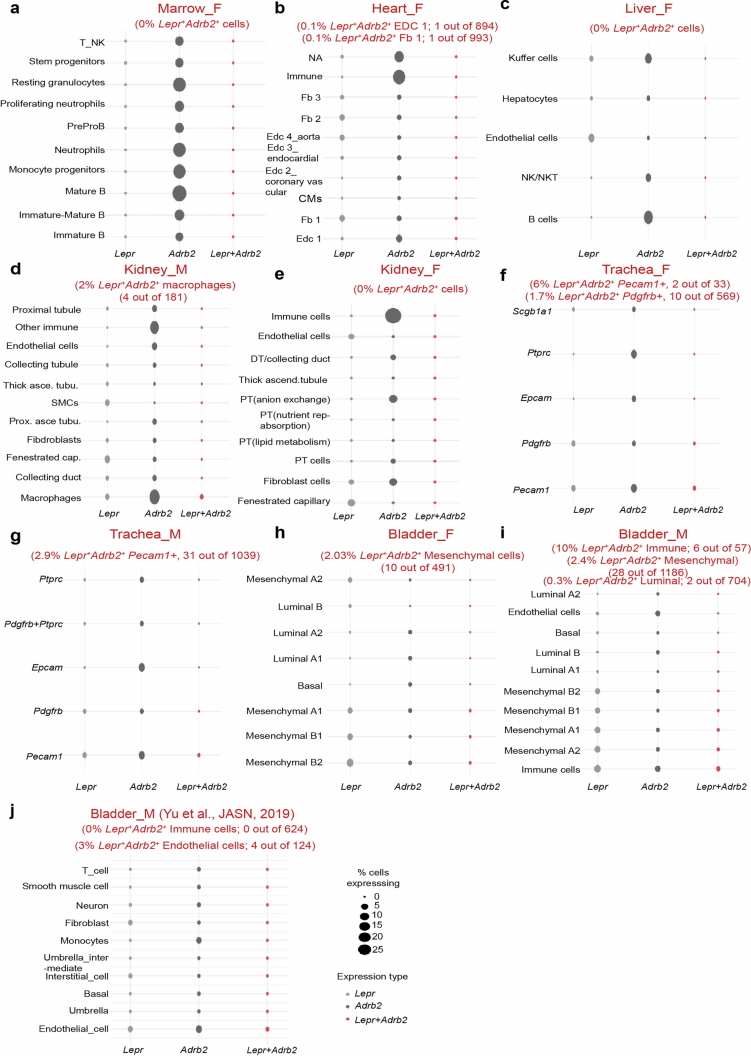
Detailed analysis of Tabula Muris Consortium showing no notable co-expression of *Lepr* and *Adrb2* across different cell types in marrow, heart, liver, kidney, trachea, and bladder. **a-i**, Dot plot showing the percentage of *Lepr*^+^ *Adrb2*^+^ cells in different mouse organs documented in Tabula Muris Consortium. **j**, Dot plot of the percentage of *Lepr*^+^*Adrb2*^+^ cells across different cell types in mouse bladder (Yu et al., JASN 2019; PMID: 31462402). M = Microfluidic droplet method. F = FACS-based method. Cells with normalized expression values > 0.75 for both genes were considered positive for co-expression. Source data

**Extended Data Fig. 8:**
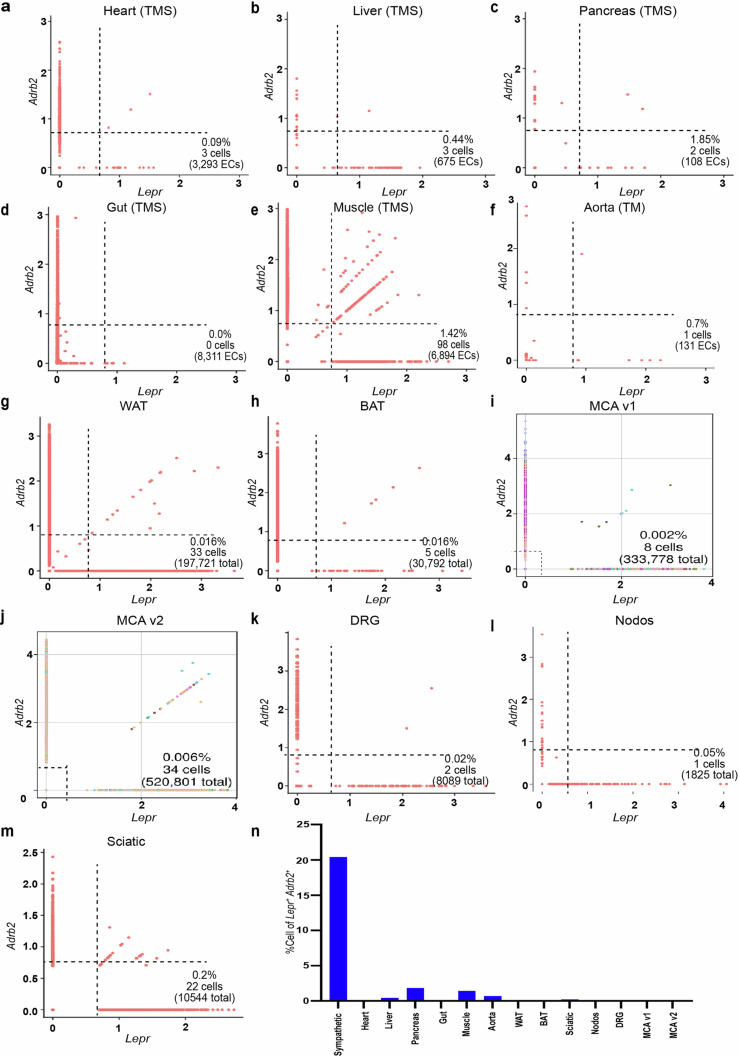
*Lepr*^+^ SPCs express β2 adrenergic (*Adrb2*) receptor, and other cells in other organs show little or no *Lepr* and *Adrb2* co-expression. **a-m**, Correlogram showing the expression of Lepr and Adrb2 across different scRNA datasets and tissues. The position of the dotted lines is described in the Methods. **n**, Bar plot representing the percentage of double-positive Lepr^+^Adrb2^+^ endothelial cells across tissues and datasets. ECs = endothelial cells, MCA = Mouse Cell Atlas, versions v1 and v2, TMS = Tabula Muris Senis, TM = Tabula Muris, WAT = white adipose tissue, BAT = brown adipose tissue, DRG = dorsal root ganglia. Source data

**Extended Data Fig. 9:**
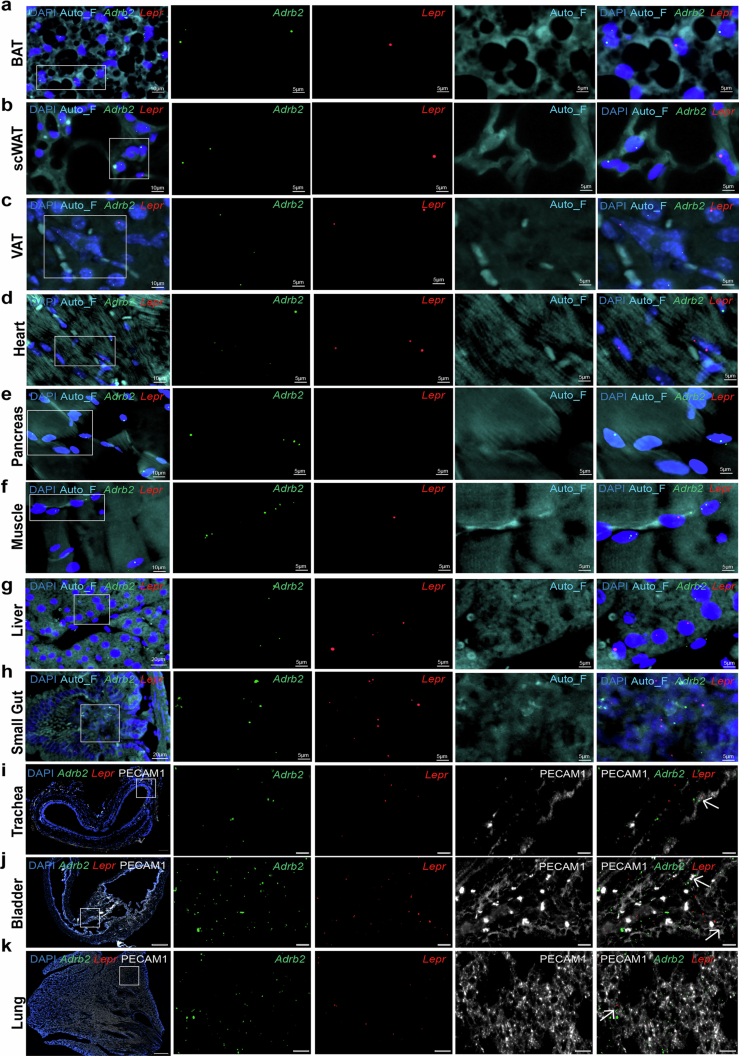
Peripheral organs show low or no co-expression of *Lepr* and *Adrb2*. High magnification images showing the expression of *Lepr* (red) and *Adrb2* (green) mRNA in (**a)** brown adipose tissue (BAT), (**b)** subcutaneous white adipose tissue (scWAT), (**c)** visceral adipose tissue (VAT), (**d)** heart, (**e)** pancreas, (**f)** skeletal muscle, (**g)** liver and (**h)** small gut of adult wild type mice. Background autofluorescence (Auto_F) was brightened to visualize the tissue architecture (cyan). The boxed areas in the left panels (60x) are shown at higher magnification (120x) on the right. (**i-k**) High magnification image showing the expression of *Lepr* mRNA (red) and *Adrb2* mRNA (green) in PECAM1+ (white) endothelial cells in trachea (**i**), bladder (**j**), and lung (**k**). Cell nuclei were labelled with DAPI (blue). The boxed areas in the left panels (4x) are shown at higher magnification (40x) at right. Scale bar = left panel (100 µm), right panel (20 µm). The experiments were repeated twice with similar results.

Next, we investigated whether *Lepr* and *Adrb2* were co-expressed in any other tissues. To this end, we analysed the co-expression of *Lepr* and *Adrb2* within individual annotated cell types for each organ represented in the Tabula Muris dataset^18^. No double-positive cells were detected in thymus, skin, diaphragm, colon, brain microglia, tongue, bone marrow, liver or kidney (FACS-based datasets). In pancreas (0.1%, 1/925 endothelial cells), fat (1%, 6/549 endothelial cells), brain non-microglia (0.3%, 2/639 endothelial cells), aorta (0.7%, 1/139 endothelial cells), muscle (0.7%, 3/402 endothelial cells), heart (0.1%, 1/894 endothelial cells or 1/993 fibroblasts), kidney (2%, 4/181 cells in droplet-based sequencing) and trachea (2.9%, 31/1,039), double-positive cells were exceptionally rare (Extended Data Figs. 6 and 7a–g. In bladder, droplet-based datasets detected low frequencies of double-positive cells in mesenchymal (2.4%, 28/1,186) and immune (10%, 6/57) populations, whereas FACS-based analysis identified only 2.0% (10/491) double-positive mesenchymal cells (Extended Data Fig. 7h,i). Analysis of an independent bladder-specific scRNA-seq dataset^19^ revealed no double-positive immune cells and only 3% (4/124) double-positive endothelial cells (Extended Data Fig. 7j). We further analysed the co-expression of this gene pair in the endothelial cell clusters extracted from mouse heart, liver, pancreas, gut and muscle^18,20^. We observed that in specific organs such as the heart (0.09%, 3 cells of 3,293), liver (0.44%, 3 of 675), pancreas (1.85%, 2 cells of 108), gut (0%, 0 cell out to 8,311) and muscle (1.42%, 98 cells of 6,894), double-positive cells were scarce (Extended Data Fig. 8a–e). We extended our analysis to fat tissue, using single-nucleus RNA-seq data from WAT^21^ (0.016%, 33 cells of 197,721) and BAT^22^ (0.016%, 5 cells of 30,792; Extended Data Fig. 8g,h). In both cases, double-positive cells were statistically indistinguishable from sequencing noise. To further validate our model, we performed RNAscope dual ISH staining in these tissues (heart, liver, fat, pancreas, muscle, gut, trachea, bladder and lung), confirming the negligible presence of *Lepr*^+^*Adrb2*^+^ cells in these organs (Extended Data Fig. 9). We expanded our co-expression analysis to additional large cohorts of multiple tissues: the Mouse Cell Atlas (MCA, versions 1 and 2)^23,24^. In the MCA v1, we found that only 8 cells co-expressed *Lepr* and *Adrb2*, representing 0.002% of the 333,778 cells, which was not distinguishable from noise. In the MCA v2, which comprises developing, adult and aged mice, we found only 34 cells co-expressing these genes, representing 0.006% of the 520,801 cells, which was not distinguishable from noise (Extended Data Fig. 8i,j). Given the potential presence of the perineurial cells in sensory/parasympathetic ganglia or the sciatic nerve, we also analysed *Lepr Adrb2* co-expression in three additional single-cell datasets, including data from the dorsal root ganglia^25^, nodose ganglia^26^ and the sciatic nerve^13^. Co-expression of *Lepr* and *Adrb2* was rare in the dorsal root ganglia (2/8,089; 0.02%) and nodose ganglia (1/1,825; 0.05%), and frequencies were indistinguishable from noise (Extended Data Fig. 8k,l). In the sciatic nerve, 22 of 10,554 cells (0.2%) expressed both markers, a marginal increase likely reflecting the known 25% sympathetic axon in rats (Extended Data Fig. 8m). By contrast, sympathetic ganglia co-expressed *Lepr* and *Adrb2* in 1,761/73,042 (2.4% of all cells), representing 20.4% of endothelial cells (Extended Data Fig. 8n). Our analysis did not find substantial co-expression of *Lepr* and *Adrb2* in any other cell type in other organs thus far.

**Extended Data Fig. 10:**
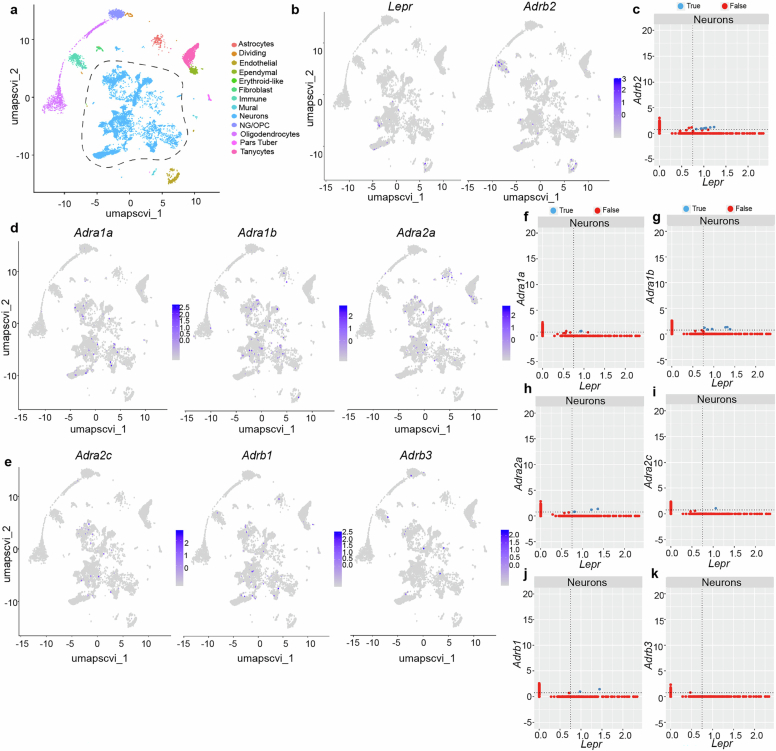
Leptin receptor and different adrenergic receptors co-expression analysis in arcuate hypothalamic scRNA dataset extracted from HypoMap. **a**, UMAP plot to show the major cell populations in the arcuate hypothalamus. **b**, UMAP plot to show the expression of *Lepr* and *Adrb2* in different cell clusters of the arcuate hypothalamus. **c**, Correlogram shows the co-expression of *Lepr* and *Adrb2* in the neuronal population of the arcuate hypothalamus. **d-e**, UMAP plot to show the expression of other adrenergic receptors (*Adra1a*, *Adra1b*, *Adra2a*, *Adra2c*, *Adrb1*, and *Adrb3*) in different cell clusters of the arcuate hypothalamus. **f-k**, Correlogram to show the co-expression of *Lepr* and other adrenergic receptors in the neuronal population of the arcuate hypothalamus. The position of the dotted lines is described in the Methods. Source data

Neurons in the arcuate nucleus of hypothalamus express leptin and adrenergic receptors, which play a major role in regulating food intake as well as sympathetic outflow to white and brown adipose tissues^27–30^. We, therefore, analysed the HypoMap scRNA-seq dataset to investigate the possible presence of *Lepr*^+^ *Adrb2*^+^ co-expression in the arcuate neuronal populations^31^. Our analysis revealed a negligible percentage of neurons (0.01%) in the arcuate hypothalamus co-express *Lepr* and *Adrb2* mRNA compared with that of sympathetic endothelial cells (20.4%; Fig. 3e and Extended Data Fig. 10a–c). This observation was further validated by dual labelling of *Lepr* and *Adrb2* mRNA in NeuN-positive neurons in the arcuate hypothalamus by fluorescence ISH and immunohistochemistry (Fig. 3f). The quantification of *Lepr*^+^*Adrb2*^+^ neurons in arcuate hypothalamic region revealed an average of only 2.3% ± 0.3% central nervous system neurons as *Lepr*^+^*Adrb2*^+^ (Fig. 3g). We also analysed the arcuate neuronal populations within the HypoMap dataset to ascertain whether *Lepr* is co-expressed with other alpha-adrenergic and beta-adrenergic receptors. Our co-expression analysis excluded the presence of any notable co-expression of *Lepr* with any other adrenergic receptor subtypes in arcuate neurons (Extended Data Fig. 10d–k).

### *Lepr*^Cre^:*Adrb2*^fl/fl^ mice have lower energy expenditure and thermogenesis, and higher obesity risk, independently of food intake

To test whether *Adrb2* in SPCs was functionally relevant to body weight homeostasis, we generated *Lepr*^Cre^; *Adrb2*^fl/fl^ conditional knockout mice, resulting in the deletion of *Adrb2* in *Lepr*^+^*Adrb2*^+^ cells, predominantly in SPCs. Immunolabelling with GLUT1 protein and *Lepr*, and *Adrb2* mRNA of stellate ganglia confirmed the absence of *Adrb2* in the *Lepr*^+^ SPCs of *Lepr*^Cre^; *Adrb2*^fl/fl^ mice (Supplementary Fig. 1a,b). Next, we characterized the metabolic phenotype of *Lepr*^Cre^; *Adrb2*^fl/fl^ and control *Adrb2*^fl/fl^ mice under chow-fed and HFD-fed conditions. We discovered that *Lepr*^Cre^; *Adrb2*^fl/fl^ mice kept on a chow diet displayed a consistent pattern of significant weight gain compared with *Adrb2*^fl/fl^ mice, beginning at 7 weeks of age. This difference was more pronounced and highly significant from the age of 12 weeks to 16 weeks (Fig. 3h) and occurred independently of food intake (Fig. 3i). The weight gain in *Lepr*^Cre^; *Adrb2*^fl/fl^ mice was associated with increased adiposity, in particular higher BAT and visceral adipose tissue (VAT) mass without changes in lean mass and key skeletal muscles (Fig. 3l and Supplementary Fig. 1c). Indirect calorimetry revealed that *Lepr*^Cre^; *Adrb2*^fl/fl^ mice have lower daily energy expenditure (Fig. 3t) and a trend towards reduced O_2_ consumption (VO_2_) and CO_2_ production (VCO_2_; Supplementary Fig. 1d,e) with no changes in respiratory exchange ratio (RER) and total activity (Supplementary Fig. 1f,g and Supplementary Table 1a). Consistent with this observation, *Lepr*^Cre^; *Adrb2*^fl/fl^ have lower BAT temperature (Fig. 3n,o) and decreased expression of thermogenic genes *Ucp1*, *Elovl3*, *Cidea* and *Prdm16* (Fig. 3r). When fed a HFD, *Lepr*^Cre^; *Adrb2*^fl/fl^ mice gained weight faster and were heavier than controls (Fig. 3j), despite no difference in food intake (Fig. 3k). Following the HFD challenge, *Lepr*^Cre^: *Adrb2*^fl/fl^ developed pronounced adiposity compared with control *Adrb2*^fl/fl^ mice. While some increase in lean mass was observed in *Lepr*^Cre^: *Adrb2*^fl/fl^ mice (~13% above control levels), the increase in fat mass was substantially more significant, reaching ~85% higher than that of HFD-fed *Adrb2*^fl/fl^ mice (Fig. 3m). As observed in the chow-fed state, BAT and VAT depots were significantly larger in HFD-fed *Lepr*^Cre^: *Adrb2*^fl/fl^ mice, without any changes in skeletal muscle mass (Supplementary Fig. 1i). In addition, HFD-treated *Lepr*^Cre^; *Adrb2*^fl/fl^ mice have lower energy expenditure (Fig. 3u), VO_2_ (Supplementary Fig. 1j) and VCO_2_ (Supplementary Fig. 1k) with no changes in RER and locomotor activity (Supplementary Fig. 1l,m and Supplementary Table 1b). Similar to the chow-fed mice, HFD-treated *Lepr*^Cre^; *Adrb2*^fl/fl^ mice showed lower BAT activity (Fig. 3p,q) and a significant reduction in thermogenic gene expression (*Ucp1*, *Cidea* and *Pgc1α*; Fig. 3s).

To exclude the possibility that Cre itself may interfere with Lepr production, we also characterized the metabolic phenotype of *Lepr*^Cre^ mice and *Lepr*^Cre^: *Adrb2*^fl/fl^ mice in both the chow-fed state and the 12-week-HFD-challenged state. We observed that *Lepr*^Cre^: *Adrb2*^fl/fl^ mice gained significantly more weight compared with *Lepr*^Cre^ mice in both chow-fed (Supplementary Fig. 2a) and HFD-challenged (Supplementary Fig. 2m) states, independent of food intake (Supplementary Fig. 2b,n). Compared with *Lepr*^Cre^ mice, *Lepr*^Cre^: *Adrb2*^fl/fl^ mice had reduced energy expenditure, VO_2_ and VCO_2_ in both chow-fed (Supplementary Fig. 2e–g) and HFD-fed (Supplementary Fig. 2q–s) conditions, with no changes in physical activity (Supplementary Fig. 2i,u). Importantly, *Lepr*^Cre^: *Adrb2*^fl/fl^ mice had significantly lower BAT temperature (Supplementary Fig. 2j,k,v,w) and reduced expression of thermogenic genes (Supplementary Fig. 2l,x) compared with *Lepr*^Cre^ mice in both dietary conditions. These data suggest that the obesogenic phenotype in *Lepr*^cre^: *Adrb2*^fl/fl^ mice was not due to Lepr-driven Cre expression. Altogether, we demonstrate that the expression of *Adrb2* in *Lepr*^+^ SPCs is essential for sustaining energy expenditure and thermogenesis and for protecting against obesity.

### Diet-induced-obesity-driven hyperleptinaemia destroys the SPC barrier

**Fig. 4:**
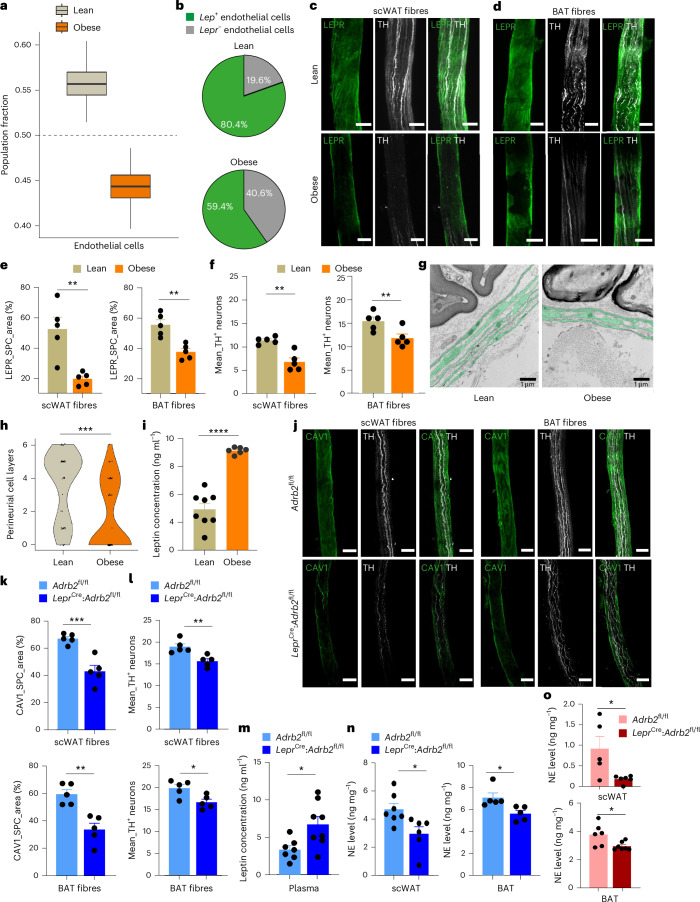
Obesity-driven hyperleptinaemia reduces SPCs and destroys the perineurial barrier. **a**, scRNA-seq of ganglia showing the fraction of endothelial cell population in chow-fed lean and HFD-induced obese WT mice. Fractions were estimated per mouse from scRNA-seq data using bootstrap resampling of cells (lean *n* = 20; obese *n* = 10). Boxes indicate median and interquartile range of bootstrap distributions. **b**, Pie chart shows the percentage of *Lepr*^+^ endothelial cells in lean and obese endothelial populations within ganglia. **c**,**d**, Representative images of scWAT (**c**) and BAT (**d**) nerve bundles from chow-fed lean and HFD-fed obese (*Lepr*^cre^; LSL-YFP) reporter mice showing the expression of LEPR (YFP) and TH. Scale bars, 50 µm. **e**, Quantification of LEPR^+^ SPC barrier in scWAT (*P* = 0.003) and BAT (*P* = 0.002) fibres of lean and DIO mice (*n* = 5 mice per group). **f**, Quantification of TH^+^ sympathetic neurons in scWAT (*P* = 0.001) and BAT (*P* = 0.002) fibres of lean and DIO mice (*n* = 5 mice per group). **g**, Electron micrograph showing the perineurial barrier in scWAT bundles of lean and obese mice with false colouring to highlight the perineurial layers (green). **h**, Quantification of the perineurial layer in obese and lean scWAT bundles (*n* = 3 mice per group); *P* = 3.63 × 10^−^^7^. **i**, Plasma leptin level in lean and DIO mice (*n* = 7 or 8 mice per group). (Lean *n* = 8; obese *n* = 6); *P* < 0.0001. **j**, Representative images of scWAT and BAT nerve fibres from chow-fed 10-week-old *Lepr*^Cre^; *Adrb2*^fl/fl^ and *Adrb2*^fl/fl^ mice showing the expression of CAV1 and TH. Scale bars, 50 µm. **k**, Quantification of CAV1^+^ SPC barrier in obese *Lepr*^Cre^; *Adrb2*^fl/fl^ and lean *Adrb2*^fl/fl^ scWAT (*P* = 0.0009) and BAT (*P* = 0.002) fibres (*n* = 5 mice per group). **l**, Quantification of TH^+^ sympathetic neurons in chow-fed *Lepr*^Cre^; *Adrb2*^fl/fl^ and *Adrb2*^fl/fl^ scWAT (*P* = 0.006) and BAT (*P* = 0.016) bundles (*n* = 5 mice per group). **m**, Plasma leptin level in 10-week-old chow-fed *Lepr*^Cre^; *Adrb2*^fl/fl^ (*n* = 8) and *Adrb2*^fl/fl^ (*n* = 7) mice; *P* = 0.017. **n**, Noradrenaline (NE) content in scWAT (*n* = 6 (*Lepr*^Cre^; *Adrb2*^fl/fl^) and *n* = 7 (*Adrb2*^fl/fl^); *P* = 0.019) and BAT (*n* = 5 per group; *P* = 0.023) fat pads isolated from 10-week-old chow-fed *Lepr*^Cre^; *Adrb2*^fl/fl^ and *Adrb2*^fl/fl^ mice (*n* = 5 or 6 mice per group). **o**, NE content in scWAT (*n* = 5 per mice group; *P* = 0.0241) and BAT (*n* = 6 mice per group; *P* = 0.021) fat pads isolated from 10-week-old HFD-challenged *Lepr*^Cre^; *Adrb2*^fl/fl^ and *Adrb2*^fl/fl^ mice. Data were analysed by two-tailed unpaired Student’s *t*-test and are shown as the mean ± s.e.m. *P < 0.05, **P < 0.01, ***P < 0.001, ****P < 0.0001. Source data

We next asked whether obesity changes the integrity of the SPC barrier. Analysis of the frequency of cells in the endothelial cluster of lean and obese sympathetic ganglia revealed a decreased proportion of endothelial cells in obese mice (Fig. 4a). Comparison analysis of the *Lepr*^+^ endothelial subset between lean and obese states further depicted a 20% reduction of *Lepr*^+^ endothelial cells in the sympathetic ganglia of obese mice (Fig. 4b). To corroborate the scRNA-seq analysis, we immunolabelled the scWAT and BAT sympathetic nerve bundles of 12-week-old HFD-induced obese (DIO) and chow-fed lean *Lepr*^Cre^; LSL-YFP reporter mice (Fig. 4c,d and Supplementary Fig. 3). These studies revealed a significant reduction in LEPR^+^ SPC area in the sympathetic nerve bundles innervating scWAT and BAT of obese mice compared with aged-matched lean mice (Fig. 4e). The density of TH^+^ sympathetic axons was also significantly decreased in scWAT and BAT sympathetic nerve bundles of obese mice (Fig. 4f). By using electron microscopy on scWAT-derived sympathetic nerve bundles of DIO and chow-fed lean mice, we confirmed a significant loss of perineurial cell layers in obese mice compared with their lean littermates (Fig. 4g,h). We also immunolabelled the scWAT and BAT sympathetic nerve fibres of 10-week-old chow-fed *Lepr*^Cre^; *Adrb2*^fl/fl^ and *Adrb2*^fl/fl^ mice. Similar to DIO mice, we observed a significant reduction in CAV1^+^ SPC area in the sympathetic nerve bundles innervating scWAT and BAT of obese *Lepr*^Cre^; *Adrb2*^fl/fl^ mice compared with age-matched lean *Adrb2*^fl/fl^ mice (Fig. 4j,k). The density of TH^+^ sympathetic axons was also significantly lower in scWAT and BAT sympathetic nerve bundles of *Lepr*^Cre^; *Adrb2*^fl/fl^ mice (Fig. 4j,l). Notably, we observed a significantly higher leptin level in *Lepr*^Cre^; *Adrb2*^fl/fl^ mice (Fig. 4m), similar to DIO mice (Fig. 4i). In addition, *Lepr*^Cre^; *Adrb2*^fl/fl^ mice had lower noradrenaline levels in scWAT and BAT in both chow-fed (Fig. 4n) and HFD-treated (Fig. 4o) conditions compared with *Adrb2*^fl/fl^ mice. Together, our data suggest that DIO disrupts the SPC barrier, which is concomitant with sympathetic neuropathy^32,33^ and reduced sympathetic activity in adipose tissues. By examining leptin-deficient *ob*/*ob* mice, we also confirmed that the SPC barrier and the density of TH^+^ neurons remain unaltered between *ob*/*ob* mice and their lean wild-type (WT) littermates (Supplementary Fig. 4). This indicates that SPCs are not lost in a hypoleptinaemic state, and provides additional support for our model, which posits that perineurial cells die in response to hyperleptinaemia, and not just obesity.

### Obesity and high leptin drive apoptosis of SPCs, which is prevented by sympathomimetic ADRB2 agonism

**Fig. 5:**
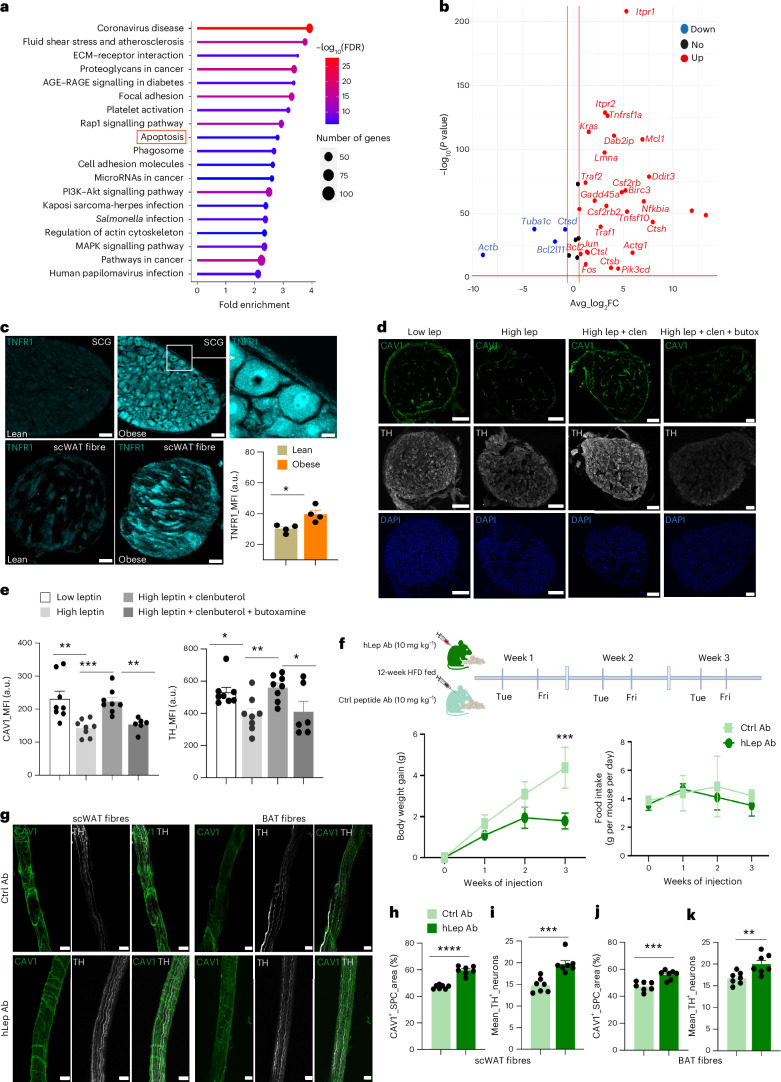
Obesity-induced hyperleptinaemia drives apoptosis of the SPCs, which is reversed by β_2_-adrenergic agonism. **a**, The top 20 enriched KEGG pathways using the DEGs between lean and obese sympathetic endothelial cell populations. **b**, Volcano plot showing the DEGs between lean and obese sympathetic endothelial cell populations involved in apoptosis. The logarithms of the fold changes of individual genes (*x* axis) are plotted against the negative logarithm of their *P* value to base 10 (*y* axis). Positive log_2_(fold change) values represent upregulation (red) and negative values represent downregulation (blue) in obese mice. The red vertical lines represent more than 50% of the fold change. **c**, TNFR1 expression in SCG and scWAT bundles isolated from lean and diet-induced obese mice. Scale bars, 50 µm. (*n* = 4 mice per group); (*P* = 0.016). **d**, Representative images of SCG explant culture showing the expression of CAV1, TH and DAPI following low leptin (10 ng ml^−1^), high leptin (100 ng ml^−1^), high leptin (100 ng ml^−1^) + clenbuterol (10 µg ml^−1^), high leptin (100 ng ml^−1^) + clenbuterol (10 µg ml^−1^) + butoxamine (10 µM) treatment. Scale bars, 100 µm. **e**, Quantification of CAV1 and TH in SCG explants following different stimulating conditions (*n* = 8, low leptin; 8, high leptin; 8, high leptin + clenbuterol; 6 mice, high leptin + clenbuterol + butoxamine). CAV1_MFI, *P* = 0.003 (low lep versus high lep), 0.0002 (high lep versus high lep + clen), 0.001 (high lep + clen versus high lep + clen + butox). TH_MFI, *P* = 0.01 (low lep versus high lep), 0.003 (high lep versus high lep + clen), 0.04 (high lep + clen versus high lep + clen + butox). **f**, Scheme of hLep Ab or Ctrl Ab treatment in DIO mice. Body weight gain (*P* = 0.0006, week 3) and food intake were measured during antibody treatment (*n* = 7 per group). **g**, Representative images of scWAT and BAT bundles from obese mice treated with hLep Ab or Ctrl Ab showing the expression of CAV1 and TH. Scale bars, 50 µm. **h**–**k**, Quantification of CAV1^+^ SPC barrier and TH^+^ sympathetic neurons in scWAT (SPCs, *P* < 0.0001; TH, *P* = 0.0004) and BAT (SPCs, *P* = 0.0005; TH, *P* = 0.007) fibres from hLep Ab- and Ctrl Ab-treated mice (*n* = 7 mice per group). a.u., arbitrary units; butox, butoxamine; clen, clenbuterol; lep, leptin; MFI, mean fluorescence intensity. Data are the mean ± s.e.m. and were analysed using two-tailed unpaired Student’s *t*-test (**c** and **h**–**k**) and one-way ANOVA with Turkey’s multiple-comparison test (**e** and **f**). *P < 0.05, **P < 0.01, ***P < 0.001. Schematic in **f** created in BioRender; Sarker, G. https://biorender.com/aw7m9xh (2026). Source data

We next asked how DIO causes loss of the perineurial barrier. To answer that question, we performed functional enrichment analysis of the differentially expressed genes (DEGs; adjusted *P* value < 0.05) in endothelial cell clusters between obese and lean mice based on Kyoto Encyclopedia of Genes and Genomes (KEGG) pathways using ShinyGO 0.77 (with a false discovery rate cut-off of 0.05)^34^. The top 20 enriched pathways are represented in the dot plot (Fig. 5a). Notably, KEGG pathway analysis revealed the enrichment of apoptotic process in obese endothelial cluster compared with the lean mice (Fig. 5a). Differential gene expression analysis of endothelial cells show that the genes listed in the cellular apoptotic pathways were mostly upregulated in obese mice (Fig. 5b). Hence, we next immunolabelled the SCG and scWAT-derived sympathetic nerve bundles of 12-week-old DIO obese and lean mice for one of the apoptotic markers, *Tnfrsf1a* (also known as TNFR1). Consistent with the scRNA-seq analysis, we observed a higher expression of TNFR1 in the SPC barrier and the sympathetic neurons of SCG and scWAT sympathetic nerve bundles (Fig. 5c) of obese mice compared with their lean littermates. The presence of TUNEL-immunoreactive cells in the SCG of DIO mice further confirms the occurrence of apoptosis of SPCs and sympathetic neurons in obese mice (Supplementary Fig. 5a). Together, these data suggests that diet-induced hyperleptinaemic obesity drives apoptosis of the SPC barrier and sympathetic neurons, resulting in loss of the SPC barrier and sympathetic neuropathy in adipose tissue. To ascertain this observation, we performed ex vivo experiments by treating SCG explants with low (10 ng ml^−1^) and high leptin (100 ng ml^−1^) that, respectively, emulate in vitro the states of normo-leptinaemia and hyperleptinaemia^32,35^. We then evaluated the effect of high leptin on the CAV1^+^ SPC barrier and TH^+^ sympathetic neurons by immunofluorescence. Compared with low leptin, high leptin exposure resulted in a significant reduction in CAV1 expression in the SPC barrier and TH intensity in the sympathetic neurons of the SCG explant (Fig. 5d,e). We next checked whether activation of ADRB2 in SPCs can mitigate the effects of high leptin. Notably, co-incubation with a high leptin and a sympathomimetic β_2_ agonist (clenbuterol) could restore the CAV1^+^ SPC barrier integrity and TH^+^ neuron density. This protective effect was abolished by a selective ADRB2 antagonist, butoxamine, indicating that ADRB2 activation is necessary to prevent the SPC and sympathetic neurons loss in hyperleptinaemic conditions (Fig. 5d,e). In contrast, co-treatment with a high dose of leptin and either the ADRB3 agonist (mirabegron) or the ADRB1 agonist (dobutamine) failed to rescue the SPC barrier and sympathetic neuron loss, emphasizing the specificity of the ADRB2-mediated effect (Supplementary Fig. 6a,b). To investigate whether excessive adrenergic signalling alone affects SPCs, we treated the SCG explant with low leptin (10 ng ml^−1^) alone or in combination with high-dose clenbuterol (100 µg ml^−1^ or 200 µg ml^−1^) for 24 h. Neither the SPC barrier integrity nor the TH^+^ neuron density was affected by elevated doses of clenbuterol, suggesting that SPCs are not intrinsically sensitive to supraphysiological adrenergic stimulation (Supplementary Fig. 6c,d). Finally, we evaluated the expression of apoptotic marker TNFR1 in SCG explants treated in vitro with different doses of leptin. Consistent with the in situ data (Fig. 5c), high leptin significantly increased TNFR1 expression both in the SPC barrier and sympathetic neurons, which was restored by β_2_ agonist treatment (Supplementary Fig. 5b,c). Collectively, these data demonstrate that obesity-driven hyperleptinaemia causes apoptosis of SPCs and sympathetic neurons, and that this effect can be prevented by selective activation of β_2_-adrenergic receptors in SPCs.

To investigate whether hyperleptinaemia-induced SPC loss and sympathetic neuropathy can be attenuated in vivo, we treated DIO mice with a human leptin-neutralizing monoclonal antibody (hLep Ab) and an isotype control antibody (Ctrl Ab) for 3 weeks^36^. hLep Ab-treated mice also showed a significant reduction in weight gain compared with Ctrl Ab mice, independently of food intake (Fig. 5f). Notably, we observed a marked increase in CAV1^+^ SPC area and the density of TH^+^ sympathetic axons in the sympathetic nerve bundles isolated from scWAT (Fig. 5g–i) and BAT (Fig. 5g,j,k) of mice treated with hLep Ab compared with Ctrl Ab mice. We next tested whether the administration of ADRB2 agonist (clenbuterol), can rescue SPC loss in vivo. DIO mice were treated with either clenbuterol-containing water or regular drinking water for five consecutive days^37^ (Supplementary Fig. 7a). Consistent with our ex vivo findings, we observed a significant increase in CAV1^+^ SPC area and the density of TH^+^ sympathetic axons in the sympathetic nerve bundles innervating both the scWAT (Supplementary Fig. 7b,d,e) and BAT (Supplementary Fig. 7c,f,g) of the mice that consumed clenbuterol with drinking water compared with the mice that consumed regular drinking water. Collectively, these results confirm that hyperleptinaemia-induced SPC loss and sympathetic neuropathy in obesity can be rescued by the partial reduction of leptin or the administration of an ADRB2 agonist.

### *LEPR*^+^*ADRB2*^+^ SPCs are present in humans, and common polymorphisms of *LEPR* and *ADRB2* show male-specific synergistic interaction with obesity risk

**Fig. 6:**
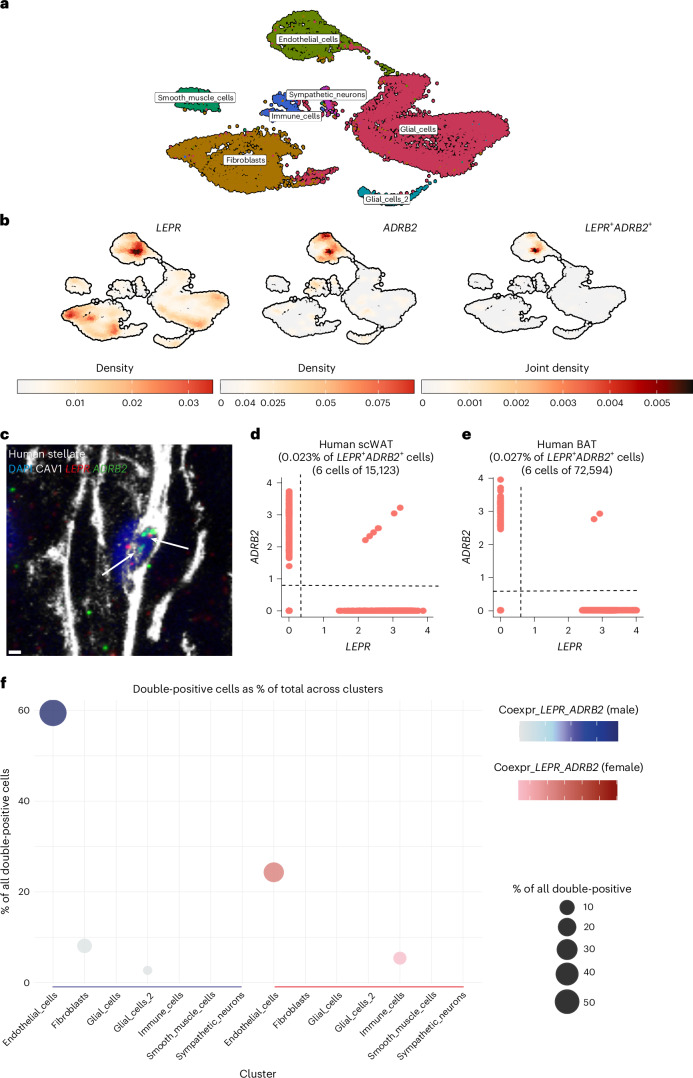
Human SPCs but not adipocytes co-express *LEPR* and *ADRB2*. **a**, Unsupervised clustering of 15,998 nuclei using a resolution of 0.75 and visualized via UMAP. **b**, Density plots showing the expression patterns of *LEPR* and *ADRB2*, and their co-expression across all nuclei. **c**, High-magnification image showing dual labelling of CAV1 protein and *LEPR*/*ADRB2* mRNA in human stellate ganglia. Scale bar, 2 µm. The experiments were repeated twice with similar results. **d**,**e**, Correlograms between *LEPR* and *ADRB2* gene expression in human white (**d**) and brown (**e**) adipose tissues. **f**, Dot plot showing the *LEPR/ADRB2* co-expression in different clusters of male and female human SCG and stellate ganglia dataset. The relative co-expression of *LEPR* and *ADRB2* was quantified across cell clusters by calculating the percentage of double-positive cells within each cluster, normalized to the total number of double-positive cells in the dataset. Co-expression levels are represented by the product of normalized gene expression values (*LEPR* *×* *ADRB2*). Source data

To establish human relevance, we next performed single-nuclei RNA-seq of sympathetic ganglia from human donors, which revealed the distinct population of non-immune and immune cell clusters (Fig. 6a). Notably, within the human sympathetic ganglia, we observed the co-expression of *LEPR* and *ADRB2* exclusively in the endothelial cluster (Fig. 6b). We also confirmed the co-expression of *LEPR* and *ADRB2* mRNA in the CAV1^+^ perineurial barriers of human stellate ganglia, which innervate BAT (Fig. 6c). To evaluate whether this co-expression is conserved in other tissues, we analysed a publicly available scRNA-seq dataset from human subcutaneous white^38^ and brown^39^ adipose tissue. We observed that only 6 cells of 25,123 (~0.02%) co-expressed these genes in the scWAT (Fig. 6d), and a mere 2 cells of 72,594 (~0.002%) co-expressed them in the BAT (Fig. 6e), emphasizing the selective expression of these 2 genes only in human SPCs. To further strengthen the translational relevance, we performed complementary experiments in primary human umbilical vein endothelial cells (HUVECs) overexpressing human *LEPR* and *ADRB2*. Under hyperleptinaemic conditions, LEPR^+^ADRB2^+^ HUVECs exhibited increased apoptosis, which was reproducibly rescued by β_2_-adrenergic stimulation with clenbuterol (Supplementary Fig. 8). Importantly, WT HUVECs showed no apoptotic response under identical conditions, indicating that the effect is dependent on LEPR/ADRB2 expression rather than nonspecific leptin toxicity. Recent genetic association studies in middle-aged Brazilian and Japanese populations reported a synergic interaction between *LEPR* and *ADRB2* variants for being associated with the risk of overweight/obesity^40,41^. We analysed the effect of such *LEPR* and *ADRB2* variants on European individuals from the UK Biobank (UKB) cohort. Consistent with prior reports, we observed that a *LEPR* variant Gln223Arg (rs1137101) or an *ADRB2* variant Gln27Glu (rs1042714) does not have, individually, any statistically significant effects on body mass index (BMI; Supplementary Table 2a). However, we found a significant synergistic effect between *LEPR* Gln223Arg (rs1137101) variant and *ADRB2* Gln27Glu (rs1042714) variant for BMI (beta = 0.04; *P* value = 0.013; Supplementary Table 2a). The same interaction showed a higher effect in the BMI > 25 group (beta = 0.047, *P* value = 0.0056) but was not significant in the BMI < 25 group (Supplementary Table 2a). Sex-stratified analysis further revealed that a significant synergistic interaction between *LEPR* Gln223Arg (rs1137101) variant and *ADRB2* Gln27Glu (rs1042714) variant was driven by only men for BMI (BMI > 25 group (beta = 0.057, *P* value = 0.04), BMI < 25 group (beta = 0.061, *P* value = 0.03); Supplementary Table 2b,c). No significant interaction was observed in women in both BMI groups. The interaction frequency of *LEPR* Gln223Arg (rs1137101) variant and *ADRB2* Gln27Glu (rs1042714) variant was higher (53%) in men in the BMI > 25 group compared with all other groups (Supplementary Table 2b,c). Importantly, this sex-specific interaction aligns with the sexual dimorphism we observed in *LEPR/ADRB2* co-expression within human SPCs (Fig. 6f), supporting a potential link between these cells and the observed genetic association with male obesity. Consistently, we observed no difference in body weight between female *Lepr*^Cre^: *Adrb2*^fl/fl^ and control *Adrb2*^fl/fl^ mice in both chow-fed and HFD-challenged states (Supplementary Fig. 9), which resonates with the sexual dimorphism observed in the human cohort.

To report on a potential structural interaction, we also modelled the interaction between the D1 domain as well as D1–D7 domains from LEPR (Q223R, Gln223Arg) with ADRB2 (Q27E, Gln27Glu) using AlphaFold2-multimer. The Q223R polymorphism is located at the D1 domain of *LEPR*. Structural predictions showed poor interaction metrics, clustering the D1 domain in the cytoplasmic side of the ADRB2, which is highly implausible because the D1 is located extracellularly (Supplementary Fig. 10a,b). This observation likely reflects the known challenges of modelling membrane protein complexes with AF2 due to inconsistencies in transmembrane segment location^42–44^. We further performed molecular dynamics simulations for both *ADRB2* and *LEPR* polymorphisms to assess any differences in ligand binding. For *ADRB2*, both WT and the Q27E polymorphism maintained ~80% of native adrenaline contacts, with stable binding observed over 1,000 ns, aligning with prior reports of negligible functional change^45^ (Supplementary Fig. 10c). For *LEPR*, while the Q223R polymorphism showed increased local flexibility in the D1 domain, no significant differences were observed in leptin binding in full-length receptor simulations, with both WT and Q223R retaining ~75% of native contacts over 8,000 ns (Supplementary Fig. 10d). These results suggest that neither mutation substantially alters ligand binding stability within the simulated timescales.

Together, the genetic data point to a male-specific interaction between *LEPR* and *ADRB2* variants in the association with obesity, which aligns with SPC biology; however, structural and simulation analyses do not support a direct physical interaction between receptors.

**Fig. 7:**
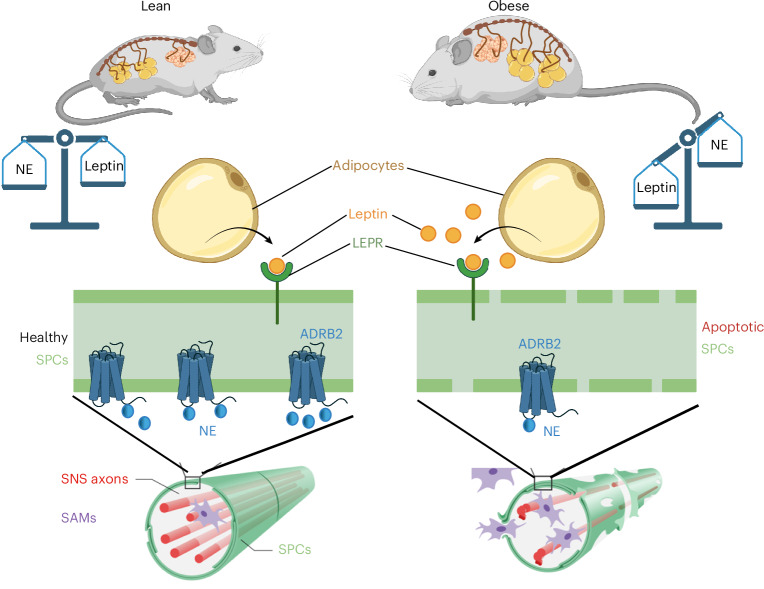
Graphical illustration of our working model. The *Lepr*^+^*Adrb2*^+^ SPCs can sense and integrate leptin and NE signalling in adipose tissue that form the afferent and efferent arms of the neuroendocrine loop of leptin action, respectively. In obesity, when leptin signalling exceeds the NE released by sympathetic nerves, SPCs undergo apoptosis, which erodes the neuroprotective perineurial barrier, exposing sympathetic neurons to pro-inflammatory cues. Altogether, this contributes to sympathetic neuropathy and disrupts adipose tissue homeostasis. SAMs, sympathetic-associated macrophages; SPCs, sympathetic perineurial cells; LEPR, leptin receptor; ADRB2, β_2_-adrenergic receptor. Illustration created in BioRender; Sarker, G. https://biorender.com/oby2bb7 (2026).

Overall, our data support a model whereby SPCs coordinate leptin and sympathetic signalling to preserve adipose sympathetic integrity and maintain metabolic homeostasis, while obesity-associated hyperleptinaemia disrupts this regulatory circuit through SPC apoptosis (Fig. 7).

## Discussion

Systems control theory posits that stable negative feedback requires proportional engagement of afferent and efferent arms of a loop. In the neuroendocrine loop of leptin action, adipocyte-derived leptin constitutes the afferent signal to the brain, whereas sympathetic outflow to adipose tissues represents the efferent arm. While the central components of this loop have been extensively studied, peripheral mechanisms that might influence its operating balance have remained less well defined.

In searching for a cell population capable of responding to both leptin and sympathetic tone within peripheral ganglia, we identified a perineurial endothelial population in sympathetic ganglia and adipose-associated nerve bundles that shows enriched co-expression of *Lepr* and *Adrb2*. Across 16 publicly available single-cell datasets covering 24 mouse organs, analysed using a uniform pipeline, *Lepr*/*Adrb2* double-positive cells were preferentially enriched in sympathetic ganglia relative to other tissues, where such cells were rare and typically at frequencies indistinguishable from background^18,20^. Independent RNAscope validation across multiple organs further supported this preferential enrichment. Together, these data position *Lepr*^+^*Adrb2*^+^ SPCs as a specialized peripheral population with the capacity to respond to both circulating leptin and local β_2_-adrenergic signal^46^.

Our functional experiments indicate that ADRB2 signalling in LEPR^+^ SPCs is required to sustain energy expenditure and thermogenic activity. Conditional deletion of *Adrb2* in *Lepr*^Cre^-expressing SPCs resulted in reduced energy expenditure, lower BAT temperature, diminished thermogenic gene expression and increased adiposity, without alterations in food intake. These findings establish a causal role for β_2_-adrenergic signalling in this cell population in maintaining metabolic homeostasis. In particular, these results provide new insights into ADRB2’s regulation of metabolic rate and fat mass reduction, which so far focus on postsynaptic effects on thermogenic adipocytes^47^, whereas SPCs act presynaptically by ensheathing sympathetic ganglia and nerve bundles, preserving their normal function^5^.

In parallel, we observed that DIO and associated hyperleptinaemia are accompanied by apoptosis of SPCs, erosion of the perineurial barrier, and sympathetic neuropathy in adipose tissue. Importantly, partial leptin reduction in vivo or selective β_2_-adrenergic agonism both prevented SPC loss and restored sympathetic integrity. Ex vivo, supraphysiological leptin induced apoptotic signatures in SPCs that were specifically counteracted by β_2_ agonism, but not by β_1_ or β_3_ stimulation. These complementary genetic and pharmacological data support a model in which leptin excess and β_2_-adrenergic tone exert opposing influences on SPC viability. We note, however, that currently available Cre-based strategies do not allow selective deletion of both *Lepr* and *Adrb2* specifically at their intersection within SPCs. Crossing endothelial Cre drivers with floxed alleles would target the union rather than the co-expression domain of these receptors. Thus, while our data support reciprocal effects of LEPR and ADRB2 in SPCs, they do not directly interrogate cell-autonomous co-requirements of both receptors within the same cell in vivo. This could represent a technical limitation and an avenue for future refinement using intersectional genetic tools.

Most conceptual models of leptin resistance have focused on hypothalamic mechanisms regulating appetite^48^. Our findings suggest that peripheral alterations in sympathetic ganglia and their perineurial barrier may also contribute to impaired energy expenditure during obesity. In this framework, sustained hyperleptinaemia may progressively compromise SPC integrity, weaken the neuroprotective perineurial barrier, exposing sympathetic neurons to pro-inflammatory cues^49^, and reduce sympathetic support of adipose tissue thermogenesis (Fig. 7). Rather than redefining a systemic ‘set point,’ these data support the existence of a peripheral modulatory node that can influence the efficiency of sympathetic output under conditions of leptin excess^50^.

The translational relevance of this axis is supported by the presence of LEPR^+^ADRB2^+^ SPCs in human sympathetic ganglia and by the male-specific synergistic interaction between common *LEPR* and *ADRB2* variants associated with increased BMI in the UKB cohort, replicating observations in independent populations^40,41^. Although genetic association does not establish causality at the cellular level, the convergence of human single-nucleus transcriptomics, endothelial overexpression assays, and population genetics strengthens the plausibility of an LEPR–ADRB2 axis in human metabolic regulation. To assess the potential for a direct structural basis underlying this interaction, we conducted structural modelling and molecular dynamics simulations. The results suggest that neither mutation substantially alters ligand binding stability within the simulated timescales. However, limitations in sampling timescales, simulation resolution, and exclusion of membrane and additional binding partners should be considered when interpreting these findings^51,52^. Alternatively, the synergistic interaction between *LEPR* and *ADRB2* polymorphisms on BMI could be explained by the cross-talk within downstream signalling pathways^53^. Future studies will determine how LEPR and ADRB2 variants in SPCs alter leptin–noradrenergic signalling to impact body weight gain. This will provide new insights on how to dually target LEPR and ADRB2, potentially with unimolecular polypharmacy, to bypass central leptin resistance beyond suppression of food intake in a large patient cohort.

In summary, our findings identify LEPR^+^ADRB2^+^ SPCs as a peripheral component of the neuroendocrine network engaged by leptin action, with potential implications for understanding aspects of leptin resistance beyond the regulation of food intake. By influencing perineurial barrier integrity and sympathetic support of adipose tissue, this cell population may contribute to the regulation of energy expenditure and to the progression of obesity under conditions of chronic hyperleptinaemia and sympathetic neuropathy.

## Methods

### Animals

C57BL/6 WT mice at 6–8 weeks old were purchased from Charles River. *Lepr*^*Cre*^ mice (*Lepr*^*tm2(cre)Rck*^; stock number 008320), Rosa26-LSL-ChR2-YFP mice (stock number 012-569) and *ob*/*ob* mice (stock number 000632) were purchased from the Jackson Laboratory, and *Adrb2*^fl/fl^ mice were kindly provided by G. Karsenty, Columbia University, USA. Mice were bred in-house to produce homozygous *Lepr*^*Cre*^: *Rosa26-LSL-ChR2-YFP* mice and *Lepr*^*Cre*^: *Adrb2*^fl/fl^ mice. Mice were housed in groups of 2–5, unless fighting or barbering was observed. The room in which animals were housed had a 12-h light–dark cycle: light during the day and dark during the night, switching at 7:00 and 19:00. Ambient room temperature (RT) was maintained at 22°C ± 2 °C and humidity at 50% ± 20%. Mice had unrestricted access to water and a regular chow diet (irradiated 2916 Teklad diet; 63% carbohydrate, 23% protein and 4% fat), unless mentioned otherwise. Both male and female mice were used in this study. All experiments were conducted in accordance with the United Kingdom Animal Scientific Procedures Act 1986 under personal and project licences granted by the United Kingdom Home Office and approved by the local Department of Physiology, Anatomy and Genetics (University of Oxford) ethical review committee.

### Antibodies, reagents and drugs

Antibodies were obtained from the following vendors: rabbit anti-TH (Millipore, AB152; 1:500 dilution), chicken anti-TH (Aves Lab, TYH, TH1205; 1:500 dilution), goat anti-GFP (Abcam, ab6673; 1:1,000 dilution), chicken anti-GFP (Abcam, ab13970; 1:1,000 dilution), rabbit anti-caveolin-1 (CAV1; Cell Signaling, D46G3; 1:500 dilution), rabbit anti-glucose transporter (GLUT1; Abcam, ab150299; 1:200 dilution), rabbit anti-TNFR1 (Invitrogen, PA595585; 1:500 dilution), rabbit anti-VE-cadherin (Life Technologies, 361900; 1:100 dilution), goat F(ab) anti-mouse IgG (H + L; Abcam, ab6668; 1:500 dilution), mouse anti-VEGFR2 (Santa Cruz Biotechnology, Sc-6251; 1:500 dilution), rabbit anti-NeuN (Abcam, ab177487; 1:500 dilution), rabbit anti-Des (Abcam, ab15200; 1:500 dilution), rat anti-CD31 (BioLegend, 102501; 1:2000 dilution), goat anti-rabbit IgG (H + L) secondary antibody, Alexa Fluor 647 (Invitrogen, A11010; 1:500 dilution), goat anti-rabbit IgG (H + L) secondary antibody, Alexa Fluor 546 (Invitrogen, A11035; 1:500 dilution), goat anti-chicken IgG (H + L) secondary antibody, Alexa Fluor 594 (Invitrogen, A11042; 1:500), goat anti-chicken IgY (H + L) secondary antibody, Alexa Fluor 647 (Invitrogen, A21449; 1:500 dilution), goat anti-chicken IgY (H + L) secondary antibody, Alexa Fluor 546 (Invitrogen, A11040; 1:500 dilution), goat anti-mouse IgG (H + L) secondary antibody, Alexa Fluor 594 (Invitrogen, A11005; 1:500 dilution), goat anti-chicken IgY (H + L) secondary antibody, Alexa Fluor 488 (Invitrogen, A-11039; 1:500 dilution), goat anti-rabbit IgG (H + L), Alexa Fluor 488 (Invitrogen, A11034; 1:500 dilution), donkey anti-goat IgG (H + L) secondary antibody, Alexa Fluor 647 (Invitrogen, A21447; 1:500 dilution), donkey anti-chicken IgY (H + L) secondary antibody, Alexa Fluor 647 (Stratech, 703-605-155; 1:500 dilution), donkey anti-goat IgG (H + L) secondary antibody, Alexa Fluor 488 (Invitrogen, A11055; 1:500 dilution), donkey anti-chicken IgG (H + L) secondary antibody, Alexa Fluor 488 (Jackson ImmunoResearch, 703-545-155; 1:500), donkey anti-rabbit IgG (H + L) secondary antibody, Alexa Fluor 546 (Invitrogen, A10040) and DAPI (Invitrogen, D1306; 1:500 dilution). All primary antibodies were validated by the manufacturers (see datasheets and manufacturer websites). Additional validation was supported by previous literature^1,54^. Recombinant mouse leptin was obtained from Amylin Pharmaceuticals (R&D Systems, 498-OB). Clenbuterol hydrochloride (C5423), dobutamine hydrochloride (D0676), mirabegron (SML2480) and butoxamine hydrochloride (sc-234233) were purchased from Sigma-Aldrich, Merck and Santa Cruz Biotechnology, respectively.

### Dietary challenge

When C57BL/6 WT mice or both *Lepr*^*Cre*^: Rosa26-LSL-ChR2-YFP mice, *Lepr*^Cre^: *Adrb2*^fl/fl^ mice, and respective controls *Lepr*^Cre^ and *Adrb2*^fl/fl^, reached 8 weeks of age, a regular chow diet was replaced with a HFD. The HFD was a defined, lard-based diet with 60% energy from fat, 20% energy from carbohydrate and 20% energy from protein (Research Diets, D12492). This feeding regime lasted 10–12 weeks, with the exact duration specified in the figure legends. For the normal diet challenge, the mice were kept on a regular chow diet since weaning, and the body weight and food intake were measured from the age of 6 weeks to the age of 16 weeks. Food intake was exchanged and measured weekly every Wednesday morning (at the beginning of the light cycle) from the onset of the dietary challenge. Food intake was measured by subtracting the weight of the food left in the cage from the original weight of the food added to the cage. The average daily food intake per mouse was then calculated for each week. The results represent the average daily food intake per mouse in grams. Body weight was measured at the same time as food intake was measured.

### Metabolic monitoring

The Promethion Core CGF system (Sable Systems International, North Las Vegas) was used to evaluate metabolic profiles and physical activity of the mice. Mice were housed in individual cages with free access to food and water, under a 12-h light–dark cycle, at a controlled RT (21–23 °C) and humidity. After acclimatization for 48 h, levels of VO_2_, VCO_2_, energy expenditure, RER and locomotor activity were measured every 10 min for 3–4 days. The body weight and food intake were measured immediately before placement into the indirect calorimetry cage and again upon removal. Promethion data analysis software package, Macro Interpreter (version 23.4.0) by Sable System, was used to conduct primary data processing. Data for energy expenditure, VO_2_ and VCO_2_ were analysed with general linear model ANCOVA in SPSS (IBM) using body weight as a covariate, as previously suggested^55,56^. RER and total activity were analysed by ANOVA.

### Temperature measurements

BAT temperature was measured using an E96 Advanced Thermal Imaging Camera (FLIR). The interscapular region of the mice was shaved 2 days before thermal imaging to avoid stress-induced BAT activity. The thermal images of single-housed freely moving mice were captured at 20 cm from the cage at 22 °C. All thermal imaging analysis was carried out under blinded conditions using FLIR Tools software. The average interscapular surface and tail temperature was calculated from at least five thermal images of each mouse as shown^56^. Rectal temperature was measured using a thermal probe (Physitemp). The core body temperature of unanaesthetized, awake mice was measured using a rectal probe (Precision) under normal housing conditions (22 ± 2 °C). The probe was lubricated with Vaseline before insertion into the anus (1.5 cm). During the measurements, mice were lightly restrained in a cotton glove for no more than 20 s.

### Body composition

Total body fat mass and lean mass were assessed by quantitative nuclear magnetic resonance spectroscopy using a fully automatic EchoMRI system (Echo Medical Systems) as shown^56,57^. After completion of the normal diet and HFD challenges, mice were euthanized, and fat tissues, including inguinal (scWAT), epididymal (visceral WAT) and interscapular BAT, and key skeletal muscles such as quadriceps, gastrocnemius and soleus were isolated and weighed. Samples were snap-frozen and stored at −80 °C until processing.

### Leptin-neutralizing antibody treatment

We followed the treatment regime documented in a previous report^36^. WT mice were fed with a HFD for 12 weeks to induce obesity. The mice were then divided into two groups and treated with either a human leptin-neutralizing monoclonal antibody (kindly provided by P. Scherer, The University of Texas Southwestern Medical Center) or an isotype control antibody (a human IgG1 monoclonal antibody). The antibodies were administered via intraperitoneal injection twice a week for 3 weeks, while mice were kept on a HFD during the whole period. Body weight and food intake were measured weekly. After 3 weeks of antibody treatment, mice were euthanized for the collection of sympathetic nerve fibres from subcutaneous WAT and interscapular BAT.

### Clenbuterol administration

For chronic clenbuterol treatment, we followed the treatment regimen described by Meister et al.^37^. Mice were first challenged with a HFD for 12 weeks to become obese. HFD-induced obese mice (DIO) were then randomly assigned to two groups: (1) the control group, which received regular drinking water, and (2) the clenbuterol group, which received clenbuterol (Sigma-Aldrich, C5423; 30 mg l^−1^) via drinking water for 5 days. Both groups were kept on a HFD throughout the experiment. During the 5-day treatment period, no difference in water intake was observed between the clenbuterol-treated mice (3.45 ml ± 0.95 ml per mouse per day) and the control mice (3.83 ml ± 0.25 ml per mouse per day). Following the treatment period, mice were euthanized, and the sympathetic nerve fibres were isolated from subcutaneous WAT and interscapular BAT, fixed overnight and stored at 4 °C in 1× PBS until further processing.

### Tissue collection and dissociation for scRNA-seq

scRNA-seq was performed as previously described^58^. Ten adult C57BL/6 males were euthanized at 24 weeks of age, and sympathetic ganglia (SCG and stellate ganglia) were quickly extracted under a stereomicroscope. Tissue was digested for 30 min with collagenase (2.5 mg ml^−1^) and DNase (5 U ml^−1^) in HBSS at 37 °C, washed and further digested with trypsin (0.25%) for 30 min at 37 °C with shaking. The samples were mechanically triturated, and the cell suspension was then filtered through a 40-μm cell strainer and centrifuged at 400*g* for 5 min. After aspiration of the supernatant, the pellet was resuspended in DMEM media with 10% FBS and one volume of 40% wt/vol iodixanol solution. The cell suspension was carefully overlaid with an Optiprep gradient composed of 3 ml 22% wt/vol iodixanol solution and 0.5 ml DMEM and centrifuged at 800*g* for 25 min. The viable cells were carefully collected from the top interface, and the cell suspension was concentrated to 300 µl by removing the supernatant.

### Library preparation, sequencing and alignment

The cell suspension (approximately 10,000 cells per channel) was then loaded onto the single-cell 3′ chip and placed on a 10x Genomics chromium controller instrument to generate single-cell GEMs. scRNA-seq libraries were prepared using the chromium single-cell 3′ library & cell bead kit according to the manufacturer’s protocol. Libraries were sequenced with an Illumina NextSeq 500 platform to a depth of approximately 300 million reads per library with 2 × 50-bp read length. The Cell Ranger Single Cell Software Suite v.2.0.1 was used to perform sample de-multiplexing, alignment, filtering and unique molecular identifier counting.

### Cell clustering and cell-type annotation

The cluster identities and filtered gene matrices generated by Cell Ranger software were used as input into the open-source R toolkit Seurat (v.4.1.2)^59^ to produce UMAP, feature plots, violin plots of the mean and variance of the mean and variance of gene expression density. Cell barcodes with <500 transcripts detected or >25% mitochondrial gene expression were first filtered out as low-quality cells. The gene counts for each cell were divided by the total gene counts for the cell and multiplied by a scale factor of 10,000, then log_2_ transformation was applied to the counts. The FindVariableFeatures function was used to select variable genes with default parameters. The ScaleData function was used to scale and centre the counts in the dataset. Principal component analysis was performed on the variable genes, and 20 principal components were used for cell clustering (resolution of 0.5) and UMAP dimensional reduction. The cluster markers were found using the FindAllMarkers function, and cell types were manually annotated based on the cluster markers in the literature. Module scores were calculated using the AddModuleScore function with default parameters and used to validate certain cell-type annotations. To calculate the sample composition based on cell type, the number of cells for each cell type from each sample was counted. The counts were then divided by the total number of cells for each sample and scaled to 100% for each cell type. In the co-expression analysis, the cells having the expression threshold for both genes more than 0.75 are considered to be positive (True) for normalized co-expression. For visualization of gene expression, count data for each run was denoised using a deep count autoencoder^60^, with default parameters, to be then merged before plotting.

### Immunofluorescence microscopy

Mice were perfused with 1× PBS, and sympathetic ganglia and nerve bundles were extracted under the stereomicroscope and fixed in 4% paraformaldehyde (PFA) overnight at 4 °C with shaking. For images in Figs. 1d and 4c,d,f, we used frozen sections, and the fixation step was followed by cryoprotection in 30% sucrose (Alfa Aesar). Sections (15 µm) were obtained in a Leica Cryostat CM3050 S. For images in Fig. 3c,e, whole-mount tissues were used. Both frozen sections and whole-mount tissues were washed with PBS for 10 min, which was followed by incubation in a blocking and permeabilization solution (3% BSA, 2% goat or donkey serum based on the host species of secondary antibodies, 0.1% Tween and 0.1% sodium azide in 1× PBS) for 1 h at RT with (whole mounts) or without (frozen sections) shaking. The samples were then incubated with primary antibodies overnight at 4 °C with (whole mounts) or without (frozen sections) agitation. The following dilutions of primary antibodies were used: anti-GFP (1:1,000 dilution), anti-TH (1:500 dilution), anti-CAV1 (1:1,000 dilution), anti-GLUT1 (1:200 dilution), anti-TNFR1 (1:500 dilution), anti-VEGFR2 (1:500 dilution), anti-TUBB3 (1:1,000 dilution) and anti-VE-cadherin (1:100 dilution). The anti-GFP antibody was used to detect the YFP-tagged LEPR cells. After washing, secondary antibodies (1:500 dilution) were applied for 1 h at RT, with or without (in the case of frozen sections) shaking. After incubation with secondary antibodies, tissues were washed with 1× PBS and was followed by incubation with DAPI (1:1,000 dilution) for 5 min (frozen sections) or 30 min (whole mounts). After DAPI staining, tissues were washed and mounted with ProLong Gold Antifade reagent (Life Technology, P36930). The slides were dried overnight at RT and stored at 4 °C.

Images were acquired with a Zeiss LSM 780 inverted confocal microscope. Analysis and quantification of acquired images were performed using Fiji^61^. For representative images, linear adjustments were made to brightness and contrast, whereas for quantification, all channels were kept unchanged. Two to five randomly selected samples per mouse from three to seven independent mice from each experimental group were quantified. High-resolution images were acquired with a confocal laser scanning microscope (Zeiss LSM 980), equipped with an Airyscan detection unit. To maximize the resolution enhancement, we used high-NA oil immersion alpha Plan-Apochromat ×40/1.4-NA Oil DIC M27 objectives (Zeiss). Detector gain and pixel dwell times were adjusted for each dataset, keeping them at their lowest values to avoid saturation and bleaching effects.

### RNAscope dual ISH and immunofluorescence

Mice at 8 weeks age were perfused transcardially with PBS (pH 7.4). Tissues (brain, SCG, stellate ganglia, sympathetic nerve bundles, BAT, scWAT, VAT, heart, pancreas, muscle, liver, small intestine, trachea, lung and bladder) were immediately removed from the perfused mice and fixed in 10% neutral buffered formalin (Sigma-Aldrich) overnight at RT before being transferred to 70% ethanol. The hypothalamic region was isolated from the whole fixed brain as described previously^62^. Samples were dehydrated and embedded in paraffin using standard procedures and then sectioned at 5 µm onto SuperFrost Plus slides (Fisher Scientific). Dual ISH and immunofluorescence was performed using the RNAscope LS Multiplex Fluorescent Reagent Kit (323275) and RNA-Protein Co-detection Ancillary Kit (323180) together with Opal 520/570/690 Fluorophore Reagent pack detection (1:1,000 dilution, FP1487001KT/FP1488001KT/FP1497001KT) on the Leica BOND RX Fully Automated Research Stainer (Leica) according to the manufacturer’s instructions. The primary antibodies used for immunofluorescence were rabbit anti-CAV1 (1:10,00 dilution), rabbit anti-GLUT1 (1:200 dilution), rabbit anti-TH (1:1,000 dilution), rabbit anti-PECAM1 (1:2,000 dilution) and rabbit anti-NeuN (1:500 dilution) combined with a BrightVision goat anti-rabbit poly-HRP detection system (Immunologic). For ISH, the following RNAscope target probes from ACD were used: *Lepr* (Mm-Lepr-tv1, ACD 471178) and *Adrb2* (Mm-Adrb2-XRn-C2, ACD 1172758-C2), positive control probes: *Th* (Mm-Th-XRn-C2, ACD, 870118-C2) and *Polr2a* (Mm-Polr2a, ACD 312478), or negative control probe DapB (ACD, 312038). Slides were counterstained with spectral DAPI and then cover-slipped with ProLong Gold Antifade reagent (Thermo Fisher Scientific).

Fluorescence slide scans were acquired with an Olympus VS200 slide scanner (Olympus) using a ×40 (0.95-NA) air objective and a DAPI/CY3/CY5 filter set. Images were visualized and processed with Olympus OlyVIA 3.8 software. High-resolution images were captured in ×40 MPLX_4Y Airyscan or ×40 Airyscan SR mode. Image quantification was performed using Fiji^61^. Cell counting was performed manually. To quantify the *Lepr*^+^*Adrb2*^+^ neurons or endothelial cells in the arcuate hypothalamus, trachea, lung and bladder images were captured from 3–8 sections per mouse, and a total of three to five mice were independently analysed. The percentages of *Lepr*^+^, *Adrb2*^+^ or *Lepr*^+^*Adrb2*^+^ cells in NeuN^+^ neurons or PECAM1^+^ endothelial cells were calculated by dividing the total fluorescence *Lepr*^+^ *Adrb2*^+^ or *Lepr*^+^*Adrb2*^+^ mRNA counts by the total number of NeuN^+^ or PECAM1^+^ cells, respectively. Percentage positive cell values were imported into Prism (GraphPad) for graphing and statistical analysis. As previously described^63^, RNAscope ISH was performed to detect *ADRB2* and *LEPR* mRNA in post-mortem human stellate ganglia using the RNAscope Multiplex Fluorescent v2 Assay (323100, Advanced Cell Diagnostics, a Bio-Techne brand), following the manufacturer’s instructions. Fixed frozen ganglia were cryosectioned at 10 µm, mounted on Superfrost Plus slides, and hybridized with probes targeting Hs-ADRB2 (450641, detected in Cy5) and Hs-LEPR (410371-C2, detected in Cy3). After RNAscope, immunofluorescence for caveolin-1 (CAV1) was performed with rabbit anti-CAV1 (D46G3) XP monoclonal antibody (Cell Signaling Technology, 3267; 1:500 dilution). The sections were imaged using confocal microscopy (Leica TCS SP8) and a motorized scanning stage fluorescence microscope (AxioObserver Z1, Zeiss).

### Electron microscopy

Sympathetic nerve bundles dissected from scWAT of lean and HFD-fed obese mice were fixed with 2% PFA and 2.5% glutaraldehyde (Electron Microscopy Services) in 0.1 M sodium cacodylate buffer. After several rinses in sodium cacodylate buffer, samples were postfixed in 1% osmium tetroxide, 1.5% potassium ferricyanide in 0.1 M sodium cacodylate buffer for 2 h on ice. Samples were dehydrated with a crescent concentration of ethanol, washed with propylene oxide, and infiltrated in a mixture of propylene oxide/epoxy resin overnight. The resin was then substituted with fresh epoxy resin, and samples were embedded in silicone moulds. Then, after being cured for 48 h at 60 °C, resin blocks were cut into ultrathin sections (70–90 nm) using an ultramicrotome (UC7, Leica microsystem, Vienna, Austria), longitudinal sections of nerve bundles were collected on copper formvar carbon-coated slot grids, stained with uranyl acetate and Sato’s lead solutions and observed in a Transmission Electron Microscope Talos L120C (FEI, Thermo Fisher Scientific) operating at 120 kV. Images were acquired with a Ceta CCD camera (FEI, Thermo Fisher Scientific). For quantification of the perineurial layers for each fibre, 20 random images were acquired along the external fibre profile and quantified manually using ImageJ software^61^.

### Sympathetic ganglia explant cultures

Ganglia explant culture was performed as previously described^49^. SCG and stellate ganglia were removed from mice aged 8 weeks under a stereomicroscope and placed in DMEM (Invitrogen). Ganglia were cleaned from the surrounding tissue and transferred into eight-well chamber slides (µ-slide 8 well, ibidi, 80826) that were previously coated with poly-D-lysine (Sigma-Aldrich) in accordance with the manufacturer’s instructions. Ganglia were then covered with 5 µl of Matrigel (BD Bioscience) and incubated for 7 min at 37 °C. The culture medium (DMEM without phenol red (Invitrogen), 10% FBS (Invitrogen), 2 mM l-glutamine (Biowest), nerve growth factor (1:1,000 dilution; Sigma-Aldrich) and 1% penicillin–streptomycin) was subsequently added. Twelve ganglia explant cultures (six SCG and six stellate) were prepared for each condition. Ganglia were cultured for a minimum of 24 h before further manipulation. After 24 h, ganglia cultures were washed once with the culture media. The stimulation protocol in Supplementary Fig. 6b was performed for 24 h with the following concentrations of drugs: low leptin (10 ng ml^−1^), high leptin (100 ng ml^−1^)^32^, high leptin (100 ng ml^−1^) + a sympathomimetic Adrb2 agonist (clenbuterol hydrochloride; 10 µg ml^−1^)^64^, high leptin (100 ng ml^−1^) + an Adrb3 agonist (Mirabegron; 10 µM)^65^ and high leptin (100 ng ml^−1^) + an Adrb1 agonist (dobutamine; 50 µM)^66^. For Supplementary Fig. 6d, the explant was stimulated for 24 h with the following concentrations: low leptin (10 ng ml^−1^), low leptin (10 ng ml^−1^) + high clenbuterol (100 µg ml^−1^) and low leptin (10 ng ml^−1^) + high clenbuterol (200 µg ml^−1^). In Fig. 5e, the stimulation protocol was applied for 24 h using the following drug concentrations: low leptin (10 ng ml^−1^), high leptin (100 ng ml^−1^), high leptin (100 ng ml^−1^) + clenbuterol (10 µg ml^−1^) and high leptin (100 ng ml^−1^) + clenbuterol (10 µg ml^−1^) + butoxamine (10 µM). Butoxamine was added 30 min before the administration of clenbuterol^67^. The next day, the cultures were washed once with ice-cold 1× PBS for 5 min. The explant cultures were then fixed with 4% PFA overnight at 4 °C with agitation, cryoprotected in 30% sucrose and subsequently sectioned by cryostat for immunostaining.

### Plasma isolation and protein extraction from fat tissue for biochemical assays

For terminal blood collection, mice were euthanized via intraperitoneal injection with 10 μl per gram body weight pentobarbital. Blood was collected from the left ventricle using 25-gauge needles and syringes pre-coated with 100 mM EDTA. The blood was then added into tubes containing 5 μl of 100 mM EDTA and centrifuged at 1,000g at 4 °C for 15 min to separate plasma. The supernatant was carefully transferred to a new tube. The concentration of leptin was determined using a Mouse Leptin ELISA Kit (Invitrogen, KMC2281).

Proteins were extracted from interscapular BAT and scWAT fat tissues as previously described^68^. Briefly, fat depots were dissected and placed into homogenizing tubes filled with 50 μl of lysis buffer per 30 mg of tissue. The lysis buffer consisted of 4 mM sodium metabisulfite (Affa Aesar, A17351), 1 mM EDTA (Invitrogen, 15575-020), 0.01 M HCL and 1× protease inhibitor (CST 5872). Samples were homogenized using a Precellys 24 homogenizer at 5,500 rpm for 60 s. Lipids were removed from the tissue homogenate by centrifugation at 17,000*g* at 4 °C for 15 min, and the clear phase was collected. The centrifuge step was repeated three times to remove lipids completely. The concentration of noradrenaline in the scWAT and BAT was determined using a noradrenaline ELISA kit (Ultra Sensitive, Immusmol, BA-E-5200R).

### Gene expression analysis

Total RNA from BAT tissue was extracted using the TRIzol–chloroform method (Invitrogen) according to the manufacturer’s instructions and treated with DNase (Biolabs) for 30 min at 37 °C to exclude any DNA contamination. Following RNA extraction, 1 µg of total RNA was converted to cDNA using SuperScript II Reverse Transcriptase (Invitrogen, 18064-022). The RT–qPCR reactions were performed using the SYBR Green PCR master mix (LifeTech 4368706). All real-time qPCR reactions were run in duplicate on 96-well plates using the StepOnePlus Real-Time PCR System. Data were calculated using the ∆∆Ct method and normalized against the expression of *Hprt.* Normalized expression levels were presented relative to their appropriate control (=1). The primers are listed in Supplementary Table 3.

### TUNEL assay

Freshly isolated sympathetic ganglia were fixed in 4% PFA overnight at 4 °C, embedded in OCT compound and sectioned at a thickness of 4 µm. Sections were then incubated in 0.1% Triton X-100 for 2 min, followed by the addition of Proteinase K solution for 15 min. The sections were subsequently blocked in 3% BSA in PBS for 30 min. Following pretreatments, TUNEL staining was performed using the in situ BrDU-Red DNA Fragmentation (TUNEL) assay kit from Abcam (ab66110) according to the manufacturer’s instructions. Slides were imaged using a Zeiss LSM 980 confocal microscope.

### Quality control in the UKB

*LEPR* and *ADRB2* variants were extracted from the whole-exome sequencing data from 470,000 UKB individuals. Variants were required to pass the following criteria to be selected: 10× minimum coverage, genotype quality score ≥ 20, quality score ≥ 30, 0.2 ≤ heterozygote alternative allele ratio ≤ 0.8, 0.8 ≤ homozygote alternative allele ratio ≤ 1.0, mapping quality score ≥ 40 and DPGLnexus variant status = PASS. Furthermore, for both genes, variants with minor allele frequency < 0.01 and Hardy–Weinberg equilibrium *P* > 1 × 10^−^^12^ or variants with minor allele frequency ≥ 0.01 and Hardy–Weinberg equilibrium *P* > 1 × 10^−6^, outside the targeted CCDS boundaries (https://www.ncbi.nlm.nih.gov/projects/CCDS/CcdsBrowse.cgi/) and with a call rate < 0.9 were removed before analysis.

### Interaction between *LEPR* and *ADRB2* in the UKB

To mitigate a possible increase of variance estimates due to relatedness, we sought to remove individuals up to third-degree relatives from our analyses using KING (v2.2.3)^69^. We then selected a total of 346,177 European unrelated individuals with BMI measures to investigate gene–gene interaction analyses between *LEPR* and *ADRB2* genes. Qualifying single-nucleotide polymorphisms (SNPs) were tested for their association with the continuous biomarker using a linear regression with an interaction variable and adjusted for age, sex, array and PC1–PC10 using the SNPassoc R package^70^. The interaction model used was: Trait ~ *β*_0_ + *β*_1 _× SNP_1_ + *β*_2 _× SNP_2_ + *β*_3 _× (SNP_1 _× SNP_2_) + *β*_*n* _× covariate_*n*_. The significance threshold for interaction was defined by traits using a Bonferroni correction *P* = 0.05/(*n* SNP).

### Protein modelling and molecular dynamics

The sequences we fed into AF2 were retrieved from UniProt for LEPR (P48357) and ADRB2 (P07550). The sequence span for the analysed constructs was the following: LEPR D1 (124-236), LEPR D1–D7 (124–830) and ADRB2 (1–413). AF2 predictions were performed using the ColabFold implementation^71^ installed locally, using default parameters, and MMseqs2 (ref. ^72^) for creating multiple sequence alignments. The models were subsequently relaxed with AMBER using ColabFold, using default parameters. AF2 mainly reports two metrics for data analysis: the predicted local distance difference test and the predicted alignment error (PAE). A predicted local distance difference test above 90 is considered very confident. Values between 70 and 90 are considered reasonably accurate, while those below 70 are less confident. The PAE metric provides a visualization of the errors at the residue-residue level. The PAE matrix provides a granular view of where the model might be uncertain about the distances between residues. It is a value between 0 and 30 (Å), where higher values (red) in the PAE heat map indicate regions where the model is less confident and the actual distance might be farther or closer than predicted. Molecular dynamics simulations for *ADRB2* WT and the Q27E polymorphism were performed on the active state structure bound to adrenaline (Protein Data Bank (PDB) 4LDO)^73^. Missing loops were not seen in the crystal density, and the N-terminal tail was added using MODELLER^74^. The molecular dynamics set-up was performed using the CHARMM-GUI molecular modelling suite^75^ following established protocols^76^. For the LEPR–Leptin dimer system, we performed coarse-grained molecular dynamics simulations and used as a starting structure the recently solved cryogenic electron microscopy structure (PDB 8DH8) by Saxton et al.^77^. Missing side chains were added with PyRosetta. Calculations of the fraction of native contacts and RMSF and RMSD calculations were performed using MDTraj^78^. Concurrence plot calculations were performed using Contact Map Explorer (https://contact-map.readthedocs.io/) with a 4.5 Å threshold. Contacts being present in more than 80% of simulation time were analysed.

### Human ganglia single-nuclei RNA-seq

Nuclei were isolated from human superior cervical and stellate ganglia as previously described^54,79^. The raw sequencing data were processed using Cell Ranger (v6.0.1, 10x Genomics) with intronic read inclusion, aligned to the GRCh38 v39 human reference genome (Genome Reference Consortium) and further corrected for technical artefacts using CellBender (https://github.com/broadinstitute/CellBender/). Downstream analysis, including quality control, data integration, dimensionality reduction and unsupervised clustering, was performed using Seurat (v5). Nuclei were excluded if they contained <500 or >2,000 detected genes, or if >1.0% of transcripts were mitochondrial. Sex assignment was based on genetic de-multiplexing and expression of sex-specific marker genes. A total of 11,228 nuclei were uniquely assigned, with 5,013 of these assigned to nuclei from female individuals and 6,215 to nuclei from male individuals. Plots were generated using SCpubr.

### Lentiviral overexpression of *LEPR* and *ADRB2* in HUVECs and drug treatments

Primary HUVECs (PromoCell, C-12200) were maintained in endothelial growth medium with supplement mix. The lentiviral vector system was used to transduce the cells with *LEPR* and *ADRB2*. VectorBuilder was used to design viral vectors. To check the transfection efficiency, a dual-reporter gene system was used. For instance, a drug-resistant gene (for example, puromycin or blasticidin) was used to select the cells transduced with the vector. A fluorescence tag (V5 or FLAG) was used to confirm the overexpression of our proteins of interest by immunofluorescence. WT HUVECS and LEPR^+^ADRB2^+^ HUVECS were treated with the following concentrations of drugs: low leptin (10 ng ml^−1^), high leptin (100 ng ml^−1^), high leptin (100 ng ml^−1^) + a sympathomimetic ADRB2 agonist (clenbuterol hydrochloride; 10 µg ml^−1^) for 24 h. After 24 h, cells were fixed with 4% PFA in PBS and processed for combined immunostaining and the Click-iT TUNEL assay.

### Statistics

Statistical analyses were performed with GraphPad (Prism 9.3.1) using an unpaired Student’s *t*-test (two-tailed) to compare two groups, one-way ANOVA with Turkey’s post hoc test to compare more than two groups and two-way ANOVA with Turkey’s post hoc test to compare two-factor study design. Metabolic parameters such as energy expenditure, VO_2_ and VCO_2_ were analysed with ANCOVA using body weight as a covariate. The data are shown as the mean ± s.e.m. unless stated otherwise. For all tests, *P* < 0.05 was considered significant. All data points represent individual biological samples. Data distribution was assumed to be normal, but this was not formally tested. All ex vivo and in situ experiments were performed at least two times. No statistical methods were used to predetermine sample size, but the sample size was similar to those generally used in the field^49,80^. Randomization was used whenever possible. Data collection and analysis were not performed blind to the conditions of the experiments.

### Reporting summary

Further information on research design is available in the Nature Portfolio Reporting Summary linked to this article.

## Supplementary information

Supplementary Information Supplementary Methods, Figs. 1–10 and legends, and Table legends 1–3.

Reporting Summary

Supplementary Table 1 Statistical analysis of indirect calorimetry data. a, Statistical analysis of metabolic cage data from chow-fed *Adrb2*^fl/fl^ and *Lepr*^Cre^: *Adrb2*^fl/fl^ mice. b, Statistical analysis of metabolic cage data from HFD-challenged *Adrb2*^fl/fl^ and *Lepr*^Cre^: *Adrb2*^fl/fl^ mice. Data were analysed by two-way ANOVA with Bonferroni post hoc test (RER and total activity) and ANCOVA (O_2_ consumption, CO_2_ production and energy expenditure).

Supplementary Table 2 The effects of *LEPR* and *ADRB2* variants in the regulation of BMI. a, *LEPR* variant Gln223Arg (rs1137101) and *ADRB2* variant Gln27Glu (rs1042714) effects on BMI in the UKB population. b, The independent effect of *LEPR* variant Gln223Arg or *ADRB2* variant Gln27Glu on BMI in men and women. c, The synergistic interaction between *LEPR* variant Gln223Arg and *ADRB2* variant Gln27Glu on BMI in men and women. EA, effect allele; EAF, effect allele frequency; AA, amino acid, Beta, effect size; SE, standard error; IAF, interaction allele frequency; *P* = *P* value. Data were analysed using linear regression with an interaction variable corrected for age, sex and the first ten principal components with Bonferroni correction for multiple testing. *P value < 0.05.

Supplementary Table 3 The list of primers used for RT–qPCR.

Supplementary Data 1 Source data for Supplementary Figs. 1, 2 and 4–9.

## Source data

Source Data Fig. 1 Statistical source data.

Source Data Fig. 2 Statistical source data.

Source Data Fig. 3 Statistical source data.

Source Data Fig. 4 Statistical source data.

Source Data Fig. 5 Statistical source data.

Source Data Fig. 6 Statistical source data.

Source Data Extended Data Fig./Table 1 Statistical source data.

Source Data Extended Data Fig./Table 2 Statistical source data.

Source Data Extended Data Fig./Table 5 Statistical source data.

Source Data Extended Data Fig./Table 6 Statistical source data.

Source Data Extended Data Fig./Table 7 Statistical source data.

Source Data Extended Data Fig./Table 8 Statistical source data.

Source Data Extended Data Fig./Table 10 Statistical source data.

## Data Availability

The raw single-cell sequencing data of sympathetic ganglia supporting the findings in this study have been deposited in the Gene Expression Omnibus (GEO) under accession code GSE233163. The raw data of the public dataset reanalysed in this study can be found in the GEO repository under the following accession codes: Tabula Muris (GSE109774 and GSE93374), mouse sympathetic ganglia (GSE231767), dorsal root ganglia (GSE175421), nodose ganglia (GSE124312), sciatic nerve (GSE137870), MCA (GSE108097), Tabula Muris Senis (GSE132042), WAT (mouse, GSE17617; human, GSE155960), BAT (mouse, GSE207707; human, E-MTAB-9199), human sympathetic ganglia (GSE241386) and Mouse Organogenesis Cell Atlas (GSE119945). The Seurat object containing the HypoMap dataset is available via 10.17863/CAM.87955. Source data are provided with this paper.

## Code availability

No custom code or mathematical algorithm was created in this work. The code used to create figures from scRNA-seq dataset and all additional input data files can be found on GitHub via https://github.com/automet26/Sympathetic_Perineurium_Leptin_Obesity. Please direct any other bioinformatic inquiries to G.S.
