# Author Correction: The perineurium integrates leptin with its sympathetic outflow to protect against obesity

**DOI:** 10.1038/s42255-026-01605-w

**Published:** 2026-08-11

**Authors:** Gitalee Sarker, Emma Haberman, Anandhakumar Chandran, Thomas Monfeuga, Cyrielle Maroteau, Sofia Lundh, David S. Oliveira, Andrea Raimondi, Karin Ziegler, Tiago Gomes, Tiago N. Cordeiro, Karishma Parekh, Emilia Schmid, Paola Fernández-Sanmartín, Alina Griebel, Noelia Martinez-Sanchez, Bernardo A. Arús, Samson W. Cheung, Yitao Zhu, Michal Shaked, Sarah C. L. Knowles, Stefan Hanns Engelhardt, Matteo Iannacone, Philipp E. Scherer, Miguel López, Enrique M. Toledo, Ana I. Domingos

**Affiliations:** 1 Department of Physiology, Anatomy and Genetics, University of Oxford, Oxford, UK; 2 Research and Development, Novo Nordisk Research Centre Oxford, Oxford, UK; 3 Department of Pathology and Imaging, Global Drug Discovery, Novo Nordisk, Måløv, Denmark; 4 Experimental Imaging Centre, IRCCS San Raffaele Scientific Institute, Milan, Italy; 5 Institute of Pharmacology and Toxicology, Technical University Munich (TUM), Munich, Germany; 6 DZHK (German Centre for Cardiovascular Research), Partner Site Munich Heart Alliance, Munich, Germany; 7 Instituto de Tecnologia Química e Biológica António Xavier, ITQB NOVA, Lisbon, Portugal; 8 Neurobesity Group, Department of Physiology, Center for Research in Molecular Medicine and Chronic Diseases (CiMUS), Universidade de Santiago de Compostela, Santiago, Spain; 9 Oxford Centre for Diabetes, Endocrinology and Metabolism, Oxford, UK; 10 Helmholtz-Zentrum Dresden-Rossendorf (HZDR), Dresden, Germany; 11 Medizinische Fakultät and University Hospital Carl Gustav Carus, Technische Universität Dresden, Dresden, Germany; 12 Department of Functional Imaging in Surgical Oncology, National Center for Tumor Diseases (NCT/UCC), Dresden, Germany; 13 German Cancer Research Center (DKFZ), Heidelberg, Germany; 14 Department of Biology, University of Oxford, Oxford, UK; 15 Vita-Salute San Raffaele University, Milan, Italy; 16 Division of Immunology, Transplantation and Infectious Diseases, IRCCS San Raffaele Scientific Institute, Milan, Italy; 17 Touchstone Diabetes Center, The University of Texas Southwestern Medical Center, Dallas, TX USA

**Keywords:** Molecular biology, Neuroscience, Metabolism

Correction to: *Nature Metabolism* https://doi.org/10.1038/s42255-026-01555-3, published online 13 July 2026.

In the version of the article initially published, Ana I. Domingos’s name was listed without a middle initial. This has now been corrected in the HTML and PDF versions of the article.

