# Supplementary material for: The perineurium integrates leptin with its sympathetic outflow to protect against obesity
Source: Nat Metab. 2026 Jul 13;8(7):1563–82. doi: 10.1038/s42255-026-01555-3 (PMC13400315; doi:10.1038/s42255-026-01555-3)
Supplement: Supplementary file 1 — Supplementary Methods, Figs. 1–10 and legends, and Table legends 1–3. [file 42255_2026_1555_MOESM1_ESM.pdf]

nature metabolism

Supplementary information https://doi.org/10.1038/s42255-026-01555-3

The perineurium integrates leptin with its sympathetic outflow to protect against obesity

In the format provided by the authors and unedited

Supplementary Figures with legends (1-10)

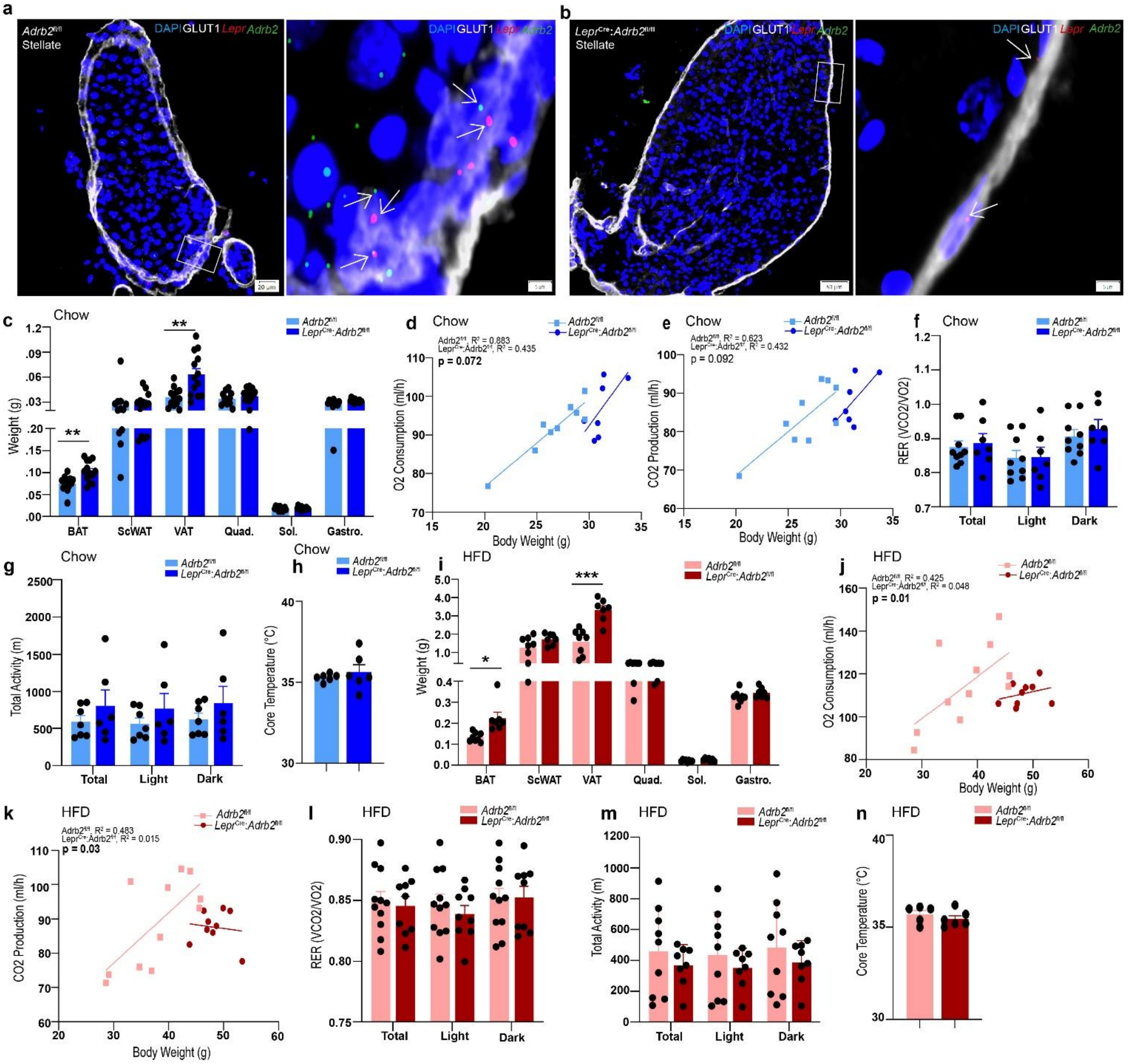

Supplementary Fig. 1. Impaired metabolic homeostasis in *Lepr*^{Cre}: *Adrb2*$^{fl/fl}$ mice. a-b, Higher magnification image showing the absence of *Lepr* and *Adrb2* mRNA colocalization in the perineurium of stellate ganglia of *Lepr*^{Cre}: *Adrb2*$^{fl/fl}$ mice. The white box in the left-panel images indicates the field of view of the higher-resolution images in the right panel. **c,** Weight of individual fat and muscles measured in 14-week-old chow-fed *Lepr*^{Cre}: *Adrb2*$^{fl/fl}$ (n = 13) and *Adrb2*$^{fl/fl}$ mice (n = 12). Individual data points representing biological replicates. p = BAT (0.0034), VAT (0.0031). **d-e**, Regression plots of average daily O_2 consumption **(d)**, and CO_2 production **(e)** as a function of body weight (n = 9-7/ group). **f-g,** Average measurements of respiratory exchange ratio (RER) **(f)** *Lepr*^{Cre}: *Adrb2*$^{fl/fl}$ (n = 7) and *Adrb2*$^{fl/fl}$ mice (n = 9) and activity **(g)** *Lepr*^{Cre}: *Adrb2*$^{fl/fl}$ (n = 6) and *Adrb2*$^{fl/fl}$ mice (n = 7) across the full day or 12 h dark and light cycles. Individual data points representing biological replicates. **h**, Core body temperature measured in 18-week-old chow-fed mice. n = 6 per group, with individual data points representing biological replicates. **i**, Weight of individual fat and muscles measured in 10 weeks of high-fat diet (HFD) fed *Lepr*^{Cre}: *Adrb2*$^{fl/fl}$ (n = 7) and *Adrb2*$^{fl/fl}$ mice (n = 8). Individual data points representing biological replicates. p = BAT (0.015), VAT (0.0002). **j-k**, Regression plots of average daily O_2 consumption **(j)**, and CO_2 production **(k)** as a function of body weight (n = 9-11/ group). **l-m,** Average measurements of respiratory exchange ratio (RER) **(l)** and activity **(m)** across the full day or 12 h dark and light cycles. n = 6 per group, with individual data points representing biological replicates. **n**, Core body temperature measured in 12-week HFD-fed mice. n = 5/6 per group, with individual data points representing biological replicates. Data were mean ± s.e.m and were analyzed by two-way ANOVA with Bonferroni post-hoc test (**c, f, g, i, l, m**), ANCOVA (**d, e, j, k**), and two-tailed unpaired Student's t-test (**h, n**). $^*p < 0.05$, $^{**}p < 0.01$, $^{***}p < 0.001$. Quad. = quadriceps,

Sol. = soleus, Gastro. = gastrocnemius, scWAT = subcutaneous white adipose tissue, BAT = brown adipose tissue, HFD = high fat diet.

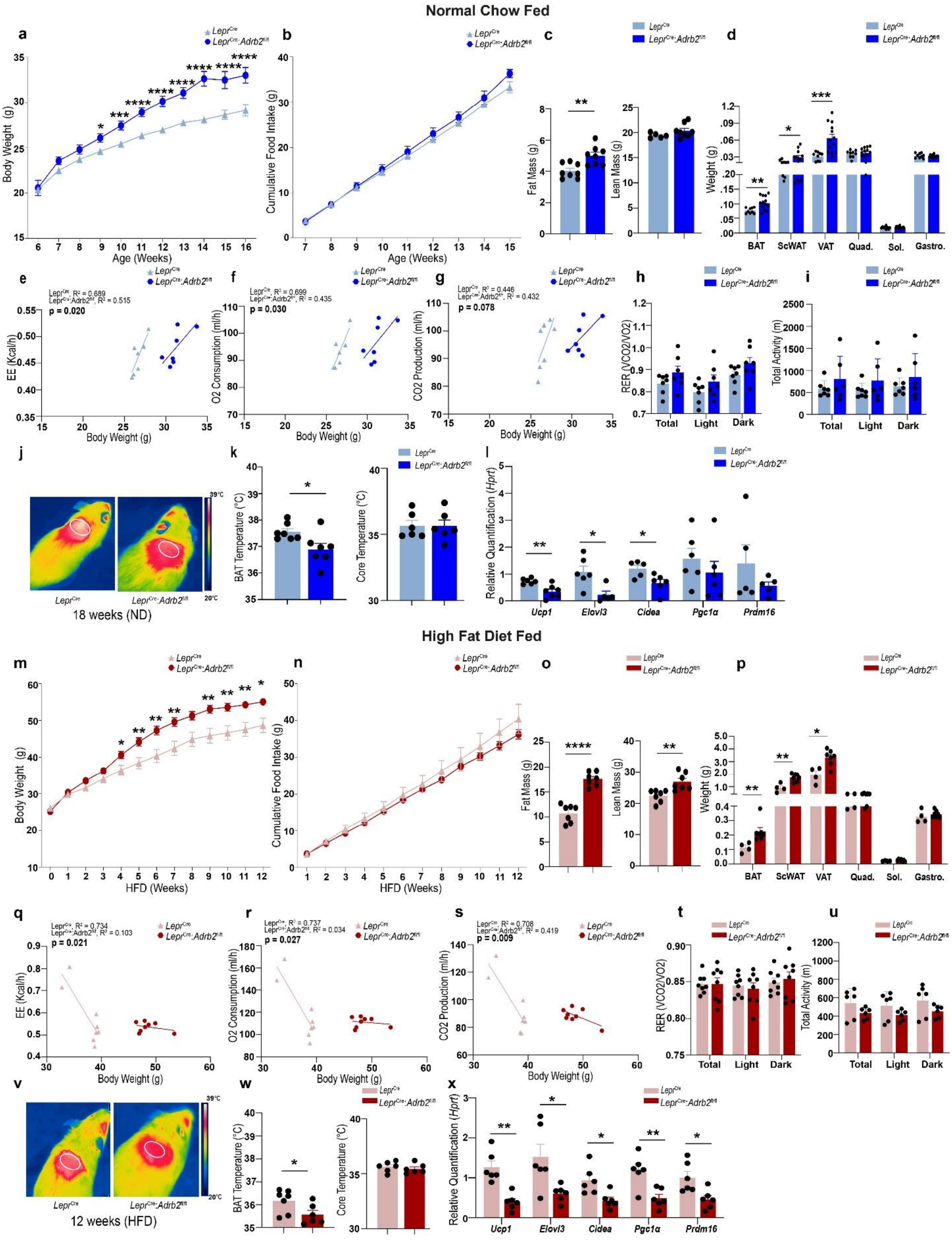

Supplementary Fig. 2. ***Lepr*-driven Cre expression does not mediate obesogenic phenotype in *Lepr*^{Cre}; *Adrb2*$^{\mathrm{fl/fl}}$ mice. a,** Body weight measured in chow fed *Lepr*^{Cre}; *Adrb2*$^{\mathrm{fl/fl}}$ (n = 19) and *Lepr*^{Cre} (n = 14) mice. p values by week: 6 (0.846), 7

(0.0568), 8 (0.0619), 9 (0.0098), 10 (0.0012), 11 (0.0001), 12 (<0.0001), 13 (0.0001), 14 (0.0002), 15 (0.0026), 16 (0.0021). **b,** Cumulative food intake, *Lepr*^{Cre}; *Adrb2*$^{fl/fl}$ (n = 19) and *Lepr*^{Cre} (n = 14). **c,** Body composition measured in 14-week-old mice. n = 8 per group with individual data points representing biological replicates. p = 0.003. **d**, Individual fat and muscle mass measured in 14-week-old *Lepr*^{Cre}: *Adrb2*$^{fl/fl}$ (n = 13) and *Lepr*^{Cre}mice (n = 8). Individual data points representing biological replicates. p = BAT (0.003), scWAT (0.03), VAT (0.001). **e-g**, Regression plots of average daily energy consumption, O_2 consumption, and CO_2 production as a function of body weight (n = 7/ group). **h,** Average respiratory exchange ratio (RER). n = 7 per group with individual data points representing biological replicates. **i,** Total activity across the full day or 12 h dark and light cycles. *Lepr*^{Cre}: *Adrb2*$^{fl/fl}$ (n = 6) and *Lepr*^{Cre} mice (n = 7). Individual data points representing biological replicates. **j**, Representative thermal imaging of the BAT of 18-week-old mice. **k**, BAT and core body temperature measurement (n = 7/group). p = 0.02. **l**, Thermogenic gene expression in BAT. *Lepr*^{Cre}: *Adrb2*$^{fl/fl}$ (n = 5/7) and *Lepr*^{Cre} (n = 5/6). p values: *Ucp1* (0.004), *Elovl3* (0.01), *Cidea* (0.02), Pgc1α (0.38), *Prdm16* (0.34). **m,** Body weight measured in 12-week HFD fed *Lepr*^{Cre}; *Adrb2*$^{fl/fl}$ (n = 17) and *Lepr*^{Cre} (n = 11) mice. p values by week: 0 (0.1711), 1 (0.6275), 2 (0.1327), 3 (0.1389), 4 (0.0223), 5 (0.0063), 6 (0.0043), 7 (0.0059), 8 (0.0121), 9 (0.0063), 10 (0.0069), 11 (0.0065), 12 (0.01). **n,** Cumulative food intake. *Lepr*^{Cre}; *Adrb2*$^{fl/fl}$ (n = 17) and *Lepr*^{Cre} (n = 11) mice. **o,** Body composition measured in 10-week-HFD-fed mice. n = 7 per group with individual data points representing biological replicates. p = fat (< 0.0001), lean (0.004). **p**, Individual fat and muscle mass measured in 10-week-HFD-fed mice. *Lepr*^{Cre}: *Adrb2*$^{fl/fl}$ (n = 7) and *Lepr*^{Cre} (n = 4). Individual data points representing biological replicates. p = BAT (0.007), scWAT (0.008), VAT (0.012). **q-s**, Regression plots of average daily energy consumption, O_2 consumption, and CO_2 production as a function of body weight (n = 8/ group). **t,** Average respiratory exchange ratio (RER). n = 8 per group with individual data points representing biological replicates. **u,** Total activity across the full day or 12 h dark and light cycles. n = 6 per group with individual data points representing biological replicates. **v**, Representative thermal imaging of the BAT of 12-week-HFD fed mice. **w**, BAT (*Lepr*^{Cre}: *Adrb2*$^{fl/fl}$ (n = 6) and *Lepr*^{Cre} (n = 7)) and core body (6 mice per group) temperature measurement. p = 0.04. **x**, Thermogenic gene expression in BAT (n = 6/group). p values: *Ucp1* (0.001), *Elovl3* (0.03), *Cidea* (0.01), Pgc1α (0.006), *Prdm16* (0.015). Data were mean ± s.e.m. and were analyzed by two-way ANOVA with Bonferroni post-hoc test (**a, b, d, h, i, m, n, p, t, u**), ANCOVA (**e, f, g, q, r, s**), and two-tailed unpaired Student's t-test (c, **k, l, o, w, x**). *p < 0.05, **p < 0.01, ***p < 0.001. Quad. = quadriceps, Sol. = soleus, Gastro. = gastrocnemius, scWAT = subcutaneous white adipose tissue, BAT = brown adipose tissue.

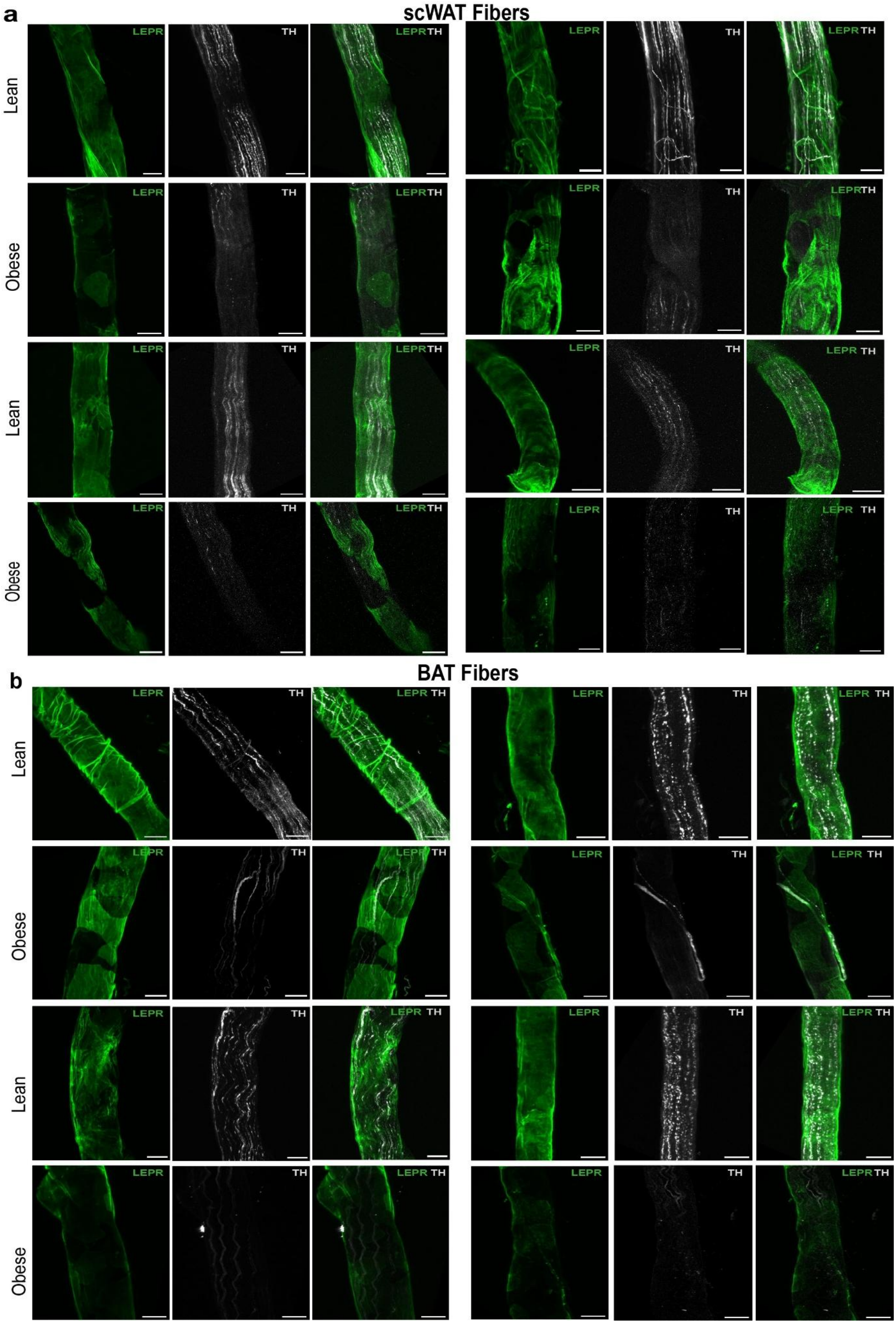
a
scWAT Fibers
Lean
Obese
Lean
Obese
LEPR
TH
LEPR TH
b
BAT Fibers
Lean
Obese
Lean
Obese
LEPR
TH
LEPR TH

Supplementary Fig. 3. Diet-induced obesity destroys the perineurial barrier and reduces sympathetic neurons. a, Representative images of scWAT bundles from ND-treated lean and HFD-treated obese (*Lepr*^{Cre}; LSL-YFP) reporter mice showing the expression of LEPR (YFP) and TH. **b**, Representative images of BAT bundles from ND-treated lean and HFD-treated obese (*Lepr*^{Cre}; LSL-YFP) reporter mice showing the expression of LEPR (YFP) and TH. Scale bar: 50µm. The experiments were repeated three times with similar results.

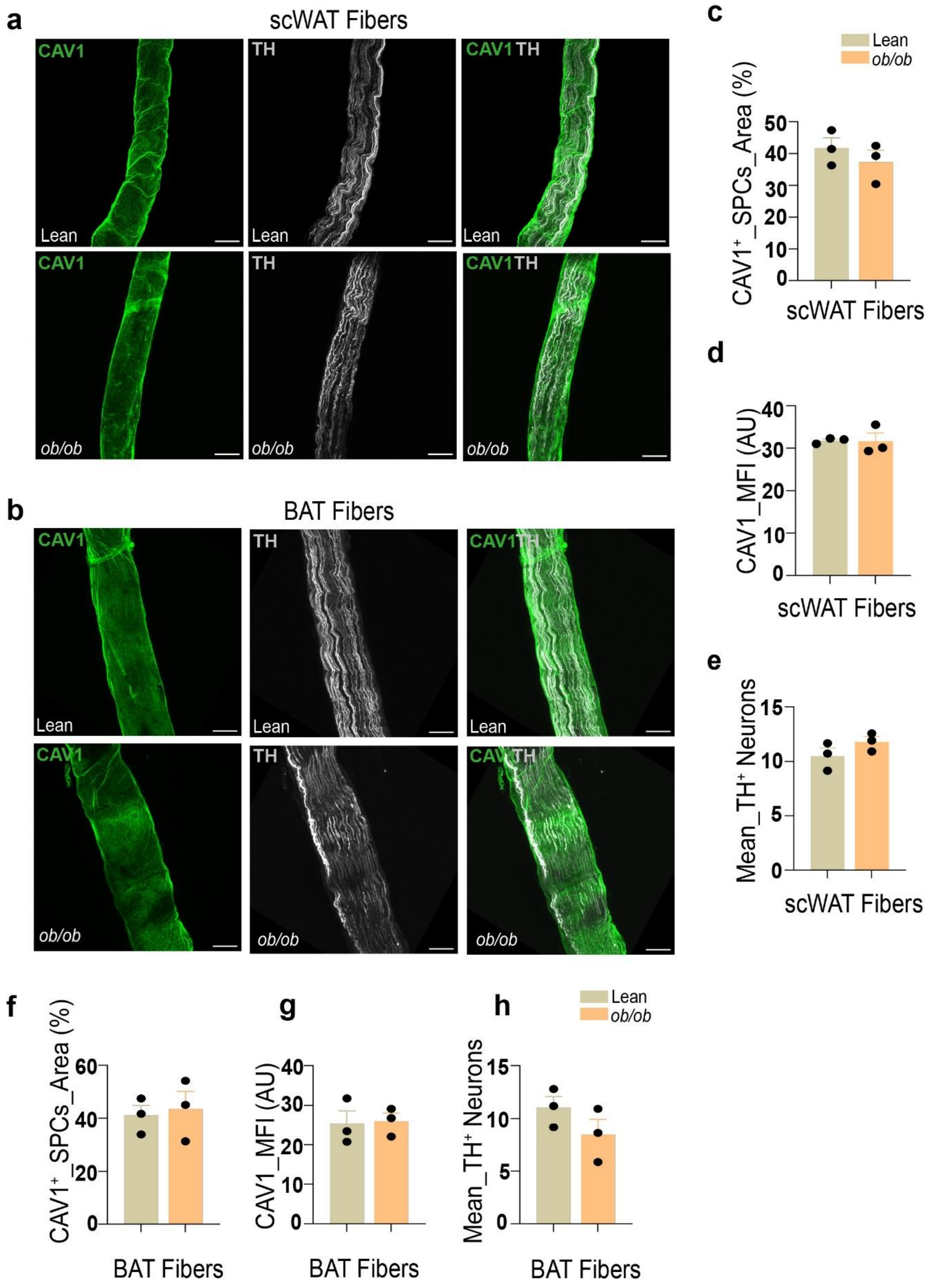

Supplementary Fig. 4. SPCs barrier remains unaltered in leptin-deficient *ob/ob* mice. a-b, Representative images of scWAT (**a**) and BAT (**b**) fibers from lean wild type and *ob*/*ob* mice showing the expression of CAV1 and TH. **c-e,** Image quantification of CAV1$^+$ SPCs area, CAV1 intensity, and TH$^+$ neurons in lean and *ob*/*ob* scWAT fibers (n = 3 mice per group, with individual data points representing biological replicates). **f-h,** Quantification of CAV1$^+$ SPCs area, CAV1 intensity, and TH$^+$ sympathetic neurons in lean and *ob*/*ob* BAT fibers (n = 3 mice per group, with individual data points representing biological replicates). Scale bar: 50 µm. Data were analysed by two-tailed unpaired Student's t-test and are shown as mean ± s.e.m.

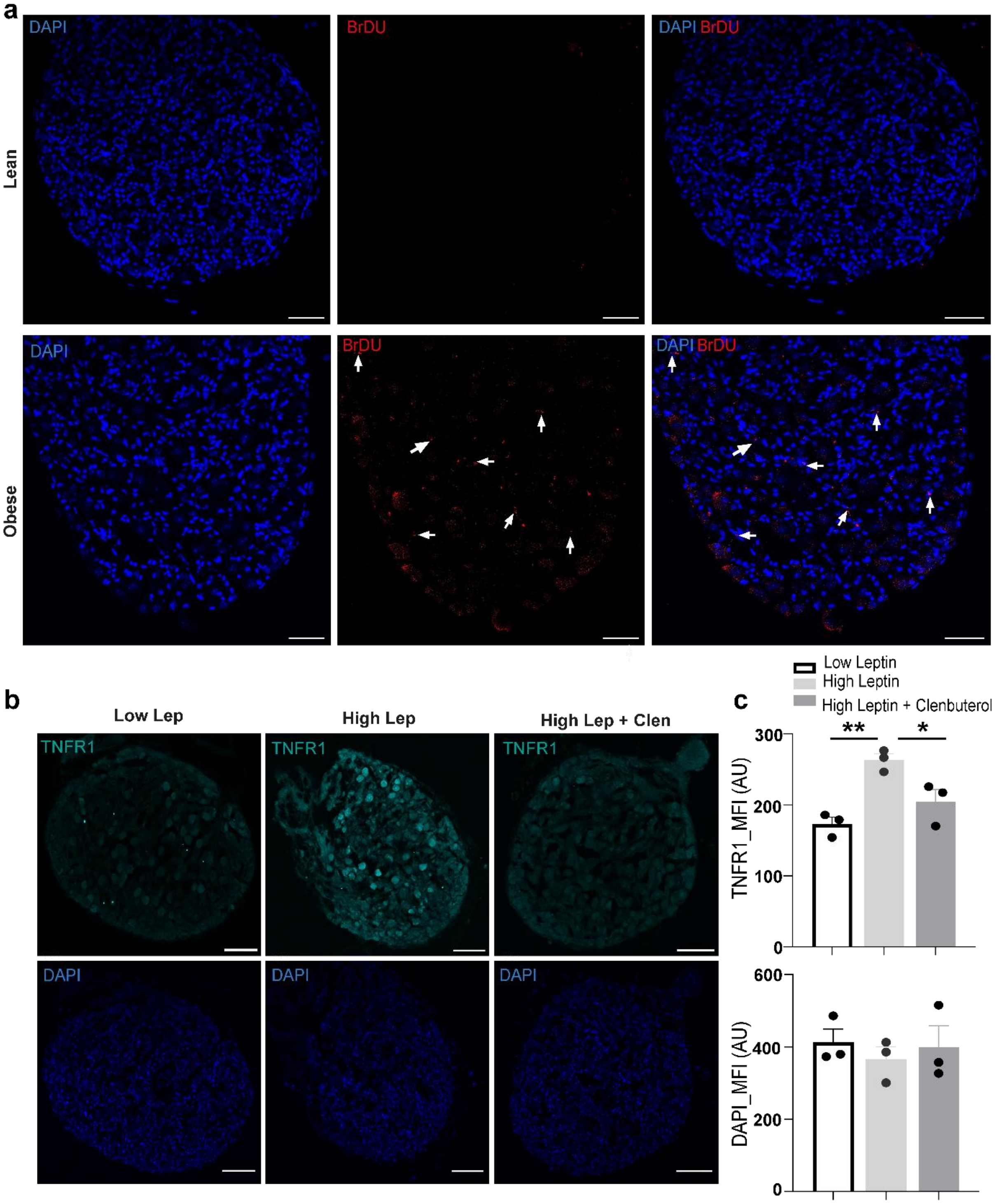

Supplementary Fig. 5. Hyperleptinemia-induced apoptosis is reversed by β2 adrenergic agonism in SCG explant. a, TUNEL assay on SCG sections of lean and diet-induced obese mice. TUNEL-positive cells show a red dot if degraded DNA

is detected, while DAPI staining displays the blue nucleus. Scale bar: 50µm. **b,** Representative images of SCG explant culture showing the expression of TNFR1 and DAPI following low leptin (10 ng/ml), high leptin (100 ng/ml), and high leptin (100 ng/ml) plus clenbuterol (10 µg/ml). Scale bar: 100 µm. **c**, Quantification of the expression of TNFR1 and DAPI in SCG explant following different stimulating conditions. ($n = 3$ per condition). $p = 0.002$ (low lep vs high lep), 0.04 (high lep vs high lep + clen). MFI = Mean Fluorescence Intensity. Data are mean ± s.e.m and were analyzed using one-way ANOVA with Turkey's multiple comparison test. *$p < 0.05$, **$p < 0.01$.

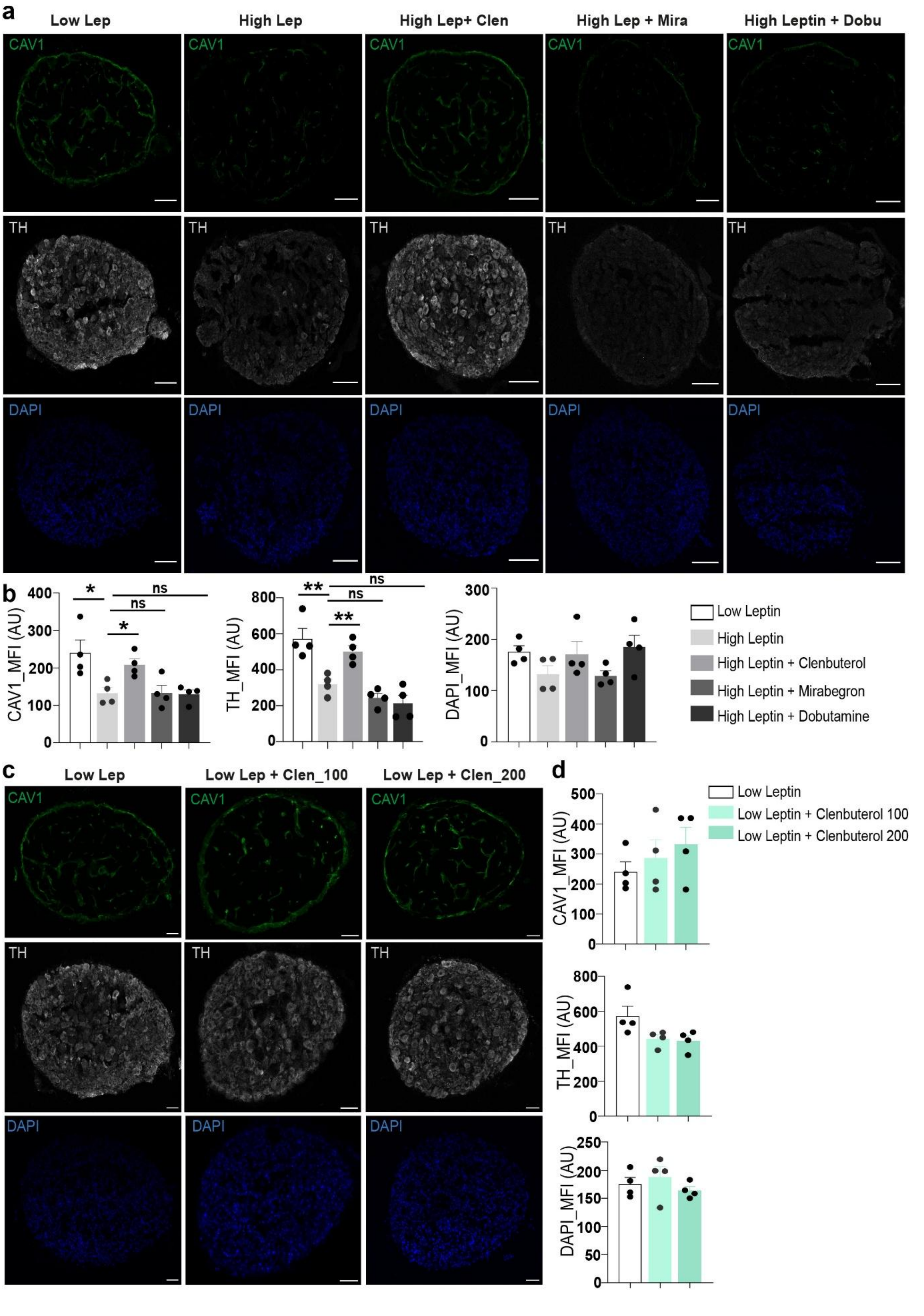

Supplementary Fig. 6. Obesity-driven hyperleptinemia causes SPCs loss, reversible exclusively by β2 adrenergic agonists and unaffected by disproportionately high adrenergic signalling. **a**, Representative images of SCG explant showing the expression of CAV1, TH, and DAPI following low leptin (10 ng/ml), high leptin (100 ng/ml), high leptin (100

ng/ml) plus Clenbuterol (10 μg/ml), high leptin (100 ng/ml) plus Mirabegron (10μM) and high leptin (100 ng/ml) plus Dobutamine (50μM) treatment. Scale bar, 100 μm. **b**, Quantification of CAV1, TH, and DAPI expression in SCG explant following different stimulating conditions (n = 4 mice per condition). CAV1_MFI; p = 0.03 (low lep vs high lep), 0.01 (high lep vs high lep + clen). TH_MFI; p = 0.008 (low lep vs high lep), 0.006 (high lep vs high lep + clen).**c**, Representative images of SCG explant showing the expression of CAV1, TH, and DAPI following low leptin (10 ng/ml), low leptin (10 ng/ml) + high Clenbuterol (100 μg/ml), and low leptin (10 ng/ml) + high Clenbuterol (200 μg/ml) treatment. Scale bar, 50 μm. **d**, Quantification of CAV1, TH, and DAPI expression in SCG explant following different stimulating conditions. (n = 4 mice per condition). Individual data points representing biological replicates. MFI = Mean Fluorescence Intensity, Lep = leptin, Clen = Clenbuterol, Mira = Mirabegron, Dobu = Dobutamine. Data are mean ± s.e.m. and were analyzed using one-way ANOVA with Turkey's Multiple Comparison test. *p < 0.05, **p < 0.01, ***p < 0.001.

.

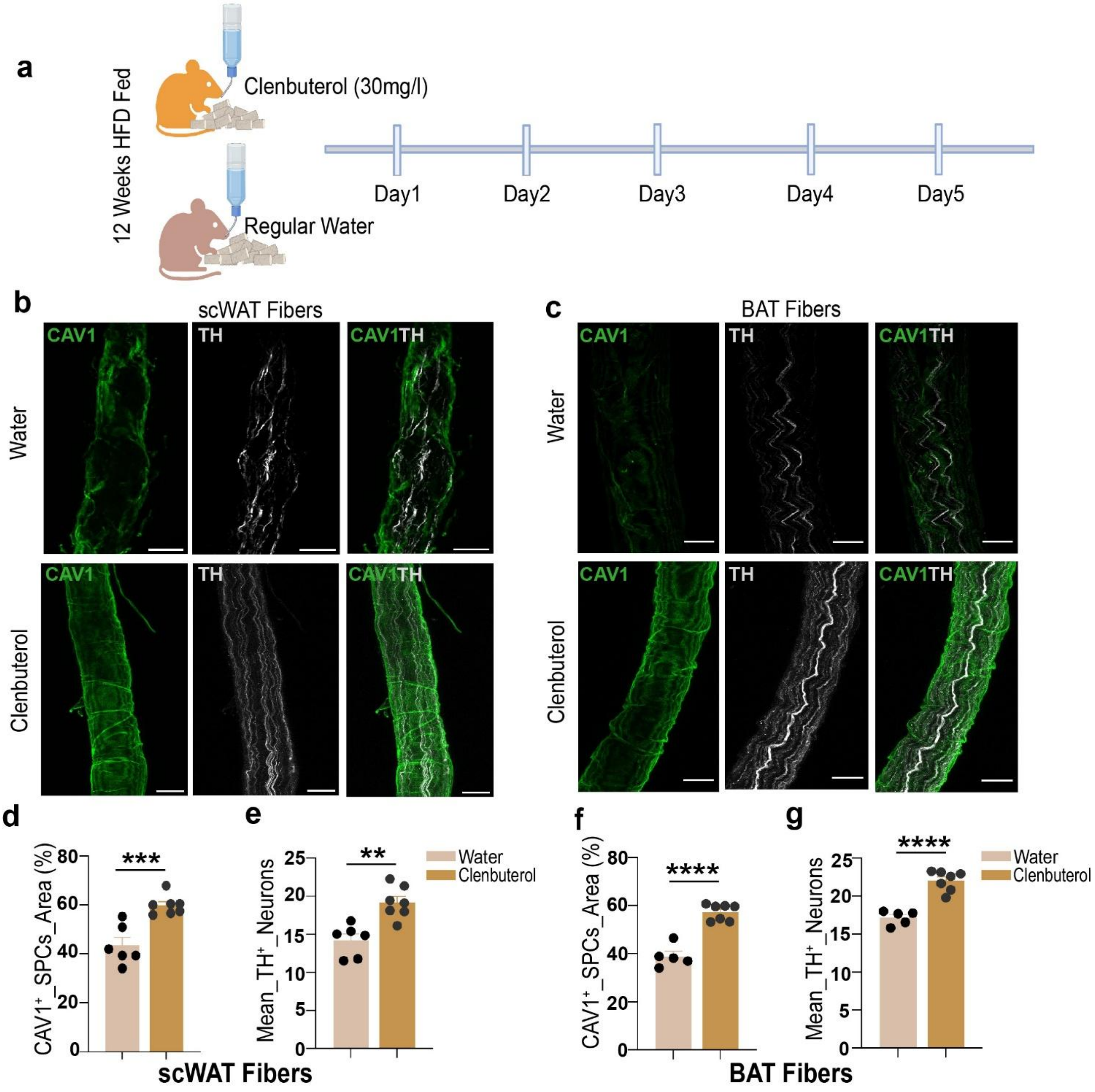

Supplementary Fig. 7. Obesity-driven hyperleptinemia causes loss of the SPCs, which is rescued *in vivo* by ADRB2 agonism. a, Scheme of HFD-induced obese (DIO) mice consumed Clenbuterol (30mg/l) via drinking water or had regular drinking water for 5 consecutive days. *Created in BioRender. Sarker, G. (2026) https://BioRender.com/ms2jk9h*. **b**, Representative images of scWAT bundles from DIO mice consumed Clenbuterol water and regular water, showing the expression of CAV1 and TH. **c**, Representative images of BAT bundles from DIO mice consumed Clenbuterol water and regular drinking water, showing the expression of CAV1 and TH. Scale bar: 50µm. **d-e**, Quantification of $CAV1^+$ SPCs barrier ($p = 0.0006$) and TH^+ sympathetic neurons ($p = 0.0013$) in scWAT fibers isolated from DIO mice consumed Clenbuterol water and regular water (n = 6 (water), 7 (clenbuterol)). **f-g**, Quantification of $CAV1^+$ SPCs barrier ($p < 0.0001$) and TH^+ sympathetic neurons ($p < 0.0001$) in BAT fibers isolated from DIO mice consumed Clenbuterol water and regular water (n = water (5), Clenbuterol (7)). Data were analyzed by two-tailed unpaired Student's t-test and were shown as mean ± s.e.m (e-h). **p < 0.01, ***p<.001, ****p<.0001.

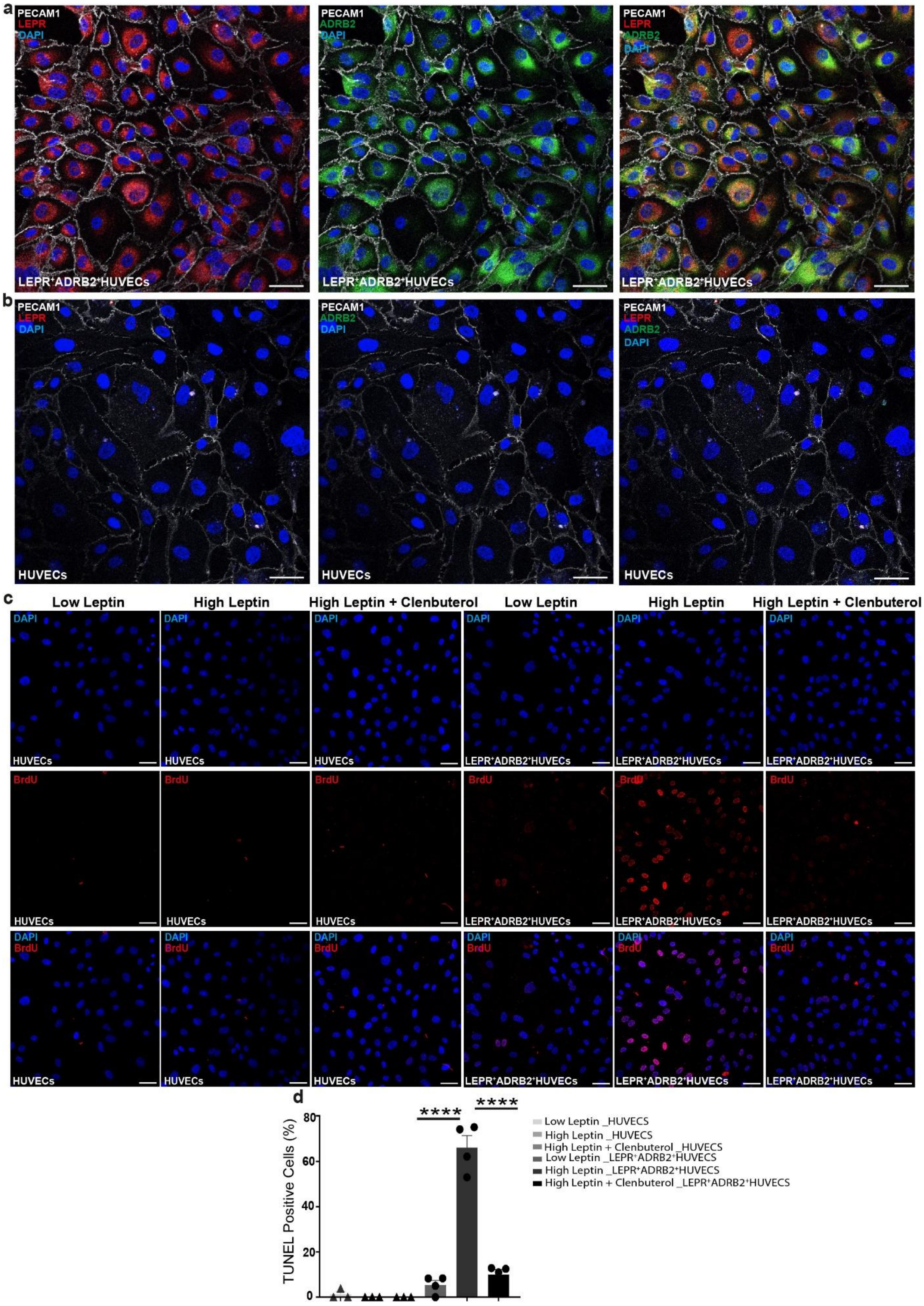
a
PECAM1
LEPR
DAPI
ADRB2
LEPR+ADRB2+HUVECs
b
HUVECs
c
Low Leptin
High Leptin
High Leptin + Clenbuterol
BrdU
d
TUNEL Positive Cells (%)
****
Low Leptin _HUVECS
High Leptin _HUVECS
High Leptin + Clenbuterol _HUVECS
Low Leptin _LEPR+ADRB2+HUVECS
High Leptin _LEPR+ADRB2+HUVECS
High Leptin + Clenbuterol _LEPR+ADRB2+HUVECS

Supplementary Fig. 8. β2 adrenergic signaling counteracts leptin-induced apoptosis in human endothelial cells overexpressing LEPR and ADRB2. **a, b** Representative images of HUVECs overexpressing LEPR and ADRB2 and wild-type HUVECs showing the expression of PECAM1, LEPR, ADRB2, and DAPI. Scale bar: 50µm. **c,** TUNEL staining reveals apoptosis of LEPR^{+}ADRB2^{+}HVECS treated with high leptin, which is rescued by clenbuterol. No apoptosis is detected in wild-type HUVECs. TUNEL-positive cells are marked as red if degraded DNA is detected, while DAPI staining displays the blue nucleus. Scale bar: 50µm. **d**, Quantification of TUNEL-positive cells following different stimulating conditions. n = 3/4 with individual data points representing technical replicates. Data are mean ± s.e.m. and were analyzed using one-way ANOVA with Turkey's Multiple Comparison test. ****p < 0.0001.

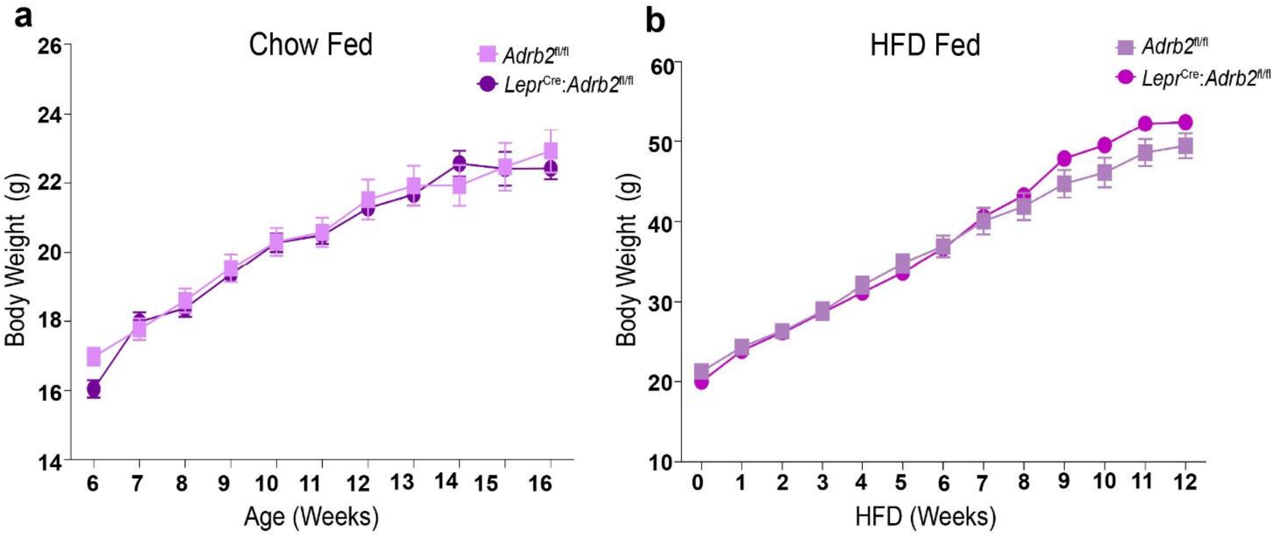

Supplementary Fig. 9. Chow-fed and high-fat diet-fed female *Lepr*^{Cre}: *Adrb2*$^{fl/fl}$ did not show any body weight difference compared to *Adrb2*$^{fl/fl}$ mice. a, Body weight of chow-fed mice from Week 6 to Week 16. n = (*Adrb2*$^{fl/fl}$, 22), (*Lepr*^{Cre}: *Adrb2*$^{fl/fl}$, 22). **b**, Body weight of *Lepr*^{Cre}: *Adrb2*$^{fl/fl}$ and *Adrb2*$^{fl/fl}$ mice challenged with 12 weeks of high-fat diet (HFD). n = (*Adrb2*$^{fl/fl}$, 8), (*Lepr*^{Cre}: *Adrb2*$^{fl/fl}$, 8). Data were mean ± s.e.m. and were analyzed using two-way ANOVA with Bonferroni post-hoc test.

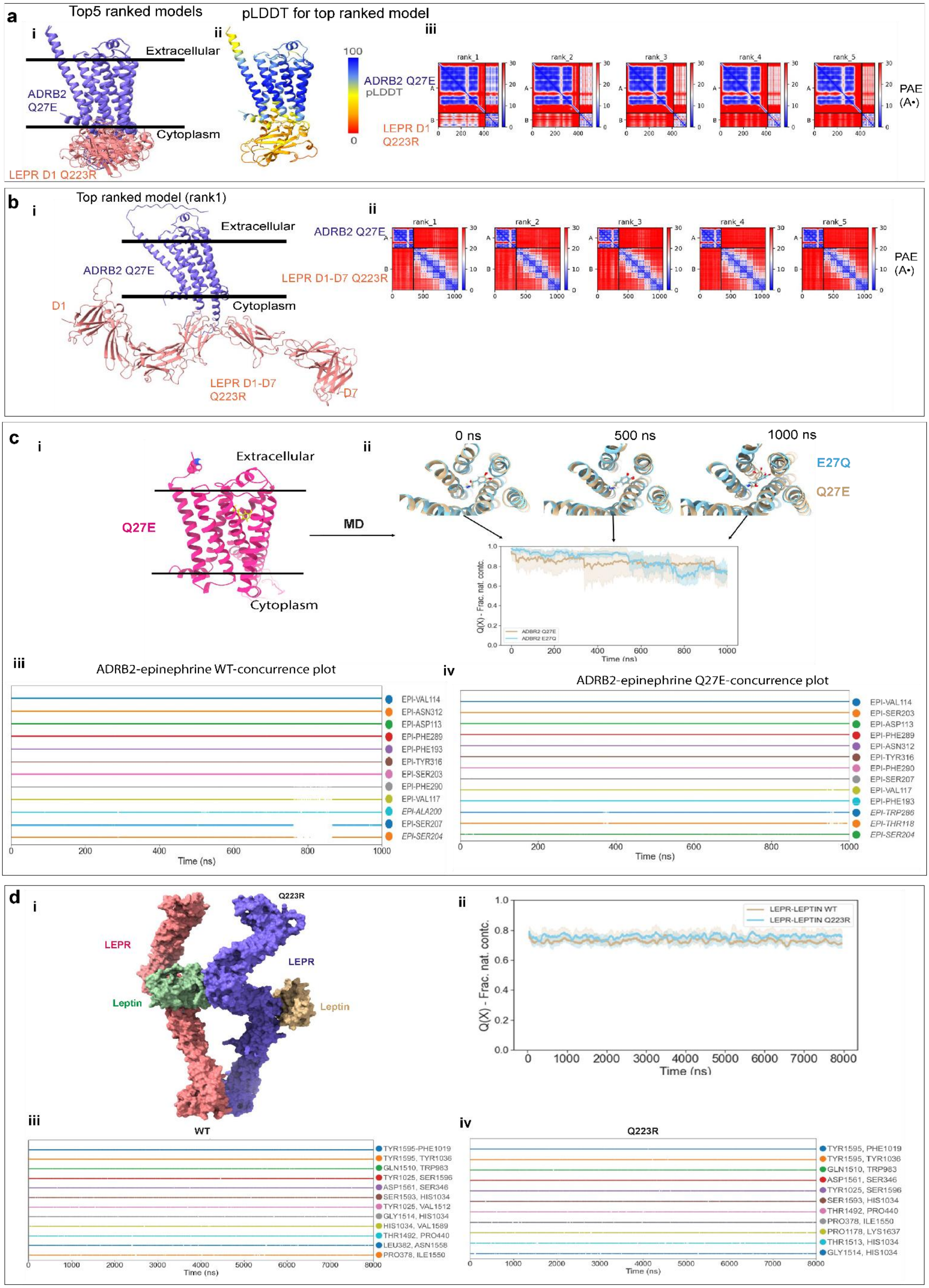

a
Top5 ranked models
pLDDT for top ranked model
i
ii
iii
Extracellular
ADRB2 Q27E
Cytoplasm
LEPR D1 Q223R
100
0
ADRB2 Q27E pLDDT
LEPR D1 Q223R
rank_1
rank_2
rank_3
rank_4
rank_5
PAE (A•)
b
Top ranked model (rank1)
Extracellular
ADRB2 Q27E
Cytoplasm
D1
LEPR D1-D7 Q223R
D7
ADRB2 Q27E
LEPR D1-D7 Q223R
PAE (A•)
c
Extracellular
Q27E
MD
Cytoplasm
0 ns
500 ns
1000 ns
E27Q
Q27E
Q(X) - Frac. nat. contc.
ADBR2 Q27E
ADBR2 E27Q
Time (ns)
ADRB2-epinephrine WT-concurrence plot
EPI-VAL114
EPI-ASN312
EPI-ASP113
EPI-PHE289
EPI-PHE193
EPI-TYR316
EPI-SER203
EPI-PHE290
EPI-VAL117
EPI-ALA200
EPI-SER207
EPI-SER204
Time (ns)
ADRB2-epinephrine Q27E-concurrence plot
EPI-VAL114
EPI-SER203
EPI-ASP113
EPI-PHE289
EPI-ASN312
EPI-TYR316
EPI-PHE290
EPI-SER207
EPI-VAL117
EPI-PHE193
EPI-TRP286
EPI-THR118
EPI-SER204
Time (ns)
d
Q223R
LEPR
LEPR
Leptin
Leptin
LEPR-LEPTIN WT
LEPR-LEPTIN Q223R
Q(X) - Frac. nat. contc.
Time (ns)
WT
TYR1595-PHE1019
TYR1595, TYR1036
GLN1510, TRP983
TYR1025, SER1596
ASP1561, SER346
SER1593, HIS1034
TYR1025, VAL1512
GLY1514, HIS1034
HIS1034, VAL1589
THR1492, PRO440
LEU382, ASN1558
PRO378, ILE1550
Time (ns)
Q223R
TYR1595, PHE1019
TYR1595, TYR1036
GLN1510, TRP983
ASP1561, SER346
TYR1025, SER1596
SER1593, HIS1034
THR1492, PRO440
PRO378, ILE1550
PRO1178, LYS1637
THR1513, HIS1034
GLY1514, HIS1034
Time (ns)

Supplementary Fig. 10. AlphaFold2 model of human β2 adrenergic (ADRB2) and leptin receptor (LEPR) and molecular dynamic simulation of ADRB2 and LEPR binding to ligands. a, AF2 model of Q27E ADRB2-Q223R LEPR D1 interaction: i, AF2 top ranked models. In purple is represented the ADRB2 Q27E polymorphism and in coral the LEPR Q223R polymorphism. ii, Top-ranked model from panel i with the pLDDT metric in a color gradient from blue (100) to red (0). iii, PAE plot for the top five models. b, AF2 model of ADRB2 Q27E - LEPR Q223R D1-D7 interaction: i, Top ranked model retrieved from the AF2 prediction. In purple is represented the ADRB2 Q27E and in coral the LEPR D1-D7 Q223R polymorphisms. ii, PAE plot for the top five models. **c,** Molecular dynamics simulations of the ADRB2-epinephrine complex: i, the ADRB2 receptor is depicted in pink, the epinephrine ligand is in yellow sticks and the Q27E mutation in blue and pink sphere. ii, Native contacts analysis for the full 1000 ns MD simulation. In brown and blue are represented the Q27E and WT polymorphisms, respectively. In darker color is depicted the average of three replicas and in shaded color the standard deviation. Also in cartoon representation, for the receptor, and stick representation, for the ligand, are depicted as aligned representative frames at 0, 500, and 1000 ns with the color code indicated above. iii, Concurrence plot for the ADRB2-epinephrine WT simulation with contacting residues indicated in the right panel legend. In italics are the residues showing contacts not seen in the crystal structure (PDB:4LDO), with the non-italic counterpart representing contacts seen in the same crystal structure. iv, Concurrence plot for the ADRB2-epinephrine Q27E simulation with contacting residues indicated in the legend. **d,** Molecular dynamics simulations of LEPR receptor - Leptin for WT and the Q223R polymorphism: i, In surface representation (pink and violet) is depicted the full-length LEPR dimer (domains D1-D7). Leptin is represented as green and yellow. The Q223R black label indicates where this polymorphism is located in the LEPR receptor full-length structure. ii, Native contacts analysis for the full 8000 ns MD simulation between LEPR and Leptin. In brown and blue are represented the WT and Q223R polymorphism, respectively. iii, Concurrence plots for the residues of the LEPR contacting Leptin for the 8000 ns simulation for replica 1 for the WT protein. Iv, Concurrence plot for the Q223R polymorphism with the same numbering as for panel iii

Supplementary Table Legends

Supplementary Table 1|Statistical analysis of indirect calorimetry data. a, Statistical analysis of metabolic cage data from chow-fed *Adrb2*$^{fl/fl}$ and *Lepr*^{Cre}: *Adrb2*$^{fl/fl}$ mice. **b**, Statistical analysis of metabolic cage data from high-fat diet (HFD) challenged *Adrb2*$^{fl/fl}$ and *Lepr*^{Cre}: *Adrb2*$^{fl/fl}$ mice. Data were analyzed by two-way ANOVA with Bonferroni post-hoc test **(**RER and total activity) and ANCOVA (O2 consumption, CO2 production, and EE).

Supplementary Table 2|The effects of *LEPR* and *ADRB2* variants in the regulation of BMI.

***a**, LEPR* variant Gln223Arg (rs1137101) and *ADRB2* variant Gln27Glu (rs1042714) effects on BMI in all UKB population. **b**, The independent effect of *LEPR* variant Gln223Arg or *ADRB2* variant Gln27Glu on BMI in men and women. **c**, The synergistic interaction between *LEPR* variant Gln223Arg and *ADRB2* variant Gln27Glu on BMI in men and women. EA = Effect Allele, EAF = Effect Allele Frequency, AA = Amino Acid, Beta = Effect size, SE = Standard Error, IAF = Interaction Allele Frequency, *P* = P value. Data were analysed using a linear regression with an interaction variable corrected for age, sex, and the first 10 principal components with Bonferroni correction for multiple testing. *P value<0.05.

Supplementary Table 3|The list of primers used for RT-qpcr.
