# Supplementary material for: The perineurium integrates leptin with its sympathetic outflow to protect against obesity
Source: Nat Metab. 2026 Jul 13;8(7):1563–82. doi: 10.1038/s42255-026-01555-3 (PMC13400315; doi:10.1038/s42255-026-01555-3)
Supplement: Supplementary file 2 — Reporting Summary [file 42255_2026_1555_MOESM2_ESM.pdf]

nature portfolio

Corresponding author(s): Ana Domingos

Last updated by author(s): Apr 28, 2026

Reporting Summary

Nature Portfolio wishes to improve the reproducibility of the work that we publish. This form provides structure for consistency and transparency in reporting. For further information on Nature Portfolio policies, see our Editorial Policies and the Editorial Policy Checklist.

Statistics

For all statistical analyses, confirm that the following items are present in the figure legend, table legend, main text, or Methods section.

n/a	Confirmed	
☐	☒	The exact sample size (n) for each experimental group/condition, given as a discrete number and unit of measurement
☐	☒	A statement on whether measurements were taken from distinct samples or whether the same sample was measured repeatedly
☐	☒	The statistical test(s) used AND whether they are one- or two-sided<br>*Only common tests should be described solely by name; describe more complex techniques in the Methods section.*
☐	☒	A description of all covariates tested
☐	☒	A description of any assumptions or corrections, such as tests of normality and adjustment for multiple comparisons
☐	☒	A full description of the statistical parameters including central tendency (e.g. means) or other basic estimates (e.g. regression coefficient) AND variation (e.g. standard deviation) or associated estimates of uncertainty (e.g. confidence intervals)
☐	☒	For null hypothesis testing, the test statistic (e.g. F, t, r) with confidence intervals, effect sizes, degrees of freedom and P value noted<br>*Give P values as exact values whenever suitable.*
☒	☐	For Bayesian analysis, information on the choice of priors and Markov chain Monte Carlo settings
☒	☐	For hierarchical and complex designs, identification of the appropriate level for tests and full reporting of outcomes
☒	☐	Estimates of effect sizes (e.g. Cohen's d, Pearson's r), indicating how they were calculated

Our web collection on statistics for biologists contains articles on many of the points above.

Software and code

Policy information about availability of computer code

Data collection	Single cell sequencing data were collected and initial processing were done by the Cell Ranger Single Cell Software Suite v.2.0.1.<br>Confocal Images were acquired with Zeiss LSM 780 Inverted Confocal Microscope. High-resolution images were acquired with a confocal laser scanning microscope, ZEISS LSM 980, equipped with an Airyscan detection unit.<br>Electron microscope images were acquired with a Ceta CCD camera (FEI, Thermo Fisher Scientific).<br>RNAscope images were acquired with an Olympus VS200 slide scanner (Olympus) and processed with Olympus OlyVIA 3.8 software.

Data analysis

Single cell data analysis: The Cell Ranger Single Cell Software Suite v.2.0.1 was used to perform sample de-multiplexing, alignment, filtering, and UMI counting. The cluster identities and filtered gene matrices generated by Cell Ranger software were used as input into the open-source R toolkit Seurat (v.4.1.2) to produce UMAP, feature plots, violin plots of the mean and variance of the mean and variance of gene expression density (Satija, R., Farrell, J. A., Gennert, D., Schier, A. F., & Regev, A. Spatial reconstruction of single-cell gene expression data. Nature biotechnology 33(5), 495-502, (2015)). For visualization of gene expression, count data for each run was denoised using a deep count autoencoder, DCA with default parameters (Eraslan, G., Simon, L.M., Mircea, M., Mueller, M.S, & Theis, F.J. Single-cell RNA-seq denoising using a deep count autoencoder. Nature communications 10 (1), 390 (2019)).

Confocal, RNAscope and Electron microscopy images were analyzed using FIJI software (Schindelin, J., Rueden, C.T., Hiner, M.C., & Eliceiri, K.W. The ImageJ ecosystem: An open platform for biomedical image analysis. Molecular reproduction and development 82 (7-8), 518-529 (2015)).

UK Biobank data were processed using SNPassoc R package (González, J.R. et al. SNPassoc: an R package to perform whole genome association studies. Bioinformatics 23 (5), 654-655(2007)) and KING (v2.2.3) (Manichaikul, A. et al. Robust relationship inference in genome-wide association studies. Bioinformatics 26 (22), 2867-2873 (2010)). Statistical analysis was performed using GraphPad Prism and SPSS.

For manuscripts utilizing custom algorithms or software that are central to the research but not yet described in published literature, software must be made available to editors and reviewers. We strongly encourage code deposition in a community repository (e.g. GitHub). See the Nature Portfolio guidelines for submitting code & software for further information.

Data

Policy information about availability of data

All manuscripts must include a data availability statement. This statement should provide the following information, where applicable:

- Accession codes, unique identifiers, or web links for publicly available datasets
- A description of any restrictions on data availability
- For clinical datasets or third party data, please ensure that the statement adheres to our policy

The raw single cell sequencing data of sympathetic ganglia supporting the findings in this study have been deposited in the GEO repository with the accession code GSE233163. The raw data of the public dataset re-analysed
in this study can be found in the GEO repository with the following accession codes: Tabula Muris (GSE109774 and GSE93374), murine sympathetic ganglia (GSE231767), dorsal root ganglia (GSE175421), nodose ganglia (GSE124312), sciatic nerve (GSE137870), Mouse Cell Atlas (GSE108097), Tabula Muris Senis (GSE132042), white adipose tissue (mouse; GSE17617 and human; GSE155960), brown adipose tissue (mouse; GSE207707 and human; E-MTAB-9199), human sympathetic ganglia (GSE241386) and Mouse Organogenesis Cell Atlas (GSE119945). The Seurat object containing the HypoMap dataset is available here: (https://doi.org/10.17863/CAM.87955).

Research involving human participants, their data, or biological material

Policy information about studies with human participants or human data. See also policy information about sex, gender (identity/presentation), and sexual orientation and race, ethnicity and racism.

Reporting on sex and gender

We performed genetic association analysis on the European population from the UK Biobank cohort. The analysis was performed in both male and female population.

Reporting on race, ethnicity, or other socially relevant groupings

N/A

Population characteristics

N/A

Recruitment

N/A

Ethics oversight

N/A

Note that full information on the approval of the study protocol must also be provided in the manuscript.

Field-specific reporting

Please select the one below that is the best fit for your research. If you are not sure, read the appropriate sections before making your selection.

☒ Life sciences ☐ Behavioural & social sciences ☐ Ecological, evolutionary & environmental sciences

For a reference copy of the document with all sections, see nature.com/documents/nr-reporting-summary-flat.pdf

Life sciences study design

All studies must disclose on these points even when the disclosure is negative.

Sample size

10 mice were pooled to obtain sufficient amount of cells from superior cervical ganglia and stellate ganglia in single cell RNA sequencing from control lean and diet induced obese mice groups.

	The sample size in experiments involving mice was estimated based on previous experiments (as optimal for in-vivo evaluation). The experiments involving genetically modified animals group sizes of minimum 3-4 animals were used. All ex-vivo and in-situ experiments were performed at least 2 times.
Data exclusions	There was no data exclusion.
Replication	Findings were reproduced as stated in method sections and figure legends. Experiments were performed at least two times independently.
Randomization	Randomization was used whenever possible.
Blinding	Data collection and analysis were not performed blind to the conditions of the experiments. Blinding was not performed due to the nature of the experimental setup; however, data quantification was performed using objective, standardized criteria to minimize bias.

Reporting for specific materials, systems and methods

We require information from authors about some types of materials, experimental systems and methods used in many studies. Here, indicate whether each material, system or method listed is relevant to your study. If you are not sure if a list item applies to your research, read the appropriate section before selecting a response.

Materials & experimental systems

n/a	Involved in the study	
☐	☒	Antibodies
☐	☒	Eukaryotic cell lines
☒	☐	Palaeontology and archaeology
☐	☒	Animals and other organisms
☒	☐	Clinical data
☒	☐	Dual use research of concern
☒	☐	Plants

Methods

n/a	Involved in the study	
☒	☐	ChIP-seq
☒	☐	Flow cytometry
☒	☐	MRI-based neuroimaging

Antibodies

Antibodies used	Antibodies were obtained from the following vendors: rabbit anti-tyrosine hydroxylase (TH) (Millipore, Cat #AB152) (1:500), chicken anti-TH (Aves Lab, Cat #TYH, lot TH1205) (1:500), goat anti-GFP (Abcam, Cat # ab6673) (1:1000), chicken anti-GFP (Abcam, Cat # ab13970) (1:1000), rabbit anti-caveolin-1 (CAV1) (Cell Signaling, Cat #D46G3) (1:1000), rabbit anti-glucose transporter (GLUT1) (Abcam, Cat #ab150299) (1:200), rabbit anti-TNFR1 (Invitrogen, Cat #PA595585) (1:500), rabbit anti-VE-cadherin (Life Technologies Ltd, Cat #361900) (1:100), goat F (ab) anti-mouse IgG (H+L) (Abcam, Cat #ab6668) (1:500), mouse anti-VEGFR2 (Santa Cruz Biotechnology, Cat #Sc-6251)(1:500), rabbit anti-NeuN (Abcam, Cat #ab177487)(1:500), rabbit anti-Des (Abcam, Cat#ab15200) (1:500), rat anti-CD31(BioLegend, Cat#102501) (1:2000), goat anti-rabbit IgG (H+L) secondary antibody, Alexa Fluor 647 (Invitrogen, Cat #A11010) (1:500), goat anti-rabbit IgG (H+L) secondary antibody, Alexa Fluor 546 (Invitrogen, Cat #A11035)(1:500), goat anti-chicken IgG (H+L) secondary antibody, Alexa Fluor 594 (Invitrogen, Cat #A11042) (1:500), goat anti-chicken IgY (H+L) secondary antibody, Alexa Fluor 647 (Invitrogen, Cat #A21449)(1:500), goat anti-chicken IgY (H+L) secondary antibody, Alexa Fluor 546 (Invitrogen, Cat #A11040)(1:500), goat anti-mouse IgG (H+L) secondary antibody, Alexa Fluor 594 (Invitrogen, Cat #A11005)(1:500), goat anti–chicken IgY (H+L) secondary antibody, Alexa Fluor 488 (Invitrogen, Cat #A-11039)(1:500), goat anti–rabbit IgG (H+L), Alexa Fluor 488, (Invitrogen, Cat #A11034)(1:500), donkey anti-goat IgG (H+L) secondary antibody, Alexa Fluor 647 (Invitrogen, Cat #A21447)(1:500), donkey anti-chicken IgY (H+L) secondary antibody, Alexa Fluor 647 (Stratech, Cat #703-605-155)(1:500), donkey anti-goat IgG (H+L) secondary antibody, Alexa Fluor 488 (Invitrogen, Cat #A11055)(1:500), donkey anti-chicken IgG (H+L) secondary antibody(1:500), Alexa Fluor 488 (Jackson ImmunoResearch, Cat #703-545-155)(1:500), donkey anti-rabbit IgG (H+L) secondary antibody(1:500), Alexa Fluor 546 (Invitrogen, Cat #A10040), DAPI (Invitrogen, Cat #D1306)(1:500).
Validation	All primary antibodies were validated by the manufacturers (see datasheets and manufacturer websites). Additional validation was supported by previous literature.

Eukaryotic cell lines

Policy information about cell lines and Sex and Gender in Research

Cell line source(s)	Primary human umbilical vein endothelial cells (HUVECs) (PromoCell, Catalog number C-12200)
Authentication	HUVECs (PromoCell, C-12200) were obtained from a commercial supplier. Cell line authentication was performed by the manufacturer; no additional authentication was performed by the authors.
Mycoplasma contamination	The cell line was tested negative for mycoplasma contamination.
Commonly misidentified lines (See ICLAC register)	N/A

Animals and other research organisms

Policy information about studies involving animals; ARRIVE guidelines recommended for reporting animal research, and Sex and Gender in Research

Laboratory animals	C57BL/6 mice, age 6-8 weeks, were purchased from Charles River.<br>LepR-cre mice (Leprtm2(cre)Rck; stock no. 008320) 6-8 weeks old and and Ob/Ob mice (stock no. 000632)were purchased from Jackson Laboratory.<br>Rosa26-LSL-ChR2-YFP mice (stock no. 012-569) 6-8 weeks old were purchased from Jackson Laboratory.<br>Adrb2flox/flox mice 6-8 weeks old was kindly provided by Gerad Karsenty, Columbia University, USA.
Wild animals	N/A
Reporting on sex	For metabolic phenotyping both male and female mice were used . Since there was no difference observed in the bodyweight of female conditional knock out and control mice, the rest of the experiments were conducted in male mice.
Field-collected samples	N/A
Ethics oversight	All experiments were conducted in accordance with the United Kingdom Animal Scientific Procedures Act 1986 under personal and project licences granted by the United Kingdom Home Office and approved by the local Department of Physiology Anatomy and Genetics (University of Oxford) ethical review committee.

Note that full information on the approval of the study protocol must also be provided in the manuscript.

Plants

Seed stocks	N/A
Novel plant genotypes	N/A
Authentication	N/A
